# Unidirectional diploid–tetraploid introgression among British birch trees with shifting ranges shown by restriction site‐associated markers

**DOI:** 10.1111/mec.13644

**Published:** 2016-05-11

**Authors:** Jasmin Zohren, Nian Wang, Igor Kardailsky, James S. Borrell, Anika Joecker, Richard A. Nichols, Richard J. A. Buggs

**Affiliations:** ^1^ School of Biological and Chemical SciencesQueen Mary University of LondonLondonE1 4NSUK; ^2^ QIAGEN Aarhus A/SSilkeborgvej 2PrismetAarhus C8000Denmark

**Keywords:** climate change, genotyping, hybridization, introgression, polyploidy

## Abstract

Hybridization may lead to introgression of genes among species. Introgression may be bidirectional or unidirectional, depending on factors such as the demography of the hybridizing species, or the nature of reproductive barriers between them. Previous microsatellite studies suggested bidirectional introgression between diploid *Betula nana* (dwarf birch) and tetraploid *B. pubescens* (downy birch) and also between *B. pubescens* and diploid *B. pendula* (silver birch) in Britain. Here, we analyse introgression among these species using 51 237 variants in restriction site‐associated (RAD) markers in 194 individuals, called with allele dosages in the tetraploids. In contrast to the microsatellite study, we found unidirectional introgression into *B. pubescens* from both of the diploid species. This pattern fits better with the expected nature of the reproductive barrier between diploids and tetraploids. As in the microsatellite study, introgression into *B. pubescens* showed clear clines with increasing introgression from *B. nana* in the north and from *B. pendula* in the south. Unlike *B. pendula* alleles, introgression of *B. nana* alleles was found far from the current area of sympatry or allopatry between *B. nana* and *B. pubescens*. This pattern fits a shifting zone of hybridization due to Holocene reduction in the range of *B. nana* and expansion in the range of *B. pubescens*.

## Introduction

Many species – especially of plants – have a history of whole‐genome duplication, leading to polyploidy (Grant 1981; Soltis *et al*. 2004; Stebbins 1971). Many polyploid species arise from the hybridization of two or more parental species and are known as allopolyploids. The establishment of a new polyploid species requires a degree of reproductive isolation from related diploid species (Levin 1975), but low levels of hybridization and introgression among species may occur (Abbott *et al*. 2013; Petit *et al*. 1999). Tracing patterns of introgression among species may help us understand their population histories and the dynamics of their evolution (Buggs 2007; Currat *et al*. 2008; Lamichhaney *et al*. 2015; The Heliconius Genome Consortium 2012). Polyploidy itself may affect the dynamics of introgression: Stebbins (1971) pointed out that introgression of alleles from a diploid to a tetraploid species is more likely to occur than vice versa. He argued (i) that triploid hybrids, which occur between diploid and tetraploid parents, mainly produce tetraploid progeny under open pollination (Stebbins 1971 p. 149) citing experimental evidence in *Dactylis* (Zohary & Nur 1959) and (ii) that unreduced gamete formation by diploids could sometimes give rise to hybrid tetraploids via fertilization of the gametes of tetraploid plants. In support of the idea of unidirectional introgression into tetraploids, Stebbins cited five examples of a widespread tetraploid species showing morphological similarity to local diploid species. A handful of studies have since provided evidence in favour of Stebbins’ hypothesis based on experimental data from wild populations of various plant species (e.g. Slotte *et al*. 2008; Chapman & Abbott 2010; Jørgensen *et al*. 2011; Han *et al*. 2015).

The genus *Betula* (birches) comprises about 60 species of trees and shrubs, among which polyploids are common (Ashburner & McAllister 2013; Wang *et al*. 2016) and hybridization is frequent (e.g. Ashburner & McAllister 2013; Thomson *et al*. 2015; Wang *et al*. 2014b; Palmé *et al*. 2004; Anamthawat‐Jónsson & Tómasson 1990; Anamthawat‐Jónsson & Thórsson 2003; Anamthawat‐Jónsson *et al*. 2010). The genus is widespread in the Northern Hemisphere with species ranging from north of the Arctic Circle (*B. nana*) to the subtropics (*B. alnoides*). In Britain, there are three native birch species: diploid *B. nana* (dwarf birch), diploid *B. pendula* (silver birch) and allotetraploid *B. pubescens* (downy birch). *Betula nana* belongs to section *Apterocaryon* (subgenus *Betula*), and *B. pendula* and *B. pubescens* are of section *Betula* (subgenus *Betula*; Ashburner & McAllister 2013). *B. pendula* is thought to be one parent of *B. pubescens*, with the other parent hypothesized to be *B. humilis* (Walters 1968; Howland *et al*. 1995), though as yet not proven (Anamthawat‐Jónsson *et al*. 2010). Analyses of pollen records suggest that *B. nana* was once widespread throughout Britain (Wang *et al*. 2014b). Today, however, *B. nana* has retreated into mountainous areas of Scotland, while *B. pubescens* and *B. pendula* are widespread. Studies on other tree species suggest that such range shifts can be strongly affected by climate change (Lenoir *et al*. 2008; Zhu *et al*. 2012).

Hybridization has been shown to occur between *B. pendula* and *B. pubescens* (e.g. Palmé *et al*. 2004; Wang *et al*. 2014b) and between *B. nana* and *B. pubescens* (e.g. Anamthawat‐Jónsson & Tómasson 1990; Anamthawat‐Jónsson & Thórsson 2003; Anamthawat‐Jónsson *et al*. 2010; Wang *et al*. 2014b). A ‘triploid block’ (Marks 1966), as reported in other interploidal crosses (e.g. Woodell & Valentine 1961; Lafon‐Placette & Köhler 2016), has not yet been shown to prevent hybridization among *Betula* species. However, it has been suggested that low temperatures in the north facilitate hybridization in *Betula* (Eriksson & Jonsson 1986), while an asymmetric pattern of introgression previously described between *B. nana* and *B. pubescens* suggests that backcrossing of hybrids mainly occurs with *B. pubescens* rather than with *B. nana* (Wang *et al*. 2014b; Eidesen *et al*. 2015). Hybrids of *B. pubescens* and *B. pendula* have been reported very frequently and are often described as *B. *x *intermedia* (Kenworthy *et al*. 1972).

Anamthawat‐Jónsson & Tómasson (1990) compared chromosome complements in tetraploid *B. pubescens*, diploid *B. nana* and their hybrids from Iceland. They found triploid hybrids between the two species showing variable viability and fertility, and some were morphologically very similar to a parental species (Thórsson *et al*. 2007). They suggested that these triploids make introgression from *B. nana* into *B. pubescens* possible, and confirmed this using gene mapping on chromosomes and genomic in situ hybridization (Anamthawat‐Jónsson & Thórsson 2003). Karlsdóttir *et al*. (2009, 2014) reported evidence for Holocene hybridization between *B. nana* and *B. pubescens* in Iceland using pollen analysis from peat profiles, while Eidesen *et al*. (2015) obtained evidence for hybridization between them based on surveys of AFLP and plastid DNA variation in populations across Europe and North America. Eidesen *et al*. (2015) further noted that AFLP introgression from *B. nana* to *B. pubescens* increased at more northerly latitudes. Palmé *et al*. (2004) found extensive chloroplast haplotype sharing among *B. nana*,* B. pendula* and *B. pubescens* in Russia and Europe, indicative of hybridization, while Wang *et al*. (2014b) obtained evidence for bidirectional introgression between tetraploid *B. pubescens* and the diploid species, *B. nana* and *B. pendula*, in Britain based on an analysis of twelve microsatellite loci. In addition, Wang *et al*. (2014a, b)detected latitudinal clines in level of introgression within *B. pubescens*.

This discovery of bidirectional introgression was unexpected, given the ploidy level differences among the three British birch species, but the cline of *B. nana* alleles penetrating deep into the range of *B. pubescens* provided striking confirmation of the hypothesis that trails of introgression can reflect past hybrid zone movements due to climate change. Wang *et al*. (2014b) argued that because shared alleles between *B. nana* and *B. pubescens* formed a cline, they were not the result of incomplete lineage sorting, as this should not elicit a geographical signal (Barton 2001), but due to introgression. Furthermore, it was reasoned that the length of the cline of *B. nana* alleles into *B. pubescens* was too great to be explained by gene flow only from the current range of *B. nana*, but could be explained in terms of a larger distribution of *B. nana* in the past and a gradual retreat of this species due to climate change and habitat loss, accompanied by hybridization with advancing populations of *B. pubescens*. To test the trustworthiness of the clines found in the microsatellite study and ascertain whether the results for the twelve loci are representative of genomewide patterns of introgression, we here present a study that examines variation for thousands of RAD markers among the three species using a subset of individuals from Wang *et al*. (2014b).

Our study required accurate genotyping of thousands of markers in many individuals, which is challenging in polyploids (reviewed in Dufresne *et al*. 2014). Whereas in a diploid, the presence of two alleles can be unambiguously assigned to an exact genotype (e.g. ‘AT’), in a tetraploid, the presence of two alleles can be due to any of three possible genotypes with different allele dosages (e.g. ‘AAAT’, ‘AATT’ and ‘ATTT’). The number of possible genotypes increases for levels of polyploidy higher than tetraploid. Furthermore, it is possible for a locus in a polyploid to be triallelic or even tetra‐allelic. Thus, while many studies have analysed introgression at genomewide SNP markers among diploid species (e.g. Lam *et al*. 2010; Hohenlohe *et al*. 2011; Amish *et al*. 2012; Stölting *et al*. 2013; Rheindt *et al*. 2014; Hand *et al*. 2015; Christe *et al*. 2016; Kenney & Sweigart 2016), only a few studies have analysed introgression for SNPs between diploid and polyploid species (e.g. Arnold *et al*. 2015; Clark *et al*. 2014, 2015).

Few tools exist that use NGS read‐count data to call genotypes with allele dosages in polyploids. Uitdewilligen *et al*. (2013) used ‘FreeBayes’ (Garrison & Marth 2012) to genotype biallelic SNPs with dosage information in autotetraploid potato. Blischak *et al*. (2015) developed the r package ‘polyfreqs’ to genotype autopolyploids from read counts at biallelic SNP loci where each locus has no missing data, while Arnold *et al*. (2015) used ‘GATK’ (McKenna *et al*. 2010) to genotype biallelic SNPs in autotetraploid *Arabidopis arenosa*. Other recent methods such as ‘HANDS’ (Mithani *et al*. 2013), ‘PolyCat’ (Page *et al*. 2013) and ‘SNiPloid’ (Peralta *et al*. 2013) assign SNP alleles to specific subgenomes of allopolyploids, relying on data from known diploid progenitors. We decided to construct our own pipeline for the current paper to genotype tetraploid *B. pubescens* as: (i) it is an allotetraploid and therefore may have loci that are tri‐ or even tetra‐allelic; (ii) we are using RAD markers and are thus likely to have high levels of missing data; and (iii) we do not have genome data from its diploid progenitors, which are still not known with certainty.

Here, we present a new RAD‐sequence data set for populations of *Betula nana*,* B. pendula* and *B. pubescens* from across Britain. We identify variant loci using the CLC Genomics Workbench and use read‐count data to confirm the ploidy level of each individual applying a method similar to one used by Arnold *et al*. (2015). Using a custom script (Zohren *et al*. 2016), we use read‐count data to infer genotypes of variable loci in 37 *B. nana*, 37 *B. pendula* and 131 *B. pubescens* individuals. We then analyse patterns of genetic differentiation and introgression across 51 237 variable loci among the three species, comparing these results with previous findings based on twelve microsatellite markers (Wang *et al*. 2014b).

## Materials and methods

### Sampling

**Figure 1:**
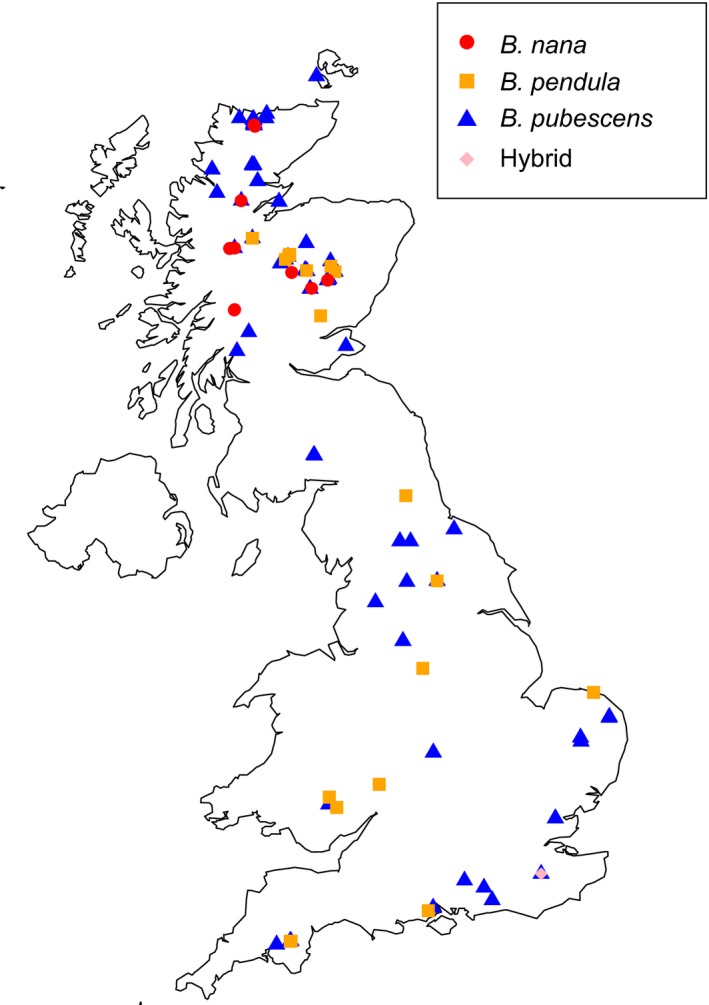
Collection locations of the 213 *Betula* samples used for restriction site‐associated sequencing. Circles = *B. nana*, squares = *B. pendula*, triangles = *B. pubescens*, diamond = hybrid. Map created with r packages ‘maps’ and ‘mapdata’.

We used samples which had been collected as leaves and twigs from wild *Betula* populations across Britain between April 2010 and August 2013 and pressed (Wang *et al*. 2014a,b). An initial identification of the species was based on leaf morphology according to the standard guide for UK birch identification (Rich & Jermy 1998), including the Atkinson discriminant function (Atkinson & Codling 1986; Wang *et al*. 2014a). A set of 205 individuals was used in the present RAD study: 37 *B. nana*, 37 *B. pendula* and 131 *B. pubescens* individuals. A map of collection locations of samples used for RAD analysis is provided in Fig. 1, and detailed information on sample sites is provided in Table S1 (Supporting information).

### DNA sequencing

Genomic DNA was extracted from dried cambial tissue and leaves using a modified cetyltrimethylammonium bromide (CTAB) protocol (Wang *et al*. 2013). Library preparation and RAD sequencing using a single digest PstI library (cut site 5′–CTGCAG–3′) was carried out by the GenePool genomics facility in the University of Edinburgh. For an initial set of 16 samples, 96‐bp paired‐end reads were produced (see Wang *et al*. 2013); for the remaining 197 samples, 42‐bp‐long single‐end reads were generated (sequenced in two batches of 177 and 20 samples). Eight samples were technically replicated.

### Read mapping and variant calling

To create a consistent data set, only the first read of the paired‐end reads of the first sequencing batch of 16 samples was used. These reads were trimmed to match the length of the single‐end reads (i.e. all reads that were analysed were 42 bp long). All 205 samples, eight of them technically replicated, were mapped to a reference sequence of *Betula* RAD loci and their flanking regions in the *B. nana* genome (Wang *et al*. 2013) using the CLC ‘Map Reads to Reference’ tool (CLC bio, Qiagen Aarhus 2012). Reads that mapped equally well to more than one position on the reference sequence were ignored. To facilitate this mapping, the 115 142 individual contigs in the reference sequence were concatenated with 50 ‘N's separating them, resulting in a 106‐Mbp‐long sequence. The 213 individual mappings were merged into one (using the CLC ‘Merge Read Mappings’ tool), which was further locally realigned with the CLC ‘Local Realignment’ tool. This reduced the number of mismappings and generally improved the quality of the read mapping using cross‐read information (CLC bio, Qiagen Aarhus 2013). Next, variants were called on the locally realigned merged read mapping. The CLC ‘Low Frequency Variant Detection’ tool (CLC bio, Qiagen Aarhus 2014) was used to create a global variant track that combines variants found in all samples (some of which might only be at very low frequency). The variant caller relies on a statistical model and accounts for sequencing errors. To validate the number of variants, the CLC ‘Fixed Ploidy Variant Detection’ tool (CLC bio, Qiagen Aarhus 2014) was run on the same data, using default parameters and setting the ploidy parameter to four.

To trace back each sample's locus configuration, the ‘Identify Known Mutations from Sample Mappings’ tool from the Biomedical Genomics Workbench (CLC bio, Qiagen Aarhus 2015) was used. This takes the global variant track and the individual read mappings as input and looks up every variant position in each sample. The output is one variant table per sample containing the number of reads supporting each variant, among many other values. This approach (calling variants on a combined mapping rather than on each individual and then going back to the individual's positions) allowed us to account for rare variants and reduced computing time. Detailed parameter settings and version numbers for each of the CLC tools are provided in Table S2 (Supporting information).

The variants were then filtered to include only single nucleotide variations and single base deletions to facilitate analyses (hereafter referred to as SNVs). A flow chart of this analysis pipeline is presented in Fig. S1 (Supporting information).

### Allelic ratios at heterozygous sites

**Figure 2:**
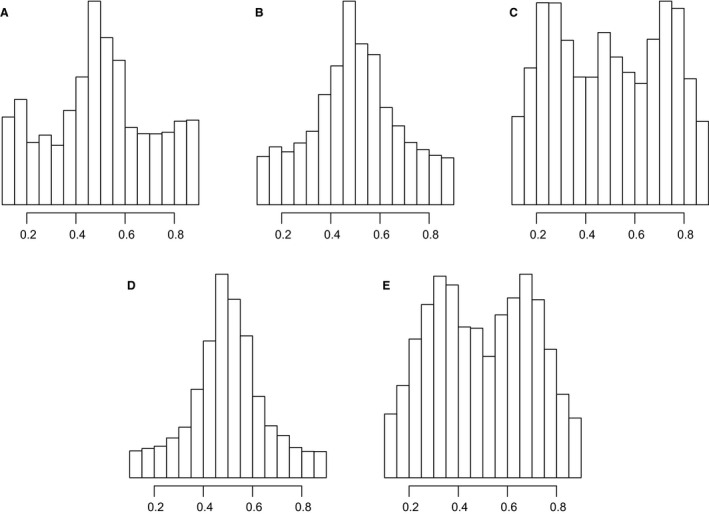
Distribution of read count ratios for heterozygous sites covered by at least 30 reads. (A) All *B. nana* individuals. (B) All *B. pendula* individuals. (C) All *B. pubescens* individuals. (D) Sample number 574, which we conclude is an autotetraploid of *B. pendula* (see main text). (E) Sample number 1173, which we conclude is a triploid *B. pubescens* x *B. pendula* hybrid (see main text). Figure created in r.

To assess the ploidy of the samples using the RAD data, we plotted the distribution of allele ratios from read counts at heterozygous sites with at least 30× coverage (Fig. 2). A diploid sample should have one peak around 0.50, a triploid should have peaks near 0.33 and 0.66, and a tetraploid should have peaks close to 0.25, 0.50 and 0.75 (due to the greater number of possible heterozygotes). We initially examined the histograms of allelic counts at heterozygous biallelic loci in individuals thought to be diploid. This distribution was compared with the binomial distribution with mean 0.5. The dispersion of frequencies around the mean was consistently larger than the binomial expectation, presumably due to subtle biases in the number of counts sequenced at each locus, generated by the extraction, library preparation and sequencing methods. We therefore modelled the distributions as beta‐binomial – the distribution in which the mean for each locus is drawn from a beta distribution, specified by an expectation (overall mean) *p* and a correlation coefficient ρ. The value of ρ determines the dispersion of the locus‐mean around the expectation.

In the case of a polyploid individual, the counts were assumed to be a mixture of beta‐binomial distributions, depending on the number of alleles at a heterozygous locus. In our study, we suspect that most nondiploids would be tetraploids, so the possible genotypes at a heterozygous locus (alleles A or B) would be, ABBB (¼ A), AABB (½ A) or AAAB (¾ A), hence *p* ∈ {¼, ½, ¾}. More generally, in a polyploid individual with *k* haploid chromosome sets, *p* ∈ {1*/k … (k‐*1*)/k*}. For a diploid individual, *p* corresponds to the single value *p *=* *0.5, because half the expected reads are of each allele.

The log‐likelihood of the observations, *L*, was calculated for each individual as:

$$L=\sum_{l}\text{log}\;(\sum_{p}m_{p}\beta b\left(x_{l},n_{l},p,\rho \right)),$$

where the outer sum is over the *l* loci which have been identified as heterozygous in the individual concerned. The parameter *m*_*p*_ is the proportion of the loci at which the expected frequency of reads would be *p*. The function *ßb*() represents the beta‐binomial density function. It has four parameters: *x*_*l*_ is the count of reads of an allele at locus *l, n*_*l*_ is the total number of reads at locus *l*,* p* is the expectation of the beta‐binomial distribution, and ρ is the correlation coefficient. The data were censored to include only the range 0.1–0.9 in order to exclude counts from loci that were in fact homozygous, but appeared heterozygous due to mistyping errors. The *ßb*() function was modified accordingly (by dividing by the total density in the uncensored range) and implemented in r with the ‘vgam’ package (Yee & Wild 1996; Yee 2015).

The r function ‘mle’ was used to obtain the maximum‐likelihood combination and confidence intervals of the parameters *m*_*l*_ and ρ for each individual. Results were obtained for the diploid and polyploid calculations. The relative support for an individual being a diploid was calculated using the Akaike information criterion (AIC; Akaike 1974) function to compare the maximum‐likelihood models for the diploid case with other ploidy levels. The script is available in the Dryad Digital Repository (Zohren *et al*. 2016).

### Genotyping

To genotype each locus in each individual, we wrote a custom script (Zohren *et al*. 2016), which uses as inputs read counts and base qualities extracted from the CLC variant calling, and the ploidy level of the sample estimated using its allelic ratios (see above). We assume unbiased and independent read sampling at each locus during sequencing, mapping and read counting. The script uses model tables which have one row per possible allelic dosage in a given ploidy level: for a diploid, these are 2:0 and 1:1, and for a tetraploid, these are 4:0:0:0, 3:1:0:0, 2:2:0:0, 2:1:1:0 and 1:1:1:1.

The reads that support different allelic variants at a locus within an individual are sorted in descending order of frequency. Their call qualities, expressed as probability of an error from a simple conversion of the average phred quality score of the allelic variant, are sorted along with the read numbers. Only loci with coverage thresholds of at least five reads per ploidy level (i.e. threshold of 10 in the diploids and 20 in the tetraploids) and an upper threshold of 200 reads are used in subsequent analysis (the effect of different coverage thresholds on the number of SNVs is shown in Table S3, Supporting information).

The likelihood formula used in the genotyping script is then constructed as follows: Let *n* be the chosen ploidy level; *x* a vector of counts of reads observed for each allele, sorted in descending order (if *length(x)* > *n*, it is truncated to *n* on the right); *q* the corresponding average base quality for each called allele on a phred scale, ordered as *x*;* m*_*i*_ a vector of numbers, sorted in descending order, corresponding to a particular dosage model for a given ploidy level (*sum(m)* = *n*,* length(m)* is made equal to *n*, by padding with ‘0’, if a model specifies fewer alleles than a ploidy level, i.e. a triploid homozygote is represented as 3:0:0 and a biallelic locus in a triploid genome as 2:1:0); and *s* a subset of indices in {*i*}, where *m*_*i*_ > 0, and $\overset{¯}{s}$ is its complement, that is positions in the model representation where no alleles are expected. The data likelihood is then calculated as two parts:

- The polynomial probability where a model expects read counts:
  $$L_{1}=\frac{(\sum_{s}x_{s})!}{\prod_{s}\left(x_{s}\right)!}\prod_{s}{\left(\frac{m_{s}}{n}\right)}^{x_{s}}$$

- And the error probability where reads are present, but not expected from a model, converted from a phred score:
  $$L_{2}=\prod_{\overset{¯}{s}}p_{\overset{¯}{s}}^{x_{\overset{¯}{s}}},\;\mathrm{where}\;\;p=10^{-q/10}$$

The total likelihood is then *L *= *L*_1_ * *L*_2_. For computational convenience, the log‐likelihood is calculated in the script (i.e. products become sums). The Bayesian information criterion (BIC; Schwarz 1978) is then computed to determine the best fitting model.

Our final genotype calls excluded the following: individuals with fewer than one million raw reads, variants other than SNVs or deletions, sites with a coverage below 10 and above 200 reads, individuals with >50% missing data, and loci that were not present in at least 80% of individuals.

### Population structure

The analysis of admixture among the three species was performed in structure version 2.3.4 (Pritchard *et al*. 2000). Diploids were coded as if tetraploid (i.e. four rows per individual with the last two only containing missing data) to allow a simultaneous analysis of the mixed ploidy data set. It was run with a burn‐in period of 100 000 and a further 100 000 repeats using the admixture model, correlated allele frequencies and the number of assumed populations (‘*K*’) set to three. This was repeated 20 times. An admixture plot was created using distruct version 1.1 (Rosenberg 2004) after using structure harvester (Earl 2012) and clumpp (Jakobsson & Rosenberg 2007) to combine the output of the 20 repeats. Other values of *K* (one to five) were tested in addition to the main analysis with *K *=* *3. A principal component analysis (PCA) was carried out using a combination of the ‘adegenet’ r package version 2.0.0 (Jombart 2008), the ‘missmda’ r package version 1.8.2 (Josse & Husson 2016) to impute missing data and the ‘prcomp’ function from the r ‘base’ package (R Core Team 2015). The results presented in Fig. S2 (Supporting information) justify the use of imputed values, as individuals with a high proportion (>10%) of missing data, which are highlighted, still seem to cluster well into the groups. For the computation of the PCA, the genotype information was transformed into allele frequencies (normalized for the ploidy level), and thus, only biallelic variants could be used (95% of the full data set). *F*_ST_ values were calculated based on allele frequencies with the ‘hierfstat’ r package version 0.04‐22 (Goudet & Jombart 2015) and a custom implementation (Zohren *et al*. 2016).

The geographical cline in the direction of the introgression pattern was examined using a mixed effects model on arcsine‐transformed estimates of admixture proportions (returned by structure), the slope as a fixed effect, and the population modelled as a random effect. The latter allows for genetic drift of each population away from the overall trend. This was implemented in r using the ‘lme’ function (Pinheiro *et al*. 2015).

### Comparison of RAD and microsatellite data

structure was rerun on 177 individuals, for which previously published microsatellite markers (Wang *et al*. 2014b) as well as the present RAD data were available. It was set to a burn‐in period of 10 000 and a further 100 000 repeats using the admixture model, correlated allele frequencies, and *K *=* *3. We randomly selected 1000 RAD variants to compare them to the twelve microsatellite loci. The distribution of *Q*‐values from each of the runs was plotted in r (R Core Team 2015) for a direct comparison of the amount of admixture estimated from the microsatellite markers and RAD sequencing, respectively.

## Results

### Read mapping and variant calling

The individual read mappings resulted in 33.05% to 86.7% of mapped reads per individual. Five individuals (one *B. nana*, two *B. pendula* and two *B. pubescens*) were excluded because they each had less than one million raw reads (2400–165 500). So the remaining data set consisted of 208 individuals (with 6.6 million raw reads on average). A further eight individuals were discarded because they had more than 50% of missing data in the data set of loci that were covered by at least 80% of the samples. Their missing data content ranged from 50.6% to 85.2%, and one *B. nana*, one *B. pendula* and six *B. pubescens* individuals were affected. This reduced the data set to 200 individuals (including six technical replicates; two of the initial eight replicates were filtered out).

In the merged mapping with data from all individuals, 1.09 billion reads (79.4%) mapped to the reference and almost four million variants were called. Over 2.8 million variants were supported by at least five reads and almost 2.1 million were supported by at least ten reads. The CLC ‘Low Frequency Variant Detection’ tool calculated that 99.7% of the four million variants were called with a probability of >90% and 2.7 million (68.6%) with a probability of 100%. As expected, fewer variants (1.7 million) were found with the CLC ‘Fixed Ploidy Variant Detection’ tool, as this tool is focused on specificity rather than sensitivity and was not designed for the detection of low frequency variants.

### Allelic ratios at heterozygous sites

Bar charts of allele ratios at heterozygous sites (Fig. S3, Supporting information) confirmed the expected ploidy level for the vast majority of samples, with diploids showing a peak around 0.50 and tetraploids showing peaks near 0.25, 0.50 and 0.75. There were two exceptions (Fig. 2D,E). One individual (sample ID 574), which had previously appeared to be unusual in its morphology and RAD loci (Wang *et al*. 2013), had an excess of 0.50 over 0.25 and 0.75 ratios, suggesting that it is a diploid, even though its genome size is that of a tetraploid (Wang *et al*. 2013). We conclude from this that it is a recent autotetraploid. Another individual (sample ID 1173) showed peaks around 0.33 and 0.66, indicating that it is a triploid. It had been initially classified as a *B. pubescens* based on morphology. On the basis of the beta‐binomial model, all but two individuals (sample IDs 2347 and 2354) were correctly assigned (when compared to a visual assessment of the histograms, the plants’ morphology, the microsatellite results and the clustering results of the present study). These two were samples with rather few variable sites and seemingly very heterozygous. The AICs resulting from the beta‐binomial model comparisons are reported in Table S1 (Supporting information).

### Genotyping

After filtering (> one million raw reads, only SNVs, coverage between 10× and 200×, <50% missing data; see above), 541 080 variants were present in at least one individual and covered by 66 reads on average. Of these, 59 were present in all 200 individuals: too small for population analyses. Instead, we used as the basis of our population analyses variants present in at least 80% of individuals, of which there were 51 237. Subtracting 687 variants that only had one allele in this data set (when eight individuals with >50% missing data had been removed), we had 50 550 variants of which 49 025 were biallelic, 1484 were triallelic and 41 were tetra‐allelic.

### Population structure

**Figure 3:**
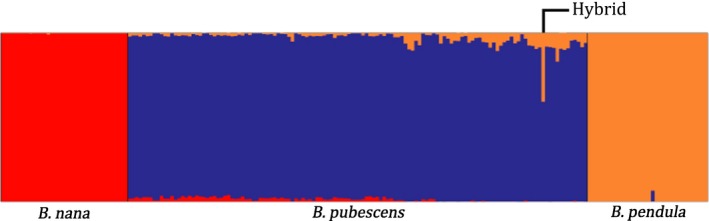
Estimated genetic admixture of 200 *Betula* samples at 51 237 variant loci. Each individual is represented by a vertical line and species are separated by different colours and a black vertical line. Within species, the samples are sorted by latitude from left (north) to right (south). Results are taken from running structure with 100 000 repeats in addition to a 100 000 burn‐in period and *K *= 3. Red = *B. nana*, orange = *B. pendula*, blue = *B. pubescens*. Figure created with ‘distruct1.1’.

The results of the PCA (Fig. 3) based on genotype calls for 49 025 biallelic loci in 200 individuals clearly indicated three tight clusters corresponding to the three *Betula* species in the data. The individual previously identified as triploid (1173) fell between the *B. pubescens* and *B. pendula* clusters and is therefore likely of hybrid origin. The first principal component (PC), which accounts for 26.9% of the variation in the data set, differentiates well between the three species, with *B. nana* widely separated from the other two species and *B. pubescens* somewhat intermediate, though much closer to *B. pendula*. The second PC, accounting for 9.1% of the variation in the data set, widely separates *B. pubescens* and *B. pendula*, with *B. nana* intermediate between them. The putatively autotetraploid individual 574 fell into the *B. pendula* cluster in the PCA.

**Figure 4:**
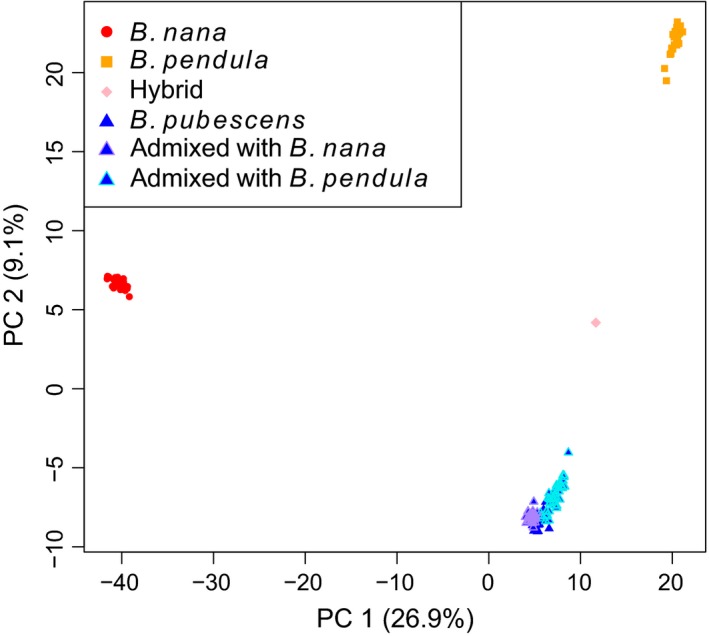
Principal component analysis of 200 *Betula* samples at 49 025 biallelic variant loci. Colours used correspond to the attributes of individuals in the structure analysis: red circles = *B*. *nana*, orange squares = *B*. *pendula*, blue triangles = *B*. *pubescens*, pink diamond = hybrid individual, *B*. *pubescens* individuals admixed with at least 2% *B*. *nana* (blue triangles with purple outline) or at least 2% *B*. *pendula* (blue triangles with cyan outline). Figure created in r.

The structure plot (Fig. 4) was generated setting *K* to three, because three clear clusters appeared in the PCA and showed clear isolation of the species, based on the 51 237 loci. Results showing the estimated admixture levels with *K* set to one to five are shown in Fig. S4 (Supporting information) together with the log‐likelihood values of each *K*. In the diploid species, *B. nana* and *B. pendula*, very little introgression was detected (highest admixture levels of 0.74% and 6.4%, respectively). More admixture was estimated in the tetraploid *B. pubescens*, showing introgression from both *B. nana* and *B. pendula*, with highest admixture values of 3.8% and 16.9% (excluding the potential hybrid, see below), respectively (Fig. 4). These individuals are also positioned on the periphery of the *B. pubescens* cluster in the PCA plot (Fig. 3, individuals with at least 2% admixture are highlighted). According to the structure estimate, plant 1173, the potential triploid hybrid, is made up of 59.3% *B. pubescens* and 40.7% *B. pendula*. Plant 574, the putative autotetraploid, was found to be *B. pendula* with no introgression from either *B. nana* or *B. pubescens*.

Among the three species, we found a high level of genetic differentiation, with a global mean *F*_ST_ of 0.40, suggesting that genetic variance among the species is almost as great as genetic variance within them. Within species, *F*_ST_ values among populations with at least three individuals are 0.08, 0.03 and 0.01 for *B. nana*,* B. pendula* and *B. pubescens*, respectively, indicating greater population structure in *B. nana*, probably due to smaller and more widespread populations. The pairwise comparisons between the three species at each locus (Fig. S5, Supporting information) showed many F_ST_ outliers (4112 for *B. nana*–*B. pendula*; 6142 for *B. nana*–*B. pubescens*; and 5230 for *B. pendula*–*B. pubescens*). The difference between the two diploid species *B. nana* and *B. pendula* is the greatest (mean of 0.17), in contrast to *B. nana*–*B. pubescens* (mean of 0.07) and *B. pendula*–*B. pubescens* (mean of 0.05). These mean figures are based on the same set of loci for all three comparisons, and so include fixed alleles in some cases, causing lower values than the global F_ST_ calculated above. The pattern of greatest differentiation between *B. nana* and *B. pendula* fits well with the results from the structure analysis (Fig. 4).

**Figure 5:**
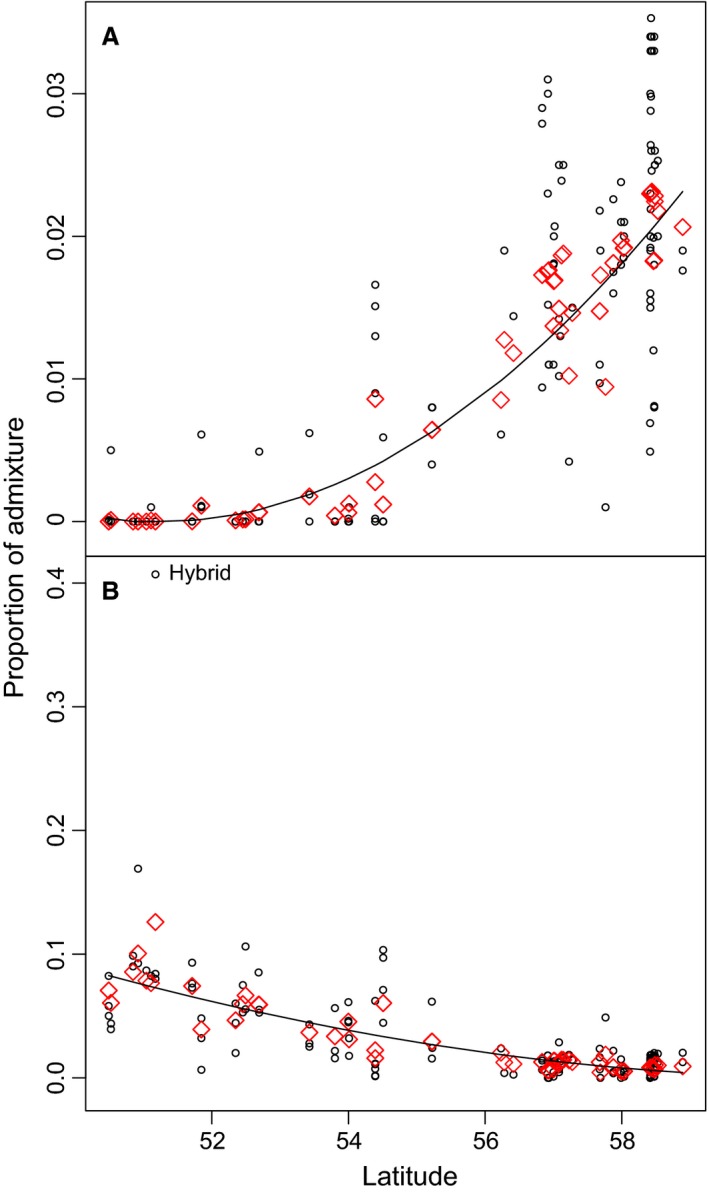
Cline analysis of admixed *B. pubescens* individuals. An arcsine transformation of the structure results and a mixed effects model were used. Individual admixture proportions are shown as black circles and red diamonds represent population means as fitted by the model. (A) Admixture from *B. nana*. (B) Admixture from *B. pendula*. Figure created in r.

We observed a geographical trend of the introgression pattern within the *B. pubescens* individuals. The more northerly individuals show more introgression from *B. nana*, whereas the individuals towards the south are increasingly admixed with *B. pendula* (Figs 4 and 5). The results in both cases were highly significant (*P*‐values of 1.1 × 10^−21^ and 3.7 × 10^−13^ for *B. nana* and *B. pendula* individuals, respectively).

The results for the technical replicates were concordant with each other in both the PCA and structure analysis. The biggest difference in the PCA between any two technical replicates was 0.32 units (on PC 2), and the biggest difference in the amount of admixture between any two replicates detected with structure was 0.3%.

### Comparison of RAD and microsatellite data

**Figure 6:**
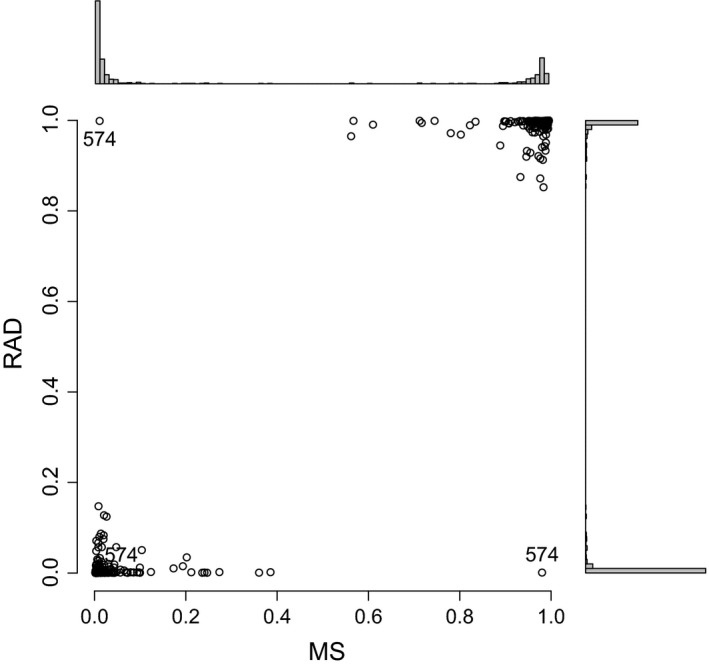
Comparison of *Q*‐values from microsatellite and restriction site‐associated (RAD) data. Genetic admixture for 177 *Betula* samples was estimated using structure on twelve microsatellite loci and 1000 RAD variants. *Q*‐value pairs of plant 574 are labelled, and distribution of *Q*‐values shown as histograms on the outer axes. Figure created in r.

We directly compared 1000 randomly selected loci from the RAD data presented here with the twelve previously published microsatellite data set (Wang *et al*. 2014b), by rerunning structure on 177 individuals for which we had both data. An alignment of the RAD and microsatellite structure plots is shown in Fig. S6 (Supporting information). The microsatellite data produced greater estimates of introgression among all three species, as visualized in a scatterplot of the *Q*‐values from both data sets (Fig. 6). The correlation among all three species was 0.74 (Spearman rho) and highly significant (*P* = 1.1 × 10^−93^). For just the *B. pubescens* individuals, rho was 0.68; for *B. pendula*, 0.59; and for *B. nana*, 0.50. In addition, the individual identified as an autotetraploid (sample ID 574, see above) was identified as being *B. pubescens* with the microsatellite markers (with 2.8% introgression from *B. nana* and 3.7% introgression from *B. pendula*), but appeared to be a *B. pendula* in the RAD data set (with 0.04% introgression from *B. nana* and 0.1% from *B. pubescens*; also labelled in Fig. 6). These admixture values differ to those presented above due to the smaller number of RAD loci used in this analysis (1000 vs. 51 237). To ensure that this individual had not been mislabelled, we resampled the tree, re‐extracted DNA and repeated the analyses. The results remained unchanged. Unfortunately, there is no microsatellite data for the triploid hybrid individual (1173) available so we cannot make a comparison for this individual.

## Discussion

Genomewide single nucleotide variants in birches throughout Britain clearly and unambiguously distinguish the three species *Betula nana*,* B. pendula* and *B. pubescens*. The structure estimates of admixture proportions suggest predominantly unidirectional gene flow has occurred into the tetraploid species *B. pubescens* from the two diploid species, *B. nana* and *B. pendula*. This gene flow has produced significant clines, with greater introgression from *B. nana* in the north and greater introgression from *B. pendula* towards the south. We found evidence for very little introgression into *B. nana* and *B. pendula*. We found one tree that appears to be a triploid hybrid between *B. pendula* and *B. pubescens* and another tree that could be a *B. pendula* autotetraploid.

The individuals genotyped in the present study are mainly a subset of those included in a previous study using twelve microsatellite loci (Wang *et al*. 2014b). The 51 237 variants analysed with RAD data generate much tighter clusters of the three species than the twelve microsatellites in principal coordinate analysis, and the clusters are more widely spaced from one another. Both the microsatellite data and the RAD data showed clines of introgression into *B. pubescens* from the other species, but the slopes of the clines are more significant for the RAD data; this is especially the case for southerly introgression from *B. pendula* into *B. pubescens*, which appears to be more discernible in the RAD data than in the microsatellite data. The RAD data contrasts with the microsatellite data in showing very little to no introgression into the two diploid species. One individual (574) which we identified as an autotetraploid based on counts of allele ratios and a flow cytometry measurement (Wang *et al*. 2013) is clustered with *B. pendula* using RAD markers but with *B. pubescens* using microsatellite markers. This individual also has unusual leaf morphology (Wang *et al*. 2013) and deserved further attention to resolve its parentage and species identification.

The differences seen between the microsatellite and RAD data sets may be due to several different possible causes: (i) the RAD variants are much greater in number and more widely distributed throughout the genome than the microsatellites, which is likely to have produced a more comprehensive and accurate measure of introgression; (ii) a subset of the very large number of RAD variants may be closely linked to loci under selection (as suggested by the thousands of F_ST_ outliers found among species), whereas such effects are *prima facia* less likely with the smaller number of microsatellites; (iii) microsatellite mutation rates are higher than SNP mutation rates, so microsatellite introgression may reflect more recent hybridization than SNP introgression (Ellegren 2000), perhaps due to human planting of saplings of different birch species closer to one another than would be common via natural propagation from seed (Wang *et al*. 2014b); (iv) the 51 237 variable loci are better able to distinguish *B. pubescens* variants from *B. pendula* variants (the PCAs for both the RAD and microsatellite data show that the difference between *B. nana* and the other two species is greater than the difference between *B. pendula* and *B. pubescens*, and the RAD data set provides much sharper resolution of *B. pendula* and *B. pubescens*), which will therefore allow better detection of introgression between them; (v) it may be that the different methods used for genotyping have systematically favoured calling SNP heterozygotes in tetraploids and microsatellite heterozygotes in the diploids, leading to an appearance of lower introgression into diploids in the RAD data; (vi) homoplasy may be more common in the microsatellite markers (Li *et al*. 2002), which would be expected to increase estimated rates of introgression bidirectionally (not unidirectionally) as we find. If the RAD data have provided greater precision than the twelve microsatellites, this pattern fits well with Stebbins’ (1971) argument that introgression should be unidirectional from diploids to tetraploids (see Introduction).

Introgression has been demonstrated for several natural systems using RAD markers (e.g. The Heliconius Genome Consortium 2012; Lamer *et al*. 2014; Combosch & Vollmer 2015; Eaton *et al*. 2015; Stankowski & Streisfeld 2015). Three studies of which we are aware have compared patterns of introgression between RAD and microsatellite markers: Bradbury *et al*. (2015) found little or no introgression with microsatellite markers in salmon, but evidence for introgression with RAD SNPs. On the other hand, Hohenlohe *et al*. (2013) found slightly lower estimates for introgression from RAD SNPs than from microsatellites in trout. Candy *et al*. (2015) found a close correspondence between RAD and microsatellite (Beacham *et al*. 2005) assessments of population differentiation in a Pacific smelt, with the RAD data yielding higher resolution. To our knowledge, only one other study has analysed introgression between a diploid and a tetraploid with RAD variants (Clark *et al*. 2015) and this showed introgression mainly from the diploid to the tetraploid, but rare diploids had some introgression from the tetraploid.

In our previous study using microsatellite markers (Wang *et al*. 2014b), we concluded that the cline of introgression from *B. nana* deep into the range of *B. pubescens* was most likely due to past range retreat of *B. nana* accompanied by hybridization with expanding populations of *B. pubescens*. This explains why the trail of introgression from the small *B. nana* shrubs penetrates deep into the distribution of *B. pubescens*, far to the south of the current range of *B. nana* (see Introduction). The RAD data presented here corroborate this by showing the pattern in a much larger sample of the genome. The RAD data set now opens up the potential for further studies to identify genes and genomic regions that have introgressed among the species, and ask whether these have adaptive potential. In future, we hope to investigate the genetic architecture and landscape of such regions, though as yet our *B. nana* reference genome (Wang *et al*. 2013) is too fragmented. We are currently using PacBio (Pacific Biosciences, Menlo Park, CA, USA) data to make this possible.

R.J.A.B. and J.S.B. collected samples; J.Z., I.K. and R.A.N. wrote R scripts; J.Z. and R.A.N. analysed data; J.Z., I.K., N.W., J.S.B., A.J. and R.J.A.B. performed research; and J.Z. and R.J.A.B. wrote the study.

## Data accessibility

- DNA read sequences for RAD loci: European Nucleotide Archive study accession ERP001869 http://www.ebi.ac.uk/ena/data/view/ERP001869 (sample accessions SAMEA3920742 to SAMEA3920954, submission accession ERA600270)
- r scripts used in the present analyses (also to create figures), *Betula* RAD reference and input files for population structure analyses: Dryad Digital Repository http://dx.doi.org/10.5061/dryad.815rj
- Herbarium sheets of specimens: British Museum London.

## Supporting information


**Table S1** Detailed information about and results of samples used in this study.
**Table S2** Parameter settings and version numbers for the CLC tools used in the present analyses.
**Table S3** Change in number of SNVs with different coverage thresholds being applied to the data set during the genotyping.
**Fig. S1** Flow chart outlining the analysis pipeline and filtering steps of the read mapping and variant calling.
**Fig. S2** Principal component analysis of 200 *Betula* samples at 49 025 biallelic variant loci.
**Fig. S3** Distribution of raw read frequencies at heterozygous sites covered by at least 30 reads.
**Fig. S4** Estimated genetic admixture of 200 *Betula* samples at 51 237 variant loci with *K *= 1 to 5.
**Fig. S5** Pairwise F_ST_ between each species pair at 49 025 biallelic variant loci.
**Fig. S6** Estimated genetic admixture of 177 *Betula* samples for which both microsatellite (upper panel) and RAD data (lower panel) was available.
