# Supplementary material for: Unidirectional diploid–tetraploid introgression among British birch trees with shifting ranges shown by restriction site‐associated markers
Source: Mol Ecol. 2016 May 11;25(11):2413–26. doi: 10.1111/mec.13644 (PMC4999052; doi:10.1111/mec.13644)
Supplement: Supplementary file 1 — Table S1 Detailed information about and results of samples used in this study. Table S2 Parameter settings and version numbers for the CLC tools used in the present analyses. Table S3 Change in number of SNVs with different coverage thresholds being applied to the data set during the genotyping. Fig. S1 Flow chart outlining the analysis pipeline and filtering steps of the read mapping and variant calling. Fig. S2 Principal component analysis of 200 Betula samples at 49 025 biallelic variant loci. Fig. S3 Distribution of raw read frequencies at heterozygous sites covered by at least 30 reads. Fig. S4 Estimated genetic admixture of 200 Betula samples at 51 237 variant loci with K = 1 to 5. Fig. S5 Pairwise FST between each species pair at 49 025 biallelic variant loci. Fig. S6 Estimated genetic admixture of 177 Betula samples for which both microsatellite (upper panel) and RAD data (lower panel) was available. [file MEC-25-2413-s001.docx]

**Online Supplementary Information**

**Unidirectional diploid-tetraploid introgression among British birch trees with shifting ranges shown by RAD markers**

Jasmin Zohren, Nian Wang, Igor Kardailsky, James S. Borrell, Anika Joecker, Richard A. Nichols, and Richard J. A. Buggs

**Table S1.** Detailed information about and results of samples used in this study. ‘struct_nana’ etc. refers to the estimated genetic admixture results from running STRUCTURE on 200 *Betula* samples at 51,237 variant loci. ‘AIC_dip’ etc. are the results of the beta-binomial model comparisons (smallest values in bold). ‘rep’ = replicate, ‘< 1m’ = less than one million reads, ‘> 50%’ = greater than 50% NA. Samples are sorted by organism and latitude.

**Table S2.** Parameter settings and version numbers for the CLC tools used in the present analyses. Details are given in the main text.

**Table S3.** Change in number of SNVs with different coverage thresholds being applied to the data set during the genotyping.

**Figure S1.** Flow chart outlining the analysis pipeline and filtering steps of the read mapping and variant calling. This part of the analysis was conducted in the CLC Genomics Workbench and the CLC Biomedical Genomics Workbench. ‘n’ = number of samples, ‘v’ = number of variants, ‘+3’ etc. indicates number of technical replicates.

**Figure S2.** Principal component analysis of 200 *Betula* samples at 49,025 biallelic variant loci. Individuals with > 10% missing values are highlighted. Symbols used correspond to Figure 3 in the main text. Figure created in R.

**Figure S3** *(separate PDF document).* Distribution of raw read frequencies at heterozygous sites covered by at least 30 reads. X-axes represent the frequency of an allele within an individual. Categories below 0.1 and above 0.9 were removed to avoid inclusion of sequencing errors. Plots are shown for each individual separately. Figure created in R.

**Figure S4.** Estimated genetic admixture of 200 *Betula* samples at 51,237 variant loci with *K* = 1 to 5. STRUCTURE was run with 50,000 repeats and a 10,000 burn-in period, repeated three times for each value of *K*. A) Admixture plots of all individuals at each *K*. Colours used correspond to Figure 4 in the main text. Figure created with ‘distruct1.1’. B) The log-likelihood values of each *K*. Figure created with STRUCTURE HARVESTER.

**Figure S5.** Pairwise F_ST_ between each species pair at 49,025 biallelic variant loci. The different species were treated as populations. Values of the boxes are (25% quartile, median, 75% quartile): 0.02, 0.06, and 0.17; 0.01, 0.02, and 0.06; 0.01, 0.02, and 0.05. Mean values are shown as grey dots: 0.17, 0.07, and 0.05. Figure created in R.

**Figure S6.** Estimated genetic admixture of 177 *Betula* samples for which both microsatellite (upper panel) and RAD data (lower panel) was available. Same individuals are aligned. Colours used correspond to Figure 4 in the main text. Figure created with ‘distruct1.1’.

**Table S1.** Detailed information about and results of samples used in this study. ‘struct_nana’ etc. refers to the estimated genetic admixture results from running STRUCTURE on 200 *Betula* samples at 51,237 variant loci. ‘AIC_dip’ etc. are the results of the beta-binomial model comparisons (smallest values in bold). ‘rep’ = replicate, ‘< 1m’ = less than one million reads, ‘> 50%’ = greater than 50% NA. Samples are sorted by organism and latitude.

| ID | Organism | Location | Latitude | Longitude | Altitude | Raw reads (million) | % mapped | struct_nana | struct_pend | struct_pub | AIC_dip (thousand) | AIC_trip (thousand) | AIC_tetra (thousand) | Comment |
| --- | --- | --- | --- | --- | --- | --- | --- | --- | --- | --- | --- | --- | --- | --- |
| 1090 | *B. nana* | Ben Loyal | 58.42 | -4.42 | 248.3 | 5.6 | 33.05 | 1 | 0 | 0 | **14.7** | 14.8 | 14.8 |  |
| 582 | *B. nana* | Ben Loyal | 58.42 | -4.42 | 274 | 5.2 | 62.19 | 1 | 0 | 0 | **49.8** | 51.8 | 51.7 | rep |
| 582 | *B. nana* | Ben Loyal | 58.42 | -4.42 | 274 | 6.9 | 63.67 | 1 | 0 | 0 | **59.7** | 62.6 | 62.2 | rep |
| 898 | *B. nana* | Ben Loyal | 58.42 | -4.42 | 254.9 | 5.3 | 63.73 | 1 | 0 | 0 | **41.0** | 42.3 | 42.1 |  |
| 714 | *B. nana* | Ben Loyal | 58.42 | -4.42 | 267.2 | 2.1 | 83.15 | 1 | 0 | 0 | **31.2** | 32.1 | 32.0 |  |
| 1029 | *B. nana* | Ben Loyal | 58.41 | -4.41 | 319.7 | 4.1 | 80.97 | 1 | 0 | 0 | **43.4** | 44.8 | 44.6 |  |
| 226 | *B. nana* | Ben Loyal | 58.41 | -4.41 | 316 | 4.7 | 63.38 | 1 | 0 | 0 | **38.6** | 39.5 | 39.5 |  |
| 230 | *B. nana* | Ben Loyal | 58.41 | -4.40 | 314.7 | 7.8 | 52.16 | 1 | 0 | 0 | **46.6** | 47.8 | 47.6 |  |
| 309 | *B. nana* | Ben Wyvis | 57.69 | -4.63 | 375.1 | 5.0 | 55.22 | 0.9978 | 0.002 | 0.0002 | **37.0** | 37.9 | 37.9 |  |
| 302 R A | *B. nana* | Ben Wyvis | 57.69 | -4.63 | 375.4 | 7.5 | 53.15 | 1 | 0 | 0 | **58.8** | 60.5 | 60.4 |  |
| 297 | *B. nana* | Ben Wyvis | 57.69 | -4.63 | 372.6 | 2.7 | 78.73 | 1 | 0 | 0 | **29.9** | 30.8 | 30.7 |  |
| 9710 | *B. nana* | Dundreggan | 57.23 | -4.75 | 449.4 | 8.4 | 81.74 | 1 | 0 | 0 | **80.7** | 83.6 | 82.7 |  |
| JBSPECIAL_3 | *B. nana* | Dundreggan Forest (e) | 57.23 | -4.74 | 436.9 | 0.1 | 59.20 | NA | NA | NA | NA | NA | NA | < 1m |
| JB33 | *B. nana* | Dundreggan Forest (e) | 57.23 | -4.74 | 439.2 | 7.5 | 77.95 | 1 | 0 | 0 | **66.1** | 69.1 | 68.5 |  |
| JB36 | *B. nana* | Dundreggan Forest (e) | 57.23 | -4.74 | 446.6 | 2.8 | 76.56 | 0.993 | 0.007 | 0 | **30.4** | 31.0 | 31.0 |  |
| JB34 | *B. nana* | Dundreggan Forest (e) | 57.23 | -4.74 | 444.4 | 13.9 | 78.95 | 1 | 0 | 0 | **64.1** | 66.8 | 66.0 |  |
| JB39 | *B. nana* | Dundreggan Forest (e) | 57.23 | -4.74 | 454.1 | 2.6 | 63.92 | 1 | 0 | 0 | **21.4** | 21.6 | 21.6 |  |
| JB42 | *B. nana* | Dundreggan Forest (e) | 57.23 | -4.74 | 456.6 | 3.7 | 78.52 | 1 | 0 | 0 | **49.9** | 51.5 | 51.4 |  |
| JB19 | *B. nana* | Dundreggan Forest (w) | 57.23 | -4.82 | 575.6 | 9.2 | 64.14 | 1 | 0 | 0 | **65.8** | 68.4 | 67.9 |  |
| JB24 | *B. nana* | Dundreggan Forest (w) | 57.22 | -4.82 | 579.8 | 11.1 | 61.23 | 1 | 0 | 0 | **65.9** | 68.3 | 67.6 |  |
| JB31 | *B. nana* | Dundreggan Forest (w) | 57.22 | -4.82 | 566.3 | 7.4 | 67.74 | 1 | 0 | 0 | **63.8** | 66.8 | 66.5 |  |
| JB27 | *B. nana* | Dundreggan Forest (w) | 57.22 | -4.82 | 566.5 | 1.8 | 76.62 | 1 | 0 | 0 | **14.5** | 14.6 | 14.6 |  |
| JB29 | *B. nana* | Dundreggan Forest (w) | 57.22 | -4.82 | 562.5 | 6.7 | 60.47 | 1 | 0 | 0 | **58.6** | 60.7 | 60.6 |  |
| JB15 | *B. nana* | Glenmore Forest | 56.99 | -3.79 | 696.2 | 16.4 | 74.32 | 1 | 0 | 0 | **56.0** | 58.4 | 57.7 |  |
| JB4 | *B. nana* | Glenmore Forest | 56.99 | -3.79 | 686.8 | 12.1 | 82.88 | 1 | 0 | 0 | **66.6** | 70.1 | 69.0 |  |
| JB7 | *B. nana* | Glenmore Forest | 56.99 | -3.80 | 674 | 10.1 | 75.49 | 1 | 0 | 0 | **73.0** | 76.6 | 75.7 |  |
| JB8 | *B. nana* | Glenmore Forest | 56.99 | -3.80 | 672.6 | 18.8 | 69.12 | 1 | 0 | 0 | **63.1** | 65.2 | 64.3 |  |
| JB10 | *B. nana* | Glenmore Forest | 56.99 | -3.80 | 676 | 2.5 | 70.06 | 1 | 0 | 0 | **25.8** | 26.3 | 26.3 |  |
| 1214 | *B. nana* | Loch Muick | 56.92 | -3.20 | 546.7 | 4.9 | 73.27 | 1 | 0 | 0 | **50.3** | 52.0 | 51.7 |  |
| 1224 | *B. nana* | Loch Muick | 56.92 | -3.20 | 589.5 | 4.1 | 79.32 | 1 | 0 | 0 | **48.8** | 50.5 | 50.3 |  |
| 1260 | *B. nana* | Loch Muick | 56.92 | -3.20 | 643 | 26.6 | 82.66 | NA | NA | NA | NA | NA | NA | > 50% |
| 1247 | *B. nana* | Loch Muick | 56.92 | -3.20 | 680.9 | 11.9 | 79.15 | 1 | 0 | 0 | **67.3** | 72.0 | 70.6 |  |
| 439 | *B. nana* | Ben Gulabin | 56.84 | -3.47 | 601 | 7.9 | 72.89 | 1 | 0 | 0 | **61.1** | 64.2 | 63.5 |  |
| 437 | *B. nana* | Ben Gulabin | 56.84 | -3.47 | 600 | 7.0 | 82.10 | 1 | 0 | 0 | **61.8** | 65.4 | 64.5 |  |
| 441 | *B. nana* | Ben Gulabin | 56.84 | -3.47 | 597.7 | 8.2 | 81.28 | 1 | 0 | 0 | **66.0** | 69.9 | 68.9 |  |
| 501 | *B. nana* | Rannoch Moor | 56.63 | -4.74 | 297.6 | 8.0 | 76.94 | 1 | 0 | 0 | **67.3** | 70.8 | 69.9 |  |
| 502 | *B. nana* | Rannoch Moor | 56.63 | -4.74 | 305.1 | 6.3 | 74.11 | 1 | 0 | 0 | **61.8** | 64.9 | 64.3 |  |
| 481 | *B. nana* | Rannoch Moor | 56.63 | -4.75 | 301.2 | 5.8 | 48.69 | 1 | 0 | 0 | **41.4** | 42.5 | 42.5 |  |
| 198i | *B. pendula* | Urqhart Castle | 57.32 | -4.45 | 89.3 | 1.6 | 82.18 | 0 | 1 | 0 | **16.7** | 17.0 | 16.9 |  |
| 198n | *B. pendula* | Urqhart Castle | 57.32 | -4.45 | 89.3 | 0.0 | 76.86 | NA | NA | NA | NA | NA | NA | < 1m |
| 1147 | *B. pendula* | Aviemore (s) | 57.17 | -3.83 | 206.1 | 8.8 | 83.97 | 0 | 1 | 0 | **48.8** | 50.3 | 49.8 |  |
| 1148 | *B. pendula* | Aviemore (s) | 57.16 | -3.84 | 223 | 6.1 | 79.51 | 0 | 1 | 0 | **49.4** | 51.1 | 50.9 |  |
| 1151 | *B. pendula* | South of Aviemore | 57.12 | -3.90 | 240.5 | 0.0 | 85.15 | NA | NA | NA | NA | NA | NA | < 1m |
| 461-2 | *B. pendula* | Rinabaich | 57.05 | -3.15 | 266.7 | 3.8 | 83.82 | 0 | 1 | 0 | **45.2** | 46.0 | 46.0 |  |
| 461c | *B. pendula* | Rinabaich | 57.05 | -3.15 | 266.7 | 3.8 | 81.28 | 0 | 1 | 0 | **31.7** | 32.5 | 32.2 |  |
| 461e | *B. pendula* | Rinabaich | 57.05 | -3.15 | 266.7 | 6.7 | 84.71 | 0 | 1 | 0 | **41.1** | 42.4 | 42.1 |  |
| 574 | *B. pendula* | Glen Lui, nr Braemar | 57.01 | -3.55 | 420.7 | 6.6 | 85.35 | 0 | 1 | 0 | **63.6** | 67.4 | 66.8 |  |
| 462w | *B. pendula* | Glen Muick | 57.00 | -3.08 | 301.3 | 2.4 | 83.37 | 0 | 1 | 0 | **31.2** | 32.3 | 32.1 |  |
| 530x-6 | *B. pendula* | Perth | 56.57 | -3.32 | 64.8 | 8.3 | 78.16 | 0 | 1 | 0 | **63.5** | 65.2 | 65.2 |  |
| 530xi-2 | *B. pendula* | Perth | 56.57 | -3.32 | 64.8 | 7.1 | 80.89 | 0 | 1 | 0 | **64.7** | 65.7 | 65.6 |  |
| 2457h | *B. pendula* | ConsettWood | 54.83 | -1.90 | 184.8 | 7.9 | 82.72 | 0 | 1 | 0 | **52.9** | 54.0 | 53.7 |  |
| 2457i | *B. pendula* | ConsettWood | 54.83 | -1.90 | 184.8 | 3.3 | 81.72 | 0 | 1 | 0 | **35.3** | 35.9 | 35.8 |  |
| 2457o | *B. pendula* | ConsettWood | 54.83 | -1.90 | 184.8 | 7.2 | 82.39 | 0 | 1 | 0 | **65.7** | 67.1 | 67.0 |  |
| 1163 | *B. pendula* | Flaxby, North Yorkshire | 54.01 | -1.39 | 33.7 | 5.3 | 83.47 | 0 | 1 | 0 | **51.7** | 54.1 | 53.6 |  |
| 2440 | *B. pendula* | Birchover, Derby | 53.16 | -1.62 | 241.4 | 5.7 | 78.55 | 0 | 1 | 0 | **62.0** | 63.3 | 63.4 |  |
| 2438 | *B. pendula* | Birchover, Derby | 53.16 | -1.62 | 228.4 | 11.0 | 80.36 | 0 | 1 | 0 | **54.2** | 55.7 | 55.4 |  |
| 2439 | *B. pendula* | Birchover, Derby | 53.16 | -1.63 | 235.5 | 7.4 | 83.21 | 0 | 1 | 0 | **60.8** | 62.4 | 62.1 |  |
| 8P_001R | *B. pendula* | Sheringham Wood | 52.93 | 1.20 | 73.2 | 5.2 | 83.64 | 0 | 0.936 | 0.064 | **59.2** | 62.1 | 61.7 |  |
| 8P_006R | *B. pendula* | Sheringham Wood | 52.93 | 1.20 | 73.2 | 5.5 | 84.42 | 0 | 1 | 0 | **61.3** | 63.8 | 63.2 |  |
| 8P_010R | *B. pendula* | Sheringham Wood | 52.93 | 1.20 | 73.2 | 4.2 | 83.61 | 0 | 1 | 0 | **61.6** | 63.8 | 63.4 |  |
| 2417e | *B. pendula* | GulletWood, Worcs | 52.04 | -2.35 | 142.9 | 10.5 | 81.75 | 0 | 1 | 0 | **55.8** | 57.2 | 56.9 |  |
| 2420a | *B. pendula* | GulletWood, Worcs | 52.04 | -2.35 | 127.3 | 11.9 | 57.75 | 0 | 1 | 0 | **47.4** | 47.8 | 47.6 |  |
| 2420b | *B. pendula* | GulletWood, Worcs | 52.04 | -2.35 | 127.3 | 6.8 | 80.26 | 0 | 1 | 0 | **39.7** | 40.1 | 39.9 |  |
| 2350 | *B. pendula* | BreconBeacons3 | 51.92 | -3.17 | 317.9 | 6.6 | 77.05 | 0 | 1 | 0 | **46.7** | 47.3 | 47.2 |  |
| 2347 | *B. pendula* | BreconBeacons3 | 51.92 | -3.17 | 299.3 | 4.8 | 79.19 | 0 | 1 | 0 | 25.7 | 25.8 | **25.7** |  |
| 2346 | *B. pendula* | BreconBeacons3 | 51.92 | -3.17 | 297.2 | 4.1 | 81.01 | 0 | 1 | 0 | **32.1** | 32.3 | 32.2 |  |
| 2310 | *B. pendula* | BreconBeacons1 | 51.82 | -3.05 | 185.2 | 5.1 | 77.81 | 0 | 1 | 0 | **32.7** | 33.1 | 32.8 |  |
| 2315 | *B. pendula* | BreconBeacons1 | 51.82 | -3.05 | 176.5 | 9.0 | 82.97 | 0 | 1 | 0 | **53.7** | 55.2 | 54.8 |  |
| 2320 | *B. pendula* | BreconBeacons1 | 51.82 | -3.05 | 181.8 | 3.4 | 62.77 | NA | NA | NA | NA | NA | NA | > 50% |
| 14_007 | *B. pendula* | E of Brockenhurst | 50.82 | -1.53 | 37.1 | 4.0 | 84.81 | 0 | 1 | 0 | **54.4** | 57.0 | 56.8 |  |
| 14_008 | *B. pendula* | E of Brockenhurst | 50.82 | -1.53 | 37.1 | 4.3 | 81.69 | 0 | 1 | 0 | **58.0** | 60.4 | 60.2 |  |
| 14_009 | *B. pendula* | E of Brockenhurst | 50.82 | -1.53 | 37.1 | 5.2 | 84.59 | 0 | 1 | 0 | **61.2** | 64.7 | 64.4 |  |
| 2380 | *B. pendula* | DartmoorBog | 50.52 | -3.82 | 101.4 | 5.9 | 81.79 | 0 | 1 | 0 | **59.8** | 62.5 | 62.0 |  |
| 2361 | *B. pendula* | DartmoorHotel | 50.52 | -3.80 | 106.9 | 4.4 | 73.22 | 0 | 1 | 0 | **29.5** | 29.8 | 29.7 |  |
| 2354 | *B. pendula* | DartmoorHotel | 50.52 | -3.80 | 98.1 | 8.5 | 83.42 | 0 | 1 | 0 | 38.6 | 38.8 | **38.6** |  |
| 1183a | *B. pubescens* | Berriedale Wood, Orkney | 58.89 | -3.38 | 38 | 4.8 | 85.08 | 0.019 | 0.0127 | 0.9684 | 172.0 | 168.9 | **167.2** |  |
| 1183d | *B. pubescens* | Orkney | 58.89 | -3.38 | 38 | 1.2 | 80.18 | NA | NA | NA | NA | NA | NA | > 50% |
| 1183r | *B. pubescens* | Orkney | 58.89 | -3.38 | 38 | 4.2 | 85.26 | 0.0176 | 0.0203 | 0.9621 | 106.3 | 104.5 | **103.4** |  |
| 277g | *B. pubescens* | Betty Hill | 58.53 | -4.21 | 46 | 5.7 | 85.50 | 0.02 | 0.0195 | 0.9605 | 176.4 | 173.2 | **171.5** |  |
| 277n | *B. pubescens* | Betty Hill | 58.53 | -4.21 | 46 | 7.1 | 83.72 | 0.0253 | 0.0126 | 0.9621 | 178.2 | 175.1 | **173.3** |  |
| 278g | *B. pubescens* | Loch Linnhe | 58.49 | -4.66 | 23.7 | 4.0 | 84.24 | 0.026 | 0.0093 | 0.9647 | 145.7 | 143.2 | **141.8** |  |
| 074a | *B. pubescens* | The Crawford Population | 58.48 | -4.22 | 75 | 4.2 | 83.06 | 0.025 | 0.0178 | 0.9572 | 124.0 | 121.8 | **120.6** |  |
| 074d | *B. pubescens* | The Crawford Population | 58.48 | -4.22 | 75 | 8.3 | 85.44 | 0.025 | 0.0145 | 0.9606 | 196.4 | 192.8 | **190.8** |  |
| 1123 | *B. pubescens* | Castle Varich Woods | 58.48 | -4.44 | 68.2 | 12.3 | 84.17 | 0.0081 | 0.001 | 0.9909 | 179.8 | 176.7 | **175.2** | rep |
| 1123 | *B. pubescens* | Castle Varich Woods | 58.48 | -4.44 | 68.2 | 4.7 | 84.25 | 0.008 | 0.001 | 0.991 | 165.2 | 162.1 | **160.2** | rep |
| 1124 | *B. pubescens* | Tongue | 58.48 | -4.44 | 68.9 | 4.2 | 84.17 | 0.034 | 0.0064 | 0.9596 | 167.5 | 164.4 | **162.8** |  |
| 1120 | *B. pubescens* | Castle Varich Woods | 58.48 | -4.43 | 7 | 9.5 | 84.15 | 0.018 | 0.0172 | 0.9648 | 154.1 | 151.6 | **150.4** |  |
| 1119 | *B. pubescens* | Castle Varich Woods | 58.47 | -4.42 | 5.6 | 8.3 | 84.48 | 0.018 | 0.0201 | 0.9619 | 188.4 | 185.2 | **183.3** |  |
| 1118 | *B. pubescens* | Tongue | 58.47 | -4.42 | 17.5 | 8.1 | 84.39 | 0.033 | 0.0013 | 0.9657 | 182.7 | 179.6 | **178.0** |  |
| 1127 | *B. pubescens* | Tongue | 58.46 | -4.42 | 19.4 | 5.8 | 83.68 | 0.012 | 0.0134 | 0.9746 | 155.5 | 152.8 | **151.2** |  |
| 1126 | *B. pubescens* | Tongue | 58.46 | -4.42 | 20.9 | 6.5 | 83.51 | 0.0199 | 0.0165 | 0.9636 | 158.3 | 155.5 | **153.8** |  |
| 1547 | *B. pubescens* | Ben Loyal | 58.44 | -4.42 | 41.8 | 7.7 | 83.21 | 0.033 | 0.0125 | 0.9545 | 172.5 | 169.6 | **168.0** |  |
| 1553c | *B. pubescens* | Ben Loyal | 58.44 | -4.42 | 57.1 | 8.0 | 83.36 | 0.0246 | 0.0134 | 0.962 | 211.9 | 207.8 | **204.7** |  |
| 1554 | *B. pubescens* | Ben Loyal | 58.44 | -4.42 | 58 | 7.3 | 84.17 | 0.034 | 0.0187 | 0.9473 | 200.9 | 196.8 | **194.0** |  |
| 1560 | *B. pubescens* | Ben Loyal | 58.43 | -4.42 | 59.2 | 6.3 | 84.53 | 0.026 | 0.0054 | 0.9686 | 195.8 | 192.0 | **189.7** |  |
| 1564 | *B. pubescens* | Ben Loyal | 58.43 | -4.42 | 73.3 | 9.5 | 85.57 | 0.0353 | 0.0104 | 0.9543 | 209.1 | 205.1 | **202.2** |  |
| 1565 | *B. pubescens* | Ben Loyal | 58.43 | -4.43 | 78.1 | 10.4 | 85.00 | 0.0298 | 0.0139 | 0.9563 | 206.8 | 202.6 | **199.7** |  |
| 1578 | *B. pubescens* | Ben Loyal | 58.42 | -4.42 | 169.8 | 4.6 | 86.31 | 0.0219 | 0.0005 | 0.9776 | 174.9 | 171.6 | **169.9** | rep |
| 1578 | *B. pubescens* | Ben Loyal | 58.42 | -4.42 | 169.8 | 9.9 | 86.71 | 0.0264 | 0.0009 | 0.9726 | 206.6 | 202.4 | **199.5** | rep |
| 1579 | *B. pubescens* | Ben Loyal | 58.42 | -4.42 | 179.2 | 11.0 | 85.34 | 0.0288 | 0.0102 | 0.9609 | 186.2 | 182.8 | **180.9** |  |
| 1002 | *B. pubescens* | Ben Loyal | 58.42 | -4.42 | 195.5 | 5.1 | 85.55 | 0.033 | 0.0181 | 0.9489 | 172.8 | 169.6 | **167.4** |  |
| 801 | *B. pubescens* | Ben Loyal | 58.42 | -4.42 | 230.7 | 10.3 | 84.06 | 0.02 | 0.011 | 0.969 | 210.6 | 206.4 | **203.2** |  |
| 1009 | *B. pubescens* | Ben Loyal | 58.42 | -4.42 | 231.6 | 6.6 | 84.24 | 0.023 | 0.0152 | 0.9618 | 200.4 | 196.6 | **194.0** |  |
| 1011 | *B. pubescens* | Ben Loyal | 58.42 | -4.42 | 244.6 | 7.2 | 84.27 | 0.034 | 0.0121 | 0.9539 | 167.2 | 164.2 | **162.4** |  |
| 583 | *B. pubescens* | Ben Loyal | 58.42 | -4.42 | 277.7 | 12.2 | 69.84 | 0.0379 | 0.0143 | 0.9477 | 207.6 | 203.5 | **200.9** |  |
| 605 | *B. pubescens* | Ben Loyal | 58.42 | -4.42 | 279.9 | 4.2 | 83.42 | 0.0192 | 0.0185 | 0.9623 | 155.4 | 152.6 | **151.0** |  |
| 750 | *B. pubescens* | Ben Loyal | 58.42 | -4.42 | 259.3 | 6.6 | 83.96 | 0.0155 | 0.0161 | 0.9684 | 190.7 | 187.2 | **184.8** |  |
| 727 | *B. pubescens* | Ben Loyal | 58.42 | -4.42 | 262 | 6.1 | 84.20 | 0.019 | 0.0087 | 0.9724 | 182.2 | 178.9 | **176.7** |  |
| 1015 | *B. pubescens* | Ben Loyal | 58.42 | -4.42 | 259.5 | 6.0 | 84.09 | 0.03 | 0.0138 | 0.9562 | 181.5 | 178.0 | **175.6** |  |
| 1016 | *B. pubescens* | Ben Loyal | 58.42 | -4.42 | 268.8 | 4.5 | 83.21 | 0.015 | 0.0022 | 0.9828 | 166.2 | 163.1 | **161.2** |  |
| 1017 | *B. pubescens* | Ben Loyal | 58.42 | -4.42 | 272.2 | 5.2 | 83.83 | 0.016 | 0.0105 | 0.9735 | 131.0 | 128.8 | **127.5** |  |
| 1045 | *B. pubescens* | Ben Loyal | 58.42 | -4.42 | 317.4 | 3.0 | 84.24 | 0.0069 | 0 | 0.9931 | 120.1 | 117.9 | **116.7** | rep |
| 1045 | *B. pubescens* | Ben Loyal | 58.42 | -4.42 | 317.4 | 5.9 | 84.41 | 0.0049 | 0 | 0.9951 | 185.8 | 182.1 | **179.9** | rep |
| 1131 | *B. pubescens* | Lairg | 58.04 | -4.45 | 105.4 | 4.4 | 81.54 | 0.021 | 0.001 | 0.978 | 102.0 | 100.2 | **99.1** |  |
| 579 | *B. pubescens* | Lairg | 58.03 | -4.42 | 93.7 | 4.9 | 83.37 | 0.02 | 0.0047 | 0.9753 | 127.7 | 125.4 | **124.0** |  |
| 1133 | *B. pubescens* | Lairg | 58.03 | -4.44 | 119.6 | 3.4 | 78.31 | 0.0185 | 0.0073 | 0.9742 | 65.9 | 64.8 | **64.0** |  |
| 283a | *B. pubescens* | Drumrunie | 57.99 | -5.11 | 118.8 | 3.8 | 83.16 | 0.018 | 0 | 0.982 | 101.8 | 100.0 | **99.0** |  |
| 283r | *B. pubescens* | Drumrunie | 57.99 | -5.11 | 118.8 | 7.0 | 56.51 | 0.0238 | 0.015 | 0.9612 | 92.2 | 90.7 | **89.8** |  |
| 1135 | *B. pubescens* | Ardgay | 57.99 | -5.11 | 23.3 | 4.4 | 79.45 | 0.021 | 0.0045 | 0.9745 | 149.7 | 147.1 | **145.6** |  |
| 1136 | *B. pubescens* | Ardgay | 57.88 | -4.36 | 31.6 | 5.7 | 83.88 | 0.0226 | 0.0037 | 0.9736 | 93.6 | 91.9 | **90.8** |  |
| 1134 | *B. pubescens* | Ardgay | 57.88 | -4.36 | 21.9 | 6.5 | 78.37 | 0.0175 | 0.0048 | 0.9778 | 144.5 | 141.9 | **140.3** |  |
| 285-1 | *B. pubescens* | Braemore | 57.88 | -4.36 | 104 | 5.7 | 85.62 | 0.016 | 0.0219 | 0.9621 | 129.0 | 126.8 | **125.5** |  |
| 325 | *B. pubescens* | Ben Wyvis | 57.76 | -5.03 | 383.6 | 8.2 | 80.13 | 0.001 | 0.0488 | 0.9502 | 176.9 | 174.4 | **173.2** | rep |
| 325 | *B. pubescens* | Ben Wyvis | 57.69 | -4.63 | 383.6 | 10.6 | 75.02 | 0.019 | 0 | 0.981 | 205.1 | 201.2 | **198.9** | rep |
| 1140 | *B. pubescens* | Cromarty | 57.68 | -4.00 | 111 | 5.8 | 79.01 | 0.0097 | 0.0069 | 0.9834 | 79.3 | 78.0 | **77.3** |  |
| 1138 | *B. pubescens* | Cromarty | 57.68 | -4.00 | 109.1 | 4.1 | 81.14 | 0.0218 | 0.0166 | 0.9616 | 100.7 | 98.9 | **97.8** |  |
| 1139 | *B. pubescens* | Cromarty | 57.68 | -4.01 | 107 | 3.8 | 81.35 | 0.011 | 0.0235 | 0.9655 | 87.0 | 85.6 | **84.8** |  |
| 198w | *B. pubescens* | Urqhart Castle | 57.32 | -4.45 | 89.3 | 2.2 | 84.16 | NA | NA | NA | NA | NA | NA | > 50% |
| 465c | *B. pubescens* | Lynemore | 57.28 | -3.55 | 384.6 | 6.3 | 84.59 | 0.015 | 0.0145 | 0.9705 | 122.8 | 120.8 | **119.5** |  |
| 465e | *B. pubescens* | Lynemore | 57.28 | -3.55 | 384.6 | 1.1 | 82.53 | NA | NA | NA | NA | NA | NA | > 50% |
| JBSPECIAL_2 | *B. pubescens* | Dundreggan Forest (e) | 57.23 | -4.74 | 448.2 | 14.9 | 81.98 | 0.0042 | 0.0186 | 0.9773 | 154.6 | 152.1 | **150.8** |  |
| 1150 | *B. pubescens* | Aviemore (s) | 57.14 | -3.86 | 212.5 | 6.6 | 85.02 | 0.025 | 0.0161 | 0.9589 | 179.8 | 176.3 | **174.1** |  |
| 1152 | *B. pubescens* | Aviemore (s) | 57.12 | -3.90 | 241.4 | 1.2 | 79.24 | NA | NA | NA | NA | NA | NA | > 50% |
| 1153 | *B. pubescens* | Aviemore (s) | 57.12 | -3.90 | 243.7 | 2.5 | 84.60 | 0.0239 | 0.0174 | 0.9587 | 94.0 | 92.3 | **91.4** | rep |
| 1153 | *B. pubescens* | Aviemore (s) | 57.12 | -3.90 | 243.7 | 0.0 | 29.83 | NA | NA | NA | NA | NA | NA | rep /  < 1m |
| 463i | *B. pubescens* | Gairnshiel Lodge (s-slope) | 57.10 | -3.15 | 374.8 | 9.0 | 83.83 | 0.013 | 0.0133 | 0.9737 | 208.0 | 204.0 | **201.4** |  |
| 1154 | *B. pubescens* | Kingussie, Highland | 57.08 | -3.98 | 251.9 | 7.7 | 83.32 | 0.0102 | 0.0069 | 0.9829 | 169.4 | 166.4 | **164.6** |  |
| 1155 | *B. pubescens* | Kingussie, Highland | 57.08 | -3.98 | 257.1 | 5.2 | 82.69 | 0.0142 | 0.0046 | 0.9812 | 77.1 | 75.9 | **75.4** |  |
| 1156 | *B. pubescens* | Kingussie, Highland | 57.08 | -3.98 | 254.6 | 13.7 | 83.94 | 0.025 | 0.0287 | 0.9463 | 144.9 | 142.6 | **141.6** |  |
| 567 | *B. pubescens* | Mar Estate ne Braemar | 57.02 | -3.57 | 423.4 | 10.4 | 85.28 | 0.0207 | 0.0136 | 0.9657 | 194.6 | 190.9 | **188.5** |  |
| 575 | *B. pubescens* | Mar Estate ne Braemar | 57.01 | -3.54 | 405.2 | 6.0 | 85.06 | 0.02 | 0.0122 | 0.9678 | 185.1 | 181.5 | **179.0** |  |
| 576 | *B. pubescens* | Mar Estate ne Braemar | 57.01 | -3.54 | 402.7 | 5.7 | 84.90 | 0.0181 | 0.0169 | 0.965 | 170.8 | 167.6 | **165.6** |  |
| 462i-1 | *B. pubescens* | Rinabaich/Glen Muick | 57.00 | -3.08 | 301.3 | 7.2 | 82.30 | 0.018 | 0.0046 | 0.9774 | 189.5 | 186.6 | **185.1** |  |
| 462n | *B. pubescens* | Glen Muick | 57.00 | -3.08 | 301.3 | 3.0 | 84.84 | 0.011 | 0.001 | 0.988 | 98.5 | 96.7 | **95.6** |  |
| 425f | *B. pubescens* | Loch Muick | 56.93 | -3.18 | 412.5 | 5.8 | 85.49 | 0.011 | 0 | 0.989 | 195.5 | 192.0 | **190.0** | rep |
| 425f | *B. pubescens* | Loch Muick | 56.93 | -3.18 | 412.5 | 7.7 | 84.85 | 0.011 | 0 | 0.989 | 200.6 | 196.7 | **194.0** | rep |
| 354 | *B. pubescens* | Loch Muick | 56.92 | -3.20 | 451.1 | 6.8 | 83.77 | 0.0152 | 0.0131 | 0.9718 | 176.1 | 172.7 | **170.3** |  |
| 364 | *B. pubescens* | Loch Muick | 56.92 | -3.20 | 509 | 8.7 | 81.83 | 0.03 | 0 | 0.97 | 195.2 | 191.5 | **189.1** |  |
| 381 | *B. pubescens* | Loch Muick | 56.92 | -3.20 | 565.1 | 13.4 | 59.96 | 0.031 | 0.018 | 0.951 | 209.1 | 204.8 | **201.6** |  |
| 1277 | *B. pubescens* | Loch Muick | 56.92 | -3.21 | 601.2 | 5.7 | 83.08 | 0.023 | 0.0181 | 0.9589 | 179.3 | 175.9 | **173.7** |  |
| 459c | *B. pubescens* | Ben Gulabin (nw) | 56.83 | -3.49 | 427.6 | 4.1 | 84.58 | 0.029 | 0.014 | 0.957 | 157.0 | 154.0 | **152.1** |  |
| 459i-4 | *B. pubescens* | Ben Gulabin (nw) | 56.83 | -3.49 | 427.6 | 10.4 | 79.78 | 0.0094 | 0.0065 | 0.9841 | 211.7 | 208.6 | **207.1** |  |
| 459w | *B. pubescens* | Ben Gulabin (nw) | 56.83 | -3.49 | 427.6 | 3.5 | 85.98 | 0.0279 | 0.0156 | 0.9565 | 140.7 | 138.0 | **136.5** |  |
| 467w | *B. pubescens* | Crianlarich | 56.42 | -4.51 | 177 | 1.6 | 85.37 | 0.0144 | 0.0026 | 0.983 | 54.0 | 53.0 | **52.2** |  |
| 466x-3 | *B. pubescens* | Bankhead Moss | 56.28 | -2.90 | 169.5 | 4.6 | 76.62 | 0.019 | 0.0039 | 0.9771 | 124.9 | 123.1 | **122.2** |  |
| 195a | *B. pubescens* | Loch Lomond | 56.23 | -4.70 | 33.8 | 7.4 | 68.74 | 0.0061 | 0.0238 | 0.9701 | 185.6 | 182.0 | **179.6** |  |
| 1159 | *B. pubescens* | Johnstonebridge, Dumfries | 55.23 | -3.43 | 143.7 | 9.4 | 76.87 | 0.008 | 0.0242 | 0.9678 | 190.0 | 186.4 | **184.1** |  |
| 1157 | *B. pubescens* | Johnstonebridge, Dumfries | 55.22 | -3.42 | 100 | 4.0 | 83.82 | 0.008 | 0.0156 | 0.9764 | 142.2 | 139.6 | **138.1** |  |
| 1158 | *B. pubescens* | Johnstonebridge, Dumfries | 55.22 | -3.42 | 97.5 | 3.6 | 84.24 | 0.004 | 0.0616 | 0.9345 | 138.7 | 136.3 | **134.9** | rep |
| 1158 | *B. pubescens* | Johnstonebridge, Dumfries | 55.22 | -3.42 | 97.5 | 2.3 | 57.56 | NA | NA | NA | NA | NA | NA | rep /  > 50% |
| 2459 | *B. pubescens* | RoseberryTopping | 54.51 | -1.11 | 204.5 | 6.5 | 84.26 | 0 | 0.0971 | 0.9029 | 140.4 | 138.1 | **136.8** |  |
| 2468 | *B. pubescens* | RoseberryTopping | 54.51 | -1.11 | 211 | 12.8 | 82.71 | 0 | 0.1034 | 0.8966 | 164.9 | 162.7 | **161.9** |  |
| 2470 | *B. pubescens* | RoseberryTopping | 54.51 | -1.11 | 216 | 8.8 | 84.71 | 0.0059 | 0.0445 | 0.9496 | 187.5 | 184.7 | **183.6** |  |
| 2473 | *B. pubescens* | RoseberryTopping | 54.51 | -1.11 | 245.3 | 12.8 | 83.72 | 0 | 0.0711 | 0.9289 | 143.5 | 141.5 | **140.6** |  |
| 2451b | *B. pubescens* | BirkPark, Yorks | 54.39 | -2.00 | 308.8 | 7.4 | 78.23 | 0.013 | 0.0018 | 0.9852 | 181.6 | 179.1 | **178.0** |  |
| 2451d | *B. pubescens* | BirkPark, Yorks | 54.39 | -2.00 | 308.8 | 10.3 | 82.29 | 0.0166 | 0.0115 | 0.9719 | 190.1 | 187.3 | **186.2** |  |
| 2451j | *B. pubescens* | BirkPark, Yorks | 54.39 | -2.00 | 308.8 | 9.7 | 81.15 | 0.009 | 0.011 | 0.98 | 184.8 | 181.7 | **180.0** |  |
| 2450a | *B. pubescens* | RichmondQuarry, Yorks | 54.39 | -1.83 | 153 | 8.3 | 76.41 | 0.0002 | 0.0073 | 0.9924 | 107.0 | 105.6 | **104.7** |  |
| 2450b | *B. pubescens* | RichmondQuarry, Yorks | 54.39 | -1.83 | 153 | 4.6 | 77.72 | 0.0151 | 0.0013 | 0.9836 | 97.8 | 96.2 | **95.4** |  |
| 2450c | *B. pubescens* | RichmondQuarry, Yorks | 54.39 | -1.83 | 153 | 6.4 | 80.29 | 0 | 0.0622 | 0.9378 | 155.5 | 152.9 | **151.5** |  |
| 1164 | *B. pubescens* | Flaxby, North Yorkshire | 54.01 | -1.39 | 37 | 2.3 | 81.03 | 0.001 | 0.0178 | 0.9812 | 59.6 | 58.6 | **57.8** |  |
| 1165 | *B. pubescens* | Flaxby, North Yorkshire | 54.01 | -1.39 | 36.9 | 5.5 | 85.20 | 0 | 0.032 | 0.968 | 178.4 | 174.9 | **172.7** |  |
| 2449b | *B. pubescens* | BoltonAbbey | 54.00 | -1.89 | 116.9 | 4.2 | 82.91 | 0 | 0.0449 | 0.9551 | 140.3 | 138.2 | **137.1** |  |
| 2449c | *B. pubescens* | BoltonAbbey | 54.00 | -1.89 | 116.9 | 11.7 | 85.68 | 0.0002 | 0.0461 | 0.9538 | 173.2 | 170.7 | **169.8** |  |
| 2449d | *B. pubescens* | BoltonAbbey | 54.00 | -1.89 | 116.9 | 9.9 | 82.92 | 0 | 0.0612 | 0.9388 | 195.0 | 192.2 | **190.9** |  |
| 2448h | *B. pubescens* | ScoutCamp, Lancs | 53.80 | -2.41 | 106.4 | 7.2 | 81.35 | 0 | 0.0564 | 0.9436 | 154.2 | 151.6 | **150.3** |  |
| 2448i | *B. pubescens* | ScoutCamp, Lancs | 53.80 | -2.41 | 106.4 | 8.2 | 81.47 | 0 | 0.0158 | 0.9843 | 182.8 | 179.8 | **178.3** |  |
| 2448j | *B. pubescens* | ScoutCamp, Lancs | 53.80 | -2.41 | 106.4 | 3.9 | 74.54 | 0 | 0.0218 | 0.9782 | 122.2 | 120.1 | **119.1** |  |
| 2446a | *B. pubescens* | GlossopWood, Derby | 53.43 | -1.95 | 205.2 | 6.1 | 81.97 | 0.0019 | 0.0279 | 0.9702 | 106.2 | 104.6 | **103.5** |  |
| 2446m | *B. pubescens* | GlossopWood, Derby | 53.43 | -1.95 | 205.2 | 7.1 | 84.39 | 0 | 0.0252 | 0.9748 | 137.3 | 135.1 | **133.8** |  |
| 2446s | *B. pubescens* | GlossopWood, Derby | 53.43 | -1.95 | 205.2 | 8.6 | 79.73 | 0.0062 | 0.0432 | 0.9506 | 194.2 | 190.6 | **188.5** |  |
| 7_004R | *B. pubescens* | Woodbastwick Marshes | 52.69 | 1.46 | 18.3 | 3.9 | 84.48 | 0.0049 | 0.0527 | 0.9424 | 143.2 | 140.5 | **139.1** |  |
| 7_008 | *B. pubescens* | Woodbastwick Marshes | 52.69 | 1.46 | 18.3 | 9.9 | 83.88 | 0 | 0.0551 | 0.9449 | 189.8 | 186.5 | **184.6** |  |
| 6_001R | *B. pubescens* | Woodbastwick Marshes | 52.68 | 1.46 | 10.5 | 7.2 | 85.48 | 0 | 0.0852 | 0.9148 | 185.1 | 181.7 | **179.8** |  |
| 5_012 | *B. pubescens* | S of Attleborough | 52.49 | 0.99 | 32.7 | 4.2 | 83.13 | 0 | 0.0555 | 0.9445 | 148.5 | 145.9 | **144.4** |  |
| 5_014 | *B. pubescens* | S of Attleborough | 52.49 | 0.99 | 32.7 | 6.5 | 84.43 | 0 | 0.1062 | 0.8938 | 178.5 | 175.3 | **173.3** |  |
| 1184a | *B. pubescens* | Eccles Car, Norfolk | 52.45 | 1.00 | 36 | 1.1 | 83.06 | NA | NA | NA | NA | NA | NA | > 50% |
| 1184b | *B. pubescens* | Eccles Car, Norfolk | 52.45 | 1.00 | 36 | 3.1 | 85.52 | 0 | 0.0529 | 0.9471 | 112.7 | 110.6 | **109.4** |  |
| 1184c | *B. pubescens* | Eccles Car, Norfolk | 52.45 | 1.00 | 36 | 4.5 | 84.54 | 0 | 0.075 | 0.925 | 160.9 | 158.0 | **156.4** |  |
| 2437b | *B. pubescens* | RytonWood, Warwick | 52.35 | -1.45 | 90.2 | 4.7 | 79.42 | 0 | 0.0444 | 0.9556 | 92.4 | 91.1 | **90.4** |  |
| 2434b | *B. pubescens* | RytonWood, Warwick | 52.35 | -1.45 | 97.4 | 5.0 | 80.70 | 0 | 0.0201 | 0.9799 | 129.4 | 127.2 | **125.9** |  |
| 2431a | *B. pubescens* | RytonWood, Warwick | 52.35 | -1.45 | 85.5 | 7.1 | 82.38 | 0 | 0.0601 | 0.9399 | 155.6 | 153.0 | **151.5** |  |
| 2322 | *B. pubescens* | BreconBeacons2 | 51.85 | -3.18 | 252 | 6.1 | 81.66 | 0.0011 | 0.0481 | 0.9508 | 118.3 | 116.7 | **115.9** |  |
| 2325 | *B. pubescens* | BreconBeacons2 | 51.85 | -3.18 | 256.6 | 4.2 | 76.31 | 0.001 | 0.0065 | 0.9925 | 67.5 | 66.4 | **65.9** |  |
| 2331 | *B. pubescens* | BreconBeacons2 | 51.85 | -3.18 | 268.2 | 5.1 | 84.16 | 0.0061 | 0.0321 | 0.9618 | 113.7 | 112.1 | **111.3** |  |
| 2_001R | *B. pubescens* | Danbury | 51.71 | 0.57 | 68.2 | 6.7 | 84.20 | 0 | 0.0763 | 0.9237 | 179.9 | 176.6 | **174.9** |  |
| 2_004R | *B. pubescens* | Danbury | 51.71 | 0.57 | 68.2 | 8.1 | 82.38 | 0 | 0.0729 | 0.9271 | 181.1 | 177.9 | **176.2** |  |
| 3_001R | *B. pubescens* | Danbury | 51.71 | 0.57 | 70.5 | 7.3 | 83.81 | 0 | 0.0931 | 0.9069 | 177.5 | 174.3 | **172.5** |  |
| 1172 | *B. pubescens* | Capel, Kent | 51.17 | 0.34 | 61.7 | 6.1 | 79.96 | 0 | 0.0841 | 0.9159 | 177.8 | 174.5 | **172.4** |  |
| 1177 | *B. pubescens* | Capel, Kent | 51.17 | 0.34 | 44.9 | 3.6 | 77.03 | 0 | 0.407 | 0.593 | 95.1 | 93.4 | **92.2** |  |
| 24 | *B. pubescens* | Long Copse | 51.11 | -0.93 | 123.6​ | 6.4 | 85.88 | 0 | 0.0801 | 0.92 | 183.5 | 180.2 | **178.4** |  |
| 38_005R | *B. pubescens* | Williand Wood | 51.04 | -0.61 | 49.7 | 4.5 | 82.67 | 0.001 | 0.0825 | 0.9165 | 148.4 | 145.7 | **144.1** |  |
| 40_009 | *B. pubescens* | Cootham | 50.92 | -0.48 | 37 | 8.0 | 79.13 | 0 | 0.0868 | 0.9132 | 189.5 | 185.9 | **183.5** |  |
| 40_012 | *B. pubescens* | Cootham | 50.92 | -0.48 | 37 | 4.2 | 80.07 | 0 | 0.1691 | 0.8309 | 144.4 | 141.8 | **140.4** |  |
| 13_004R | *B. pubescens* | N of Beaulieu | 50.85 | -1.45 | 17 | 8.3 | 83.71 | 0 | 0.0926 | 0.9073 | 188.5 | 184.9 | **182.8** |  |
| 13_006R | *B. pubescens* | N of Beaulieu | 50.85 | -1.45 | 17 | 6.5 | 82.52 | 0 | 0.0901 | 0.9099 | 171.9 | 168.9 | **167.3** |  |
| 2377 | *B. pubescens* | DartmoorBog | 50.52 | -3.82 | 100.9 | 0.0 | 82.81 | NA | NA | NA | NA | NA | NA | < 1m |
| 2376 | *B. pubescens* | DartmoorBog | 50.52 | -3.82 | 88.4 | 6.1 | 82.68 | 0 | 0.0439 | 0.9561 | 129.8 | 127.7 | **126.6** |  |
| 2364a | *B. pubescens* | DartmoorHotel | 50.52 | -3.81 | 114.7 | 3.8 | 82.30 | 0.005 | 0.0393 | 0.9557 | 90.1 | 88.8 | **88.1** |  |
| 2407 | *B. pubescens* | DartmoorBurrator | 50.49 | -4.04 | 216.2 | 9.7 | 79.42 | 0 | 0.0501 | 0.9499 | 154.7 | 152.5 | **151.5** |  |
| 2389 | *B. pubescens* | DartmoorBurrator | 50.49 | -4.05 | 216.3 | 7.3 | 63.06 | 0 | 0.0824 | 0.9176 | 83.7 | 82.6 | **81.9** |  |
| 2384 | *B. pubescens* | DartmoorBurrator | 50.49 | -4.05 | 212.6 | 5.9 | 79.56 | 0.0001 | 0.0581 | 0.9418 | 90.4 | 89.2 | **88.7** |  |
| 1173 | Hybrid | Capel, Kent | 51.17 | 0.34 | 58.4 | 3.5 | 85.47 | 0 | 0.0439 | 0.9561 | 110.5 | **108.8** | 110.4 |  |

**Table S2.** Parameter settings and version numbers for the CLC tools used in the present analyses. Details are given in the main text.

| **‘Map Reads to Reference‘** | |
| --- | --- |
| Version: | CLC Genomics Grid Worker 6.5.2 |
| Modified by: | jzohren |
| References: | RADrefSeq_conc_annot |
| Masking mode: | No masking |
| Mismatch cost: | 2 |
| Insertion cost: | 3 |
| Deletion cost: | 3 |
| Length fraction: | 0.5 |
| Similarity fraction: | 0.8 |
| Global alignment: | No |
| Non-specific match handling: | Ignore |
| Output mode: | Create reads track |
| Create report: | No |
| Collect un-mapped reads: | No |
| Comments: | Reads mapped: 6,856,956 of 8,389,115 |
|  |  |
| **‘Local Realignment’** | |
| Version: | CLC Genomics Grid Worker 6.5.2 |
| Modified by: | jzohren |
| Realign unaligned ends: | Yes |
| Multi-pass realignment: | 2 |
| Guidance-variant track: | Not set |
| Output mode: | Create reads track |
| Output track of realigned regions: | No |
|  |  |
| **‘Low Frequency Variant Detection’** | |
| Version: | CLC Genomics Grid Worker 6.5.2 |
| Modified by: | jzohren |
| Required significance (%): | 1 |
| Ignore positions with coverage above: | 100,000 |
| Restrict calling to target regions: | Not set |
| Ignore broken pairs: | No |
| Ignore non-specific matches: | Reads |
| Minimum coverage: | 10 |
| Minimum count: | 2 |
| Minimum frequency (%): | 1 |
| Base quality filter: | No |
| Read direction filter: | No |
| Read position filter: | No |
| Relative read direction filter: | Yes |
| Significance (%): | 1 |
| Remove pyro-error variants: | No |
| Create track: | Yes |
| Create annotated table: | No |
| Create report: | No |
| Comments: | Found 3,909,255 variants |
|  |  |
| **‘Identify Known Mutations from Sample Mappings’** | |
| Version: | CLC Genomics Grid Worker 7.0 Beta 2 |
| Modified by: | jzohren |
| Variant track: | RADrefSeq_conc_annot_lr |
| Minimum coverage: | 10 |
| Detection frequency: | 20 |
| Create individual tracks: | Yes |
| Create overview track: | Yes |
| Ignore broken pairs: | No |
| Ignore non-specific matches: | No |

**Table S3.** Change in number of SNVs with different coverage thresholds being applied to the data set during the genotyping.

| Thresholds | No of  individuals | No of SNVs present in  at least one individual | No of SNVs present  in 80% of individuals (biallelic loci) | No of SNVs present  in all individuals (biallelic loci) | No of individuals  with > 50% NAs |
| --- | --- | --- | --- | --- | --- |
| 10, ∞ | 208 | 648,631 | 74,559 | 4,633 | 5 |
| 10, ∞^+^ | 203 | 645,317 | 77,787 | 9,661 | 1 |
| 20, ∞ | 208 | 373,958 | 52,945 | 1,218 | 9 |
| 10, 200 | 208 | 649,586 | 58,390 | 6 | 7 |
| 20, 200 | 208 | 373,838 | 35,472 | 0 | 11 |
| 20, 400 | 208 | 373,933 | 51,379 | 93 | 9 |
| 20, 400^+^ | 203 | 373,167 | 54,851 | 211 | 4 |
| 20, 400^+^ | 199 | 371,556 | 57,141 | 595 | 1 |
| 10/20, 200 | 208 | 546,015 | 44,127 (42,018) | 0 | 8 |
| 10/20, 200^+#^ | 200 | 541,080 | 51,237 (49,025) | 59 (57) | 0 |

^+^Same filters applied to already filtered data set after removing individuals with > 50% NAs.

^#^The filters for the final data set that was used in the present analyses.

**Figure S1.** Flow chart outlining the analysis pipeline and filtering steps of the read mapping and variant calling. This part of the analysis was conducted in the CLC Genomics Workbench and the CLC Biomedical Genomics Workbench. ‘n’ = number of samples, ‘v’ = number of variants, ‘+3’ etc. indicates number of technical replicates.

**Figure S2.** Principal component analysis of 200 *Betula* samples at 49,025 biallelic variant loci. Individuals with > 10% missing values are highlighted. Symbols used correspond to Figure 3 in the main text. Figure created in R.

**Figure S4.** Estimated genetic admixture of 200 *Betula* samples at 51,237 variant loci with *K* = 1 to 5. STRUCTURE was run with 50,000 repeats and a 10,000 burn-in period, repeated three times for each value of *K*. A) Admixture plots of all individuals at each *K*. Colours used correspond to Figure 4 in the main text. Figure created with ‘distruct1.1’. B) The log-likelihood values of each *K*. Figure created with STRUCTURE HARVESTER.


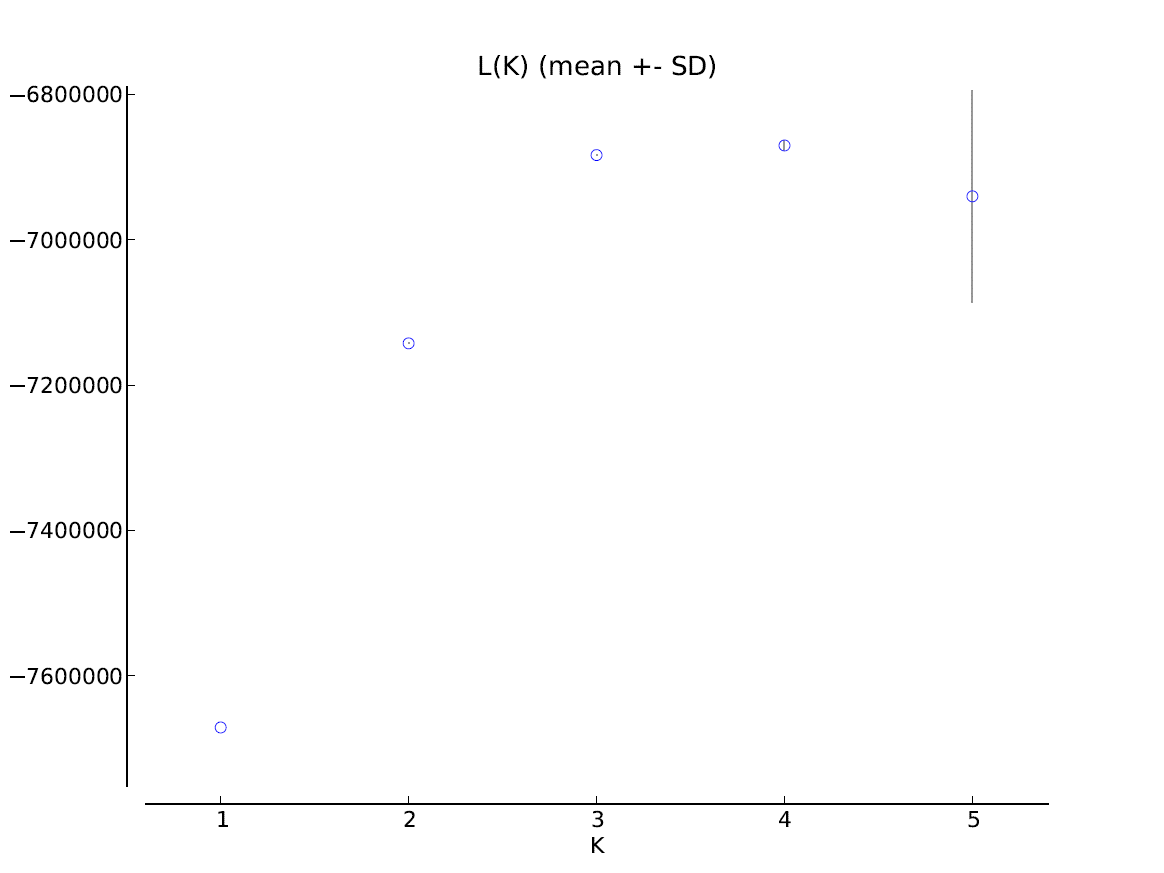


K = 3

K = 4

K = 5

K = 2

K = 1

*B. nana*

*B. pubescens*

*B. pendula*

**(A)**

**(B)**


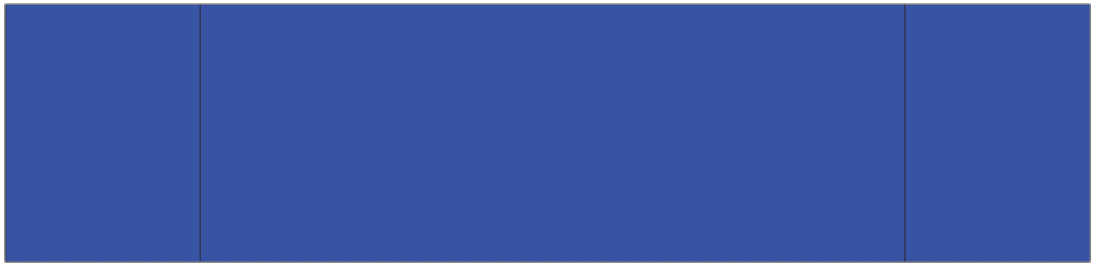

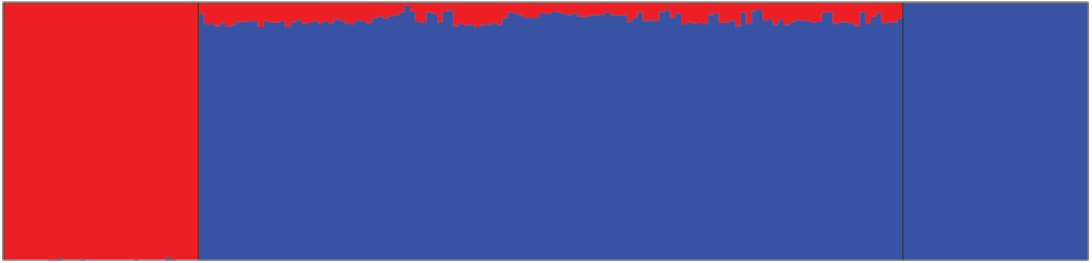

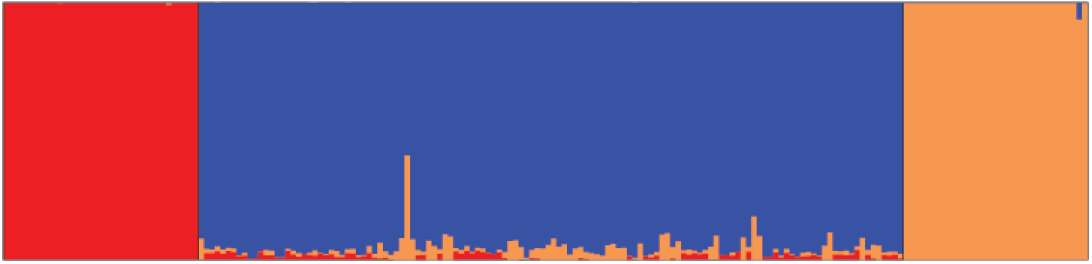

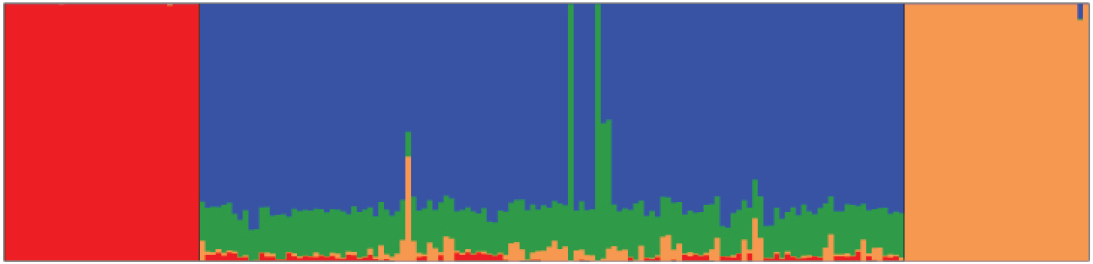

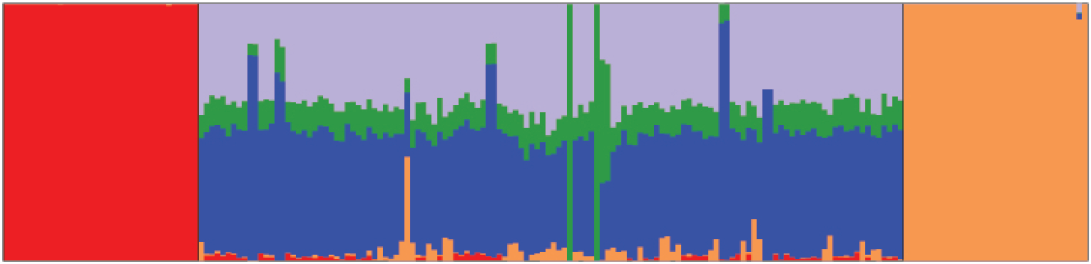


**Figure S5.** Pairwise F_ST_ between each species pair at 47,594 biallelic variant loci. The three different species were treated as populations. Values of the boxes are (25% quartile, median, 75% quartile): 0.02, 0.06, and 0.17; 0.01, 0.02, and 0.06; 0.01, 0.02, and 0.05. Mean values are shown as grey dots: 0.16, 0.07, and 0.04. Figure created in R.

**Figure S6.** Estimated genetic admixture of 177 *Betula* samples for which both microsatellite (upper panel) and RAD data (lower panel) was available. Same individuals are aligned. Colours used correspond to Figure 4 in the main text. Figure created with ‘distruct1.1’.
