# Supplementary material for: Unidirectional diploid–tetraploid introgression among British birch trees with shifting ranges shown by restriction site‐associated markers
Source: Mol Ecol. 2016 May 11;25(11):2413–26. doi: 10.1111/mec.13644 (PMC4999052; doi:10.1111/mec.13644)
Supplement: Supplementary file 2 [file MEC-25-2413-s002.pdf]

nana_09710_ACACG_1t_genotypes.txt

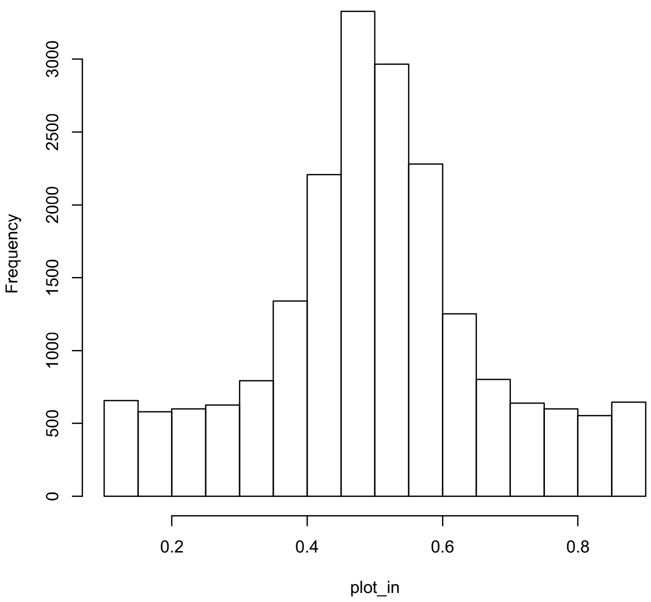

nana_1029x_TAGCTGAT_genotypes.txt

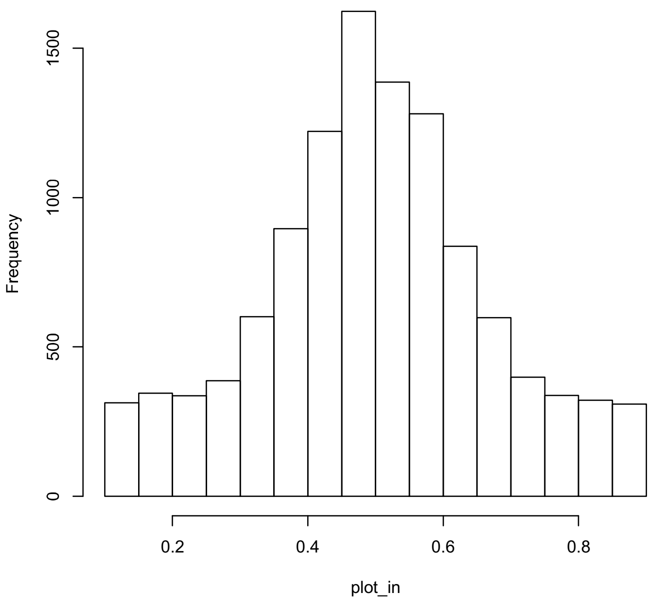

nana_1090x_TCAGCATC_genotypes.txt

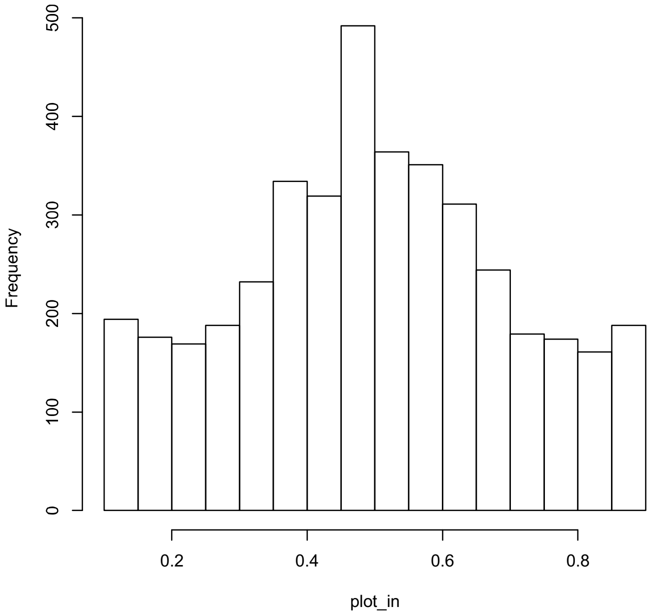

nana_1214x_CTGATGCT_genotypes.txt

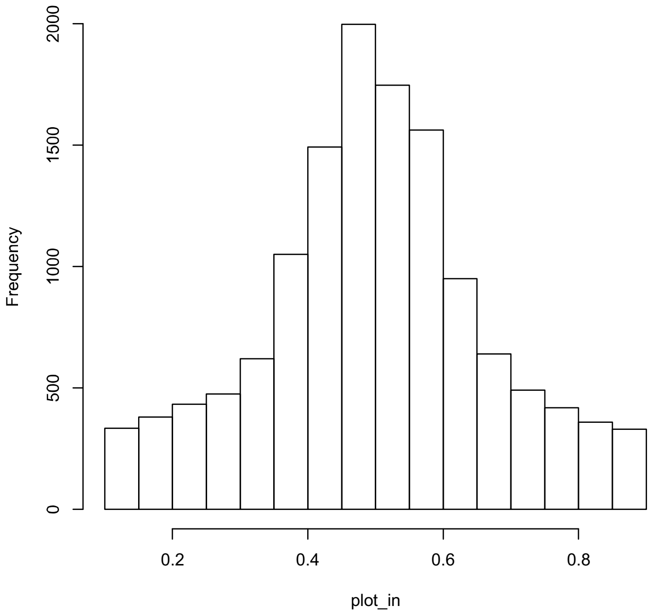

nana_1224x_GACTCATC_genotypes.txt

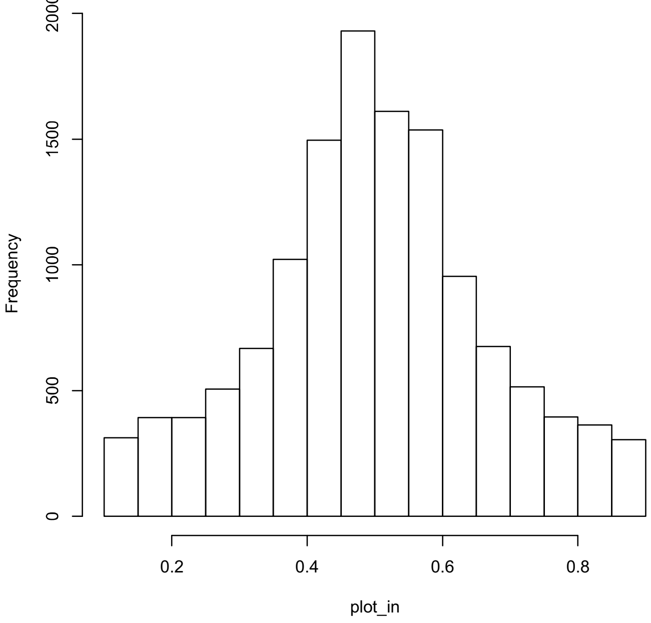

nana_1247x_GCTAGACG_genotypes.txt

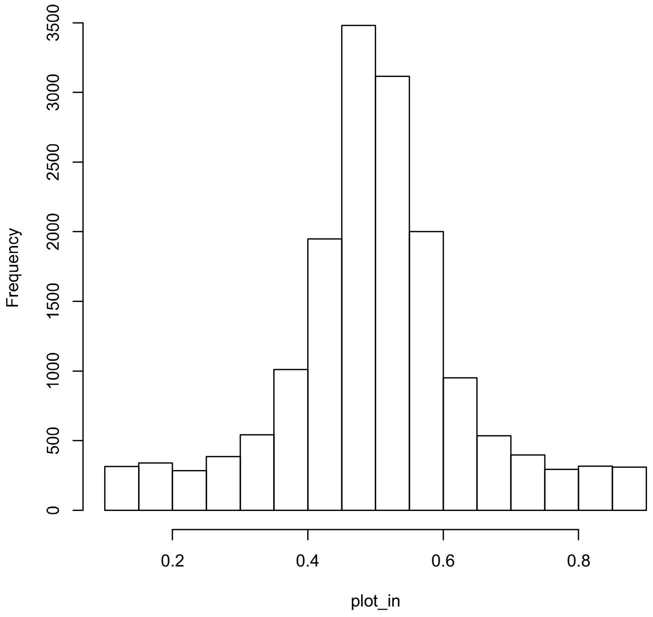

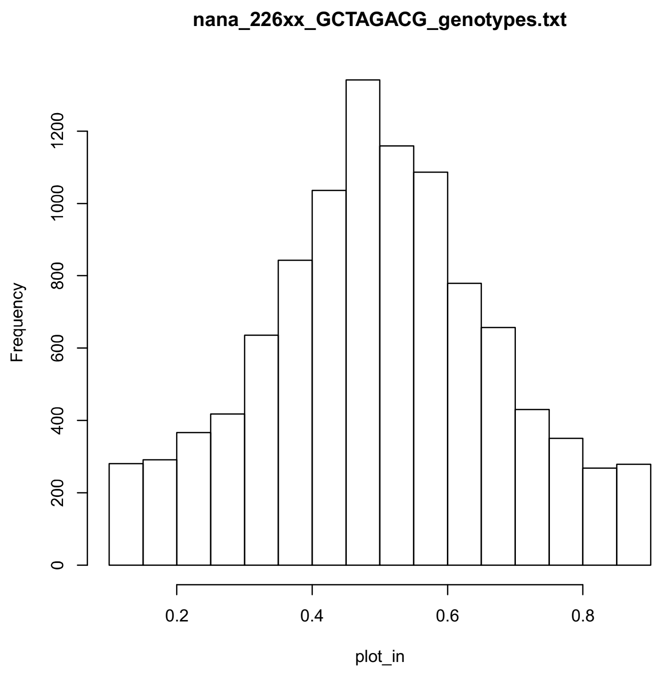
nana_226xx_GCTAGACG_genotypes.txt
Frequency
0
200
400
600
800
1000
1200
0.2
0.4
0.6
0.8
plot_in

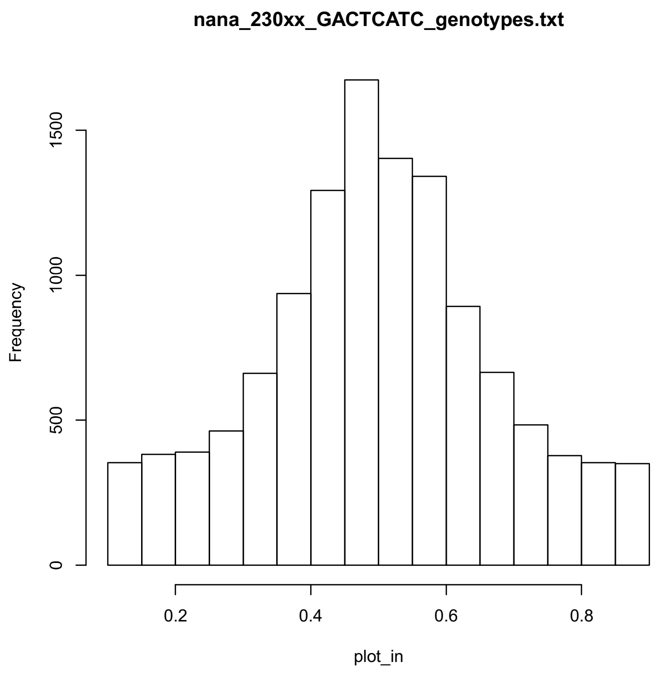
nana_230xx_GACTCATC_genotypes.txt
Frequency
0
500
1000
1500
0.2
0.4
0.6
0.8
plot_in

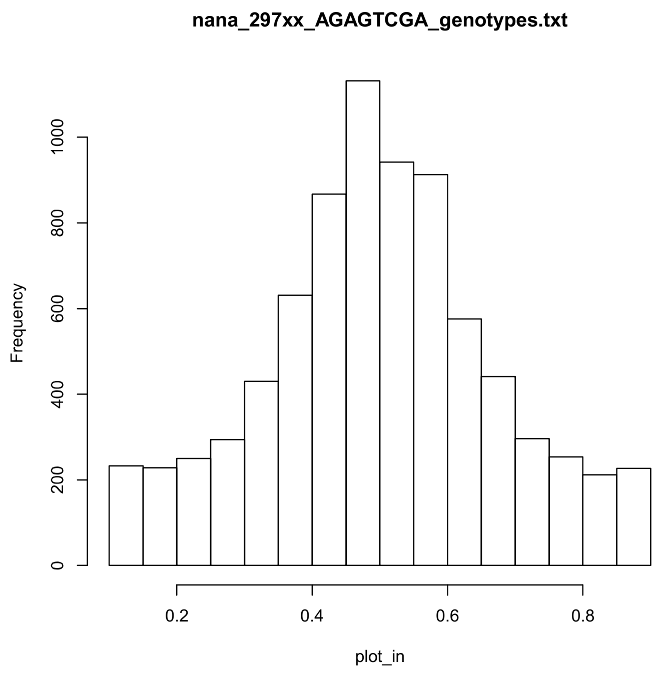
nana_297xx_AGAGTCGA_genotypes.txt
Frequency
0
200
400
600
800
1000
0.2
0.4
0.6
0.8
plot_in

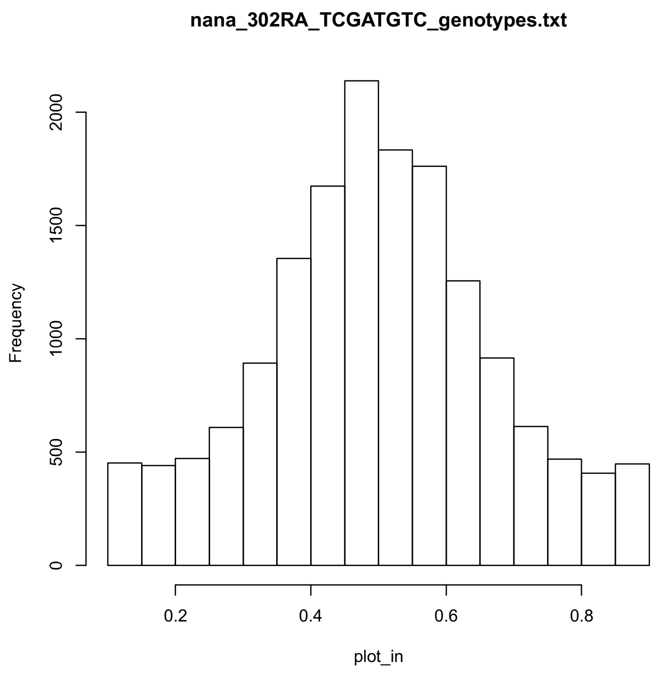
nana_302RA_TCGATGTC_genotypes.txt
Frequency
0
500
1000
1500
2000
0.2
0.4
0.6
0.8
plot_in

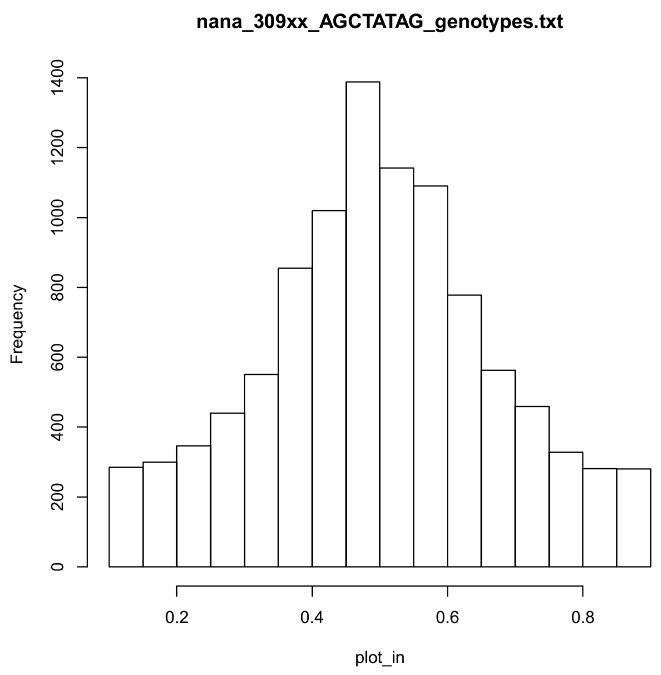
nana_309xx_AGCTATAG_genotypes.txt
Frequency
0
200
400
600
800
1000
1200
1400
0.2
0.4
0.6
0.8
plot_in

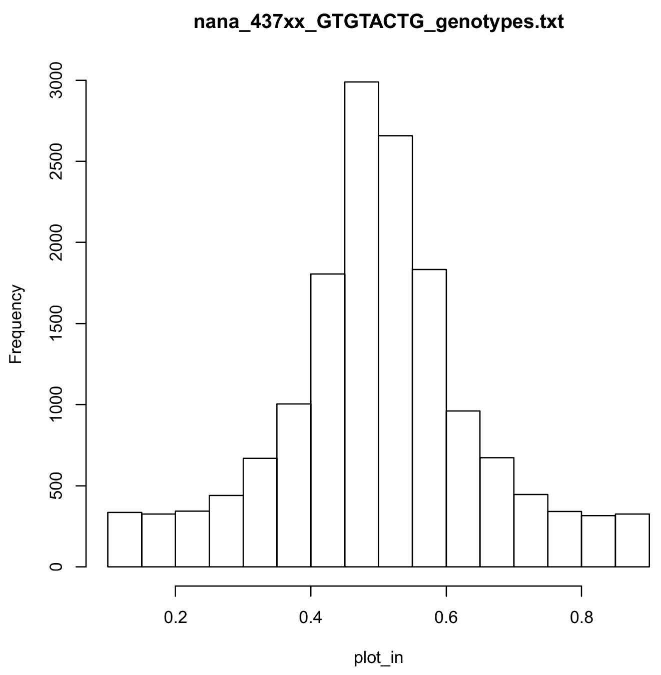
nana_437xx_GTGTACTG_genotypes.txt
Frequency
0
500
1000
1500
2000
2500
3000
0.2
0.4
0.6
0.8
plot_in

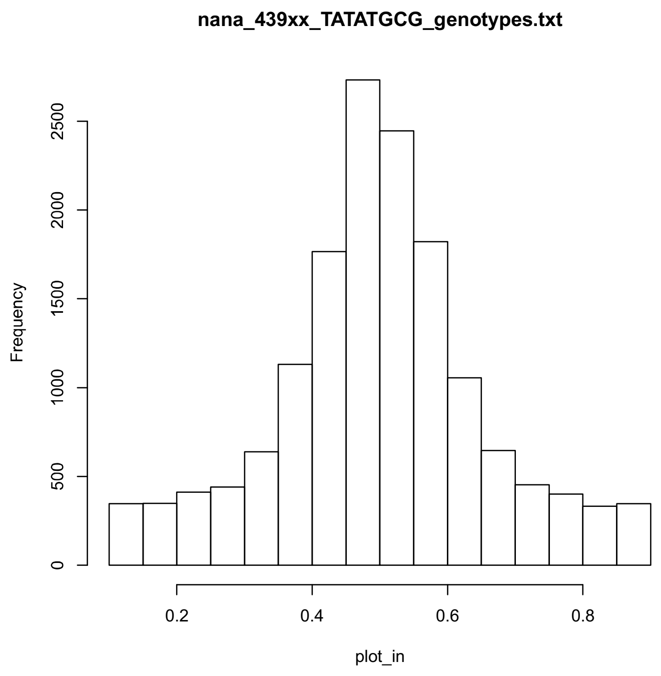
nana_439xx_TATATGCG_genotypes.txt
Frequency
0
500
1000
1500
2000
2500
0.2
0.4
0.6
0.8
plot_in

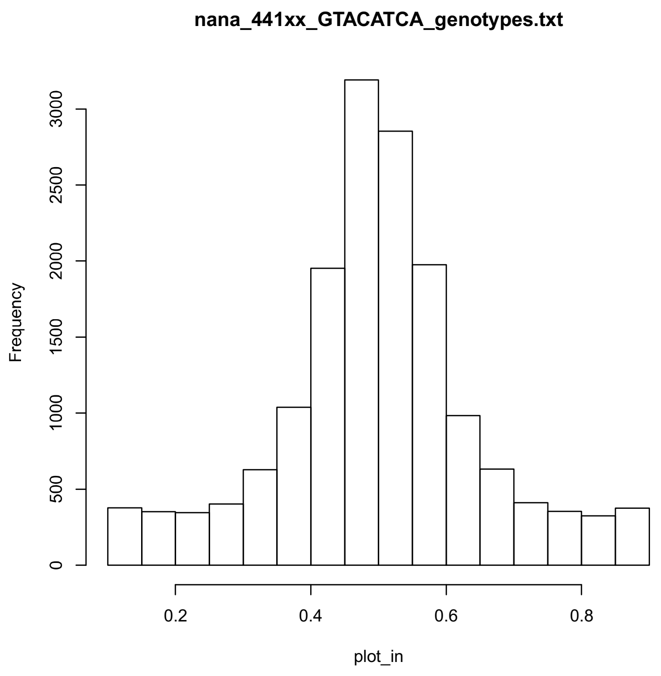
nana_441xx_GTACATCA_genotypes.txt
Frequency
0
500
1000
1500
2000
2500
3000
0.2
0.4
0.6
0.8
plot_in

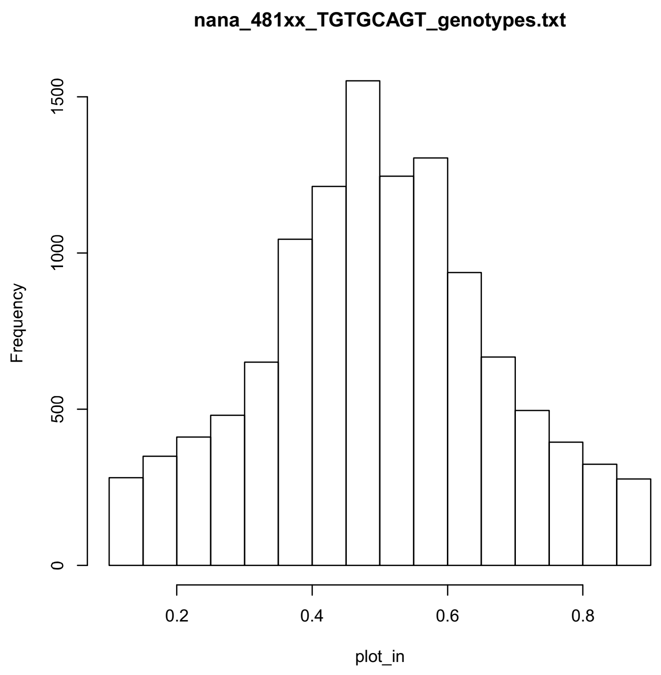
nana_481xx_TGTGCAGT_genotypes.txt
Frequency
0
500
1000
1500
0.2
0.4
0.6
0.8
plot_in

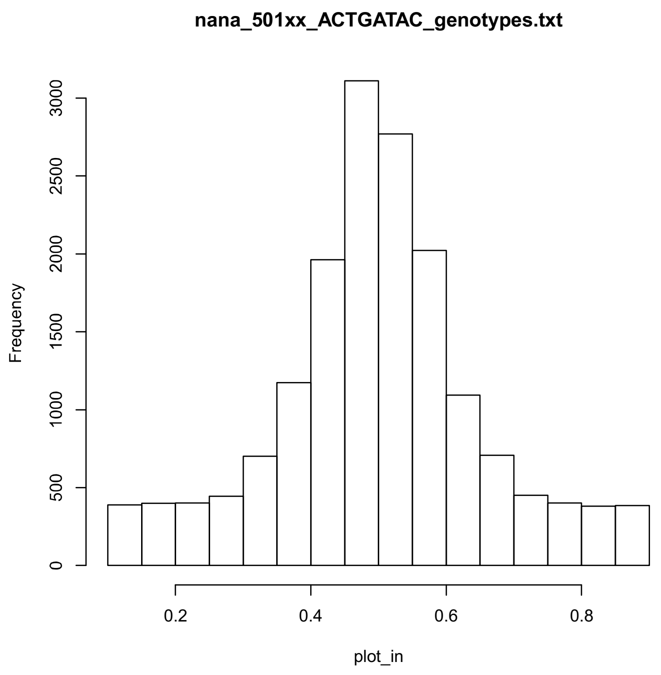
nana_501xx_ACTGATAC_genotypes.txt
Frequency
0
500
1000
1500
2000
2500
3000
0.2
0.4
0.6
0.8
plot_in

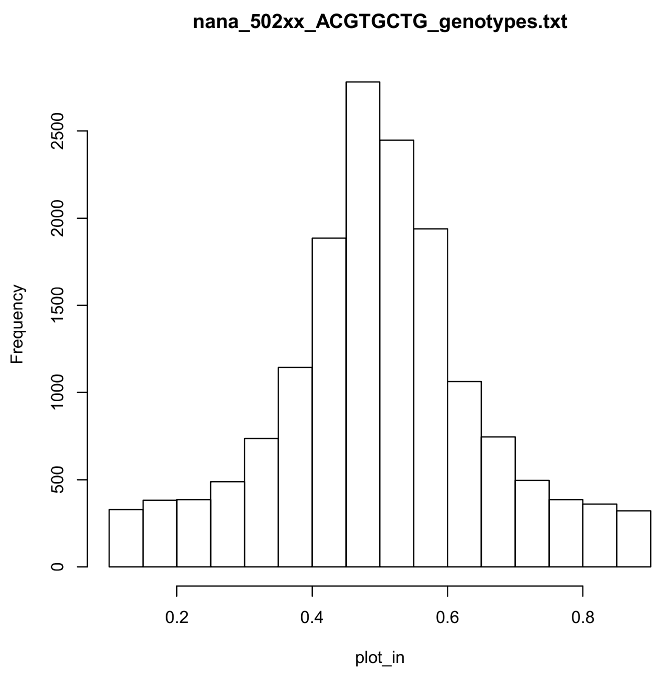
nana_502xx_ACGTGCTG_genotypes.txt
Frequency
0
500
1000
1500
2000
2500
0.2
0.4
0.6
0.8
plot_in

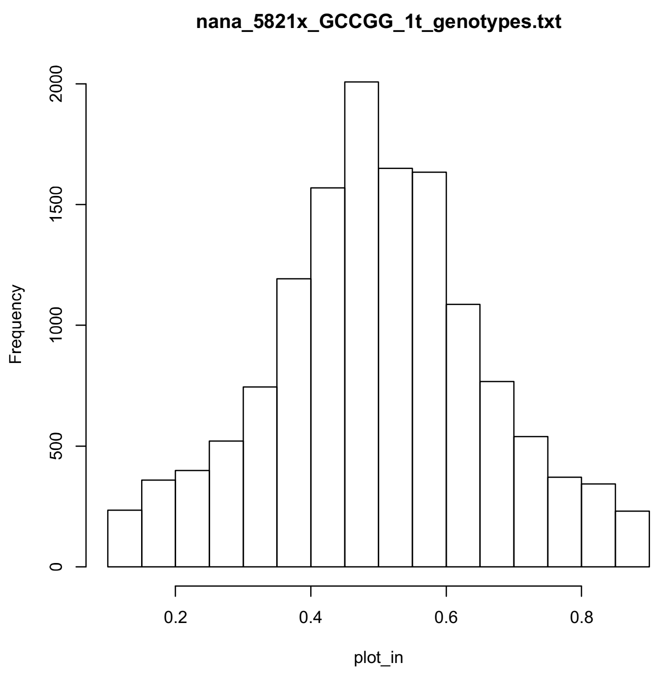
nana_5821x_GCCGG_1t_genotypes.txt
Frequency
0
500
1000
1500
2000
0.2
0.4
0.6
0.8
plot_in

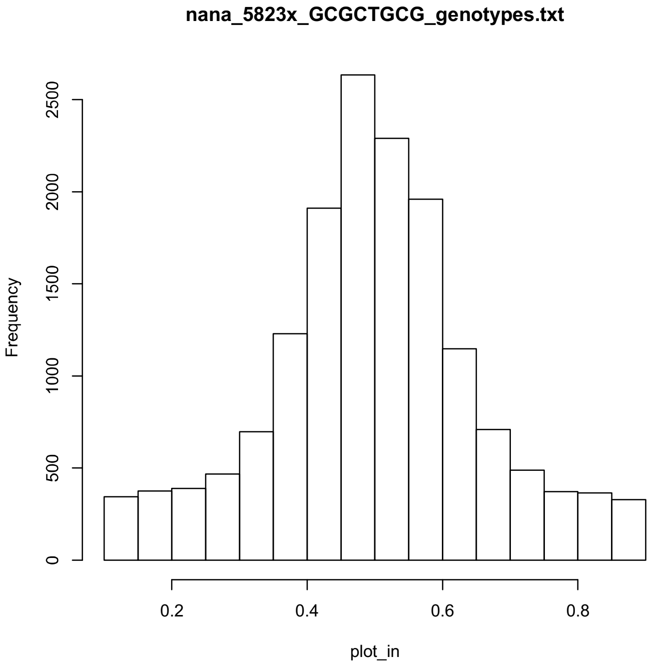
nana_5823x_GCGCTGCG_genotypes.txt
Frequency
0
500
1000
1500
2000
2500
0.2
0.4
0.6
0.8
plot_in

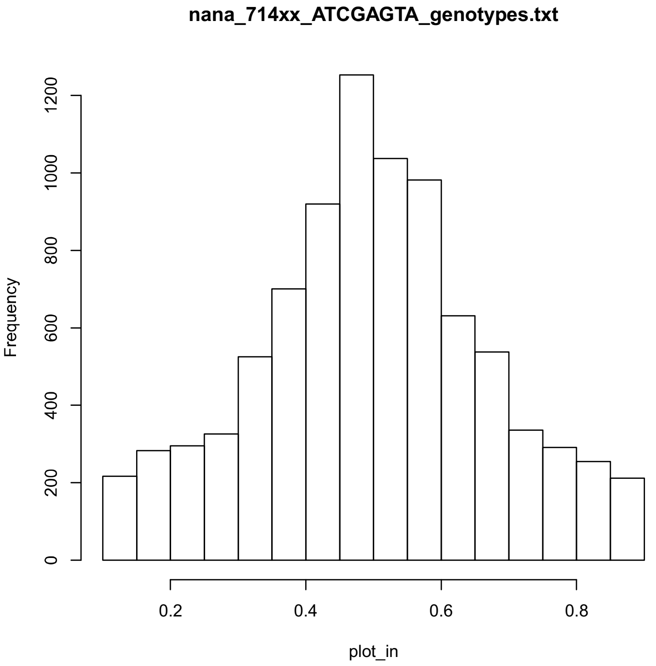
nana_714xx_ATCGAGTA_genotypes.txt
Frequency
0
200
400
600
800
1000
1200
0.2
0.4
0.6
0.8
plot_in

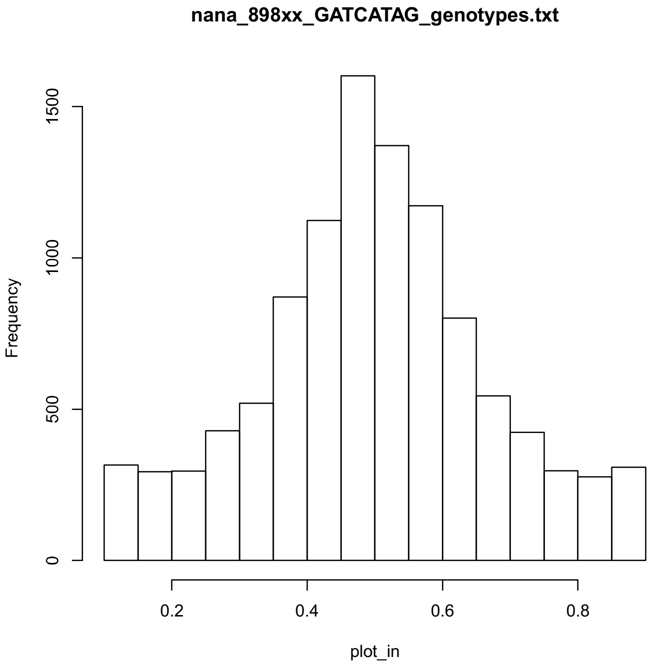
nana_898xx_GATCATAG_genotypes.txt
Frequency
0
500
1000
1500
0.2
0.4
0.6
0.8
plot_in

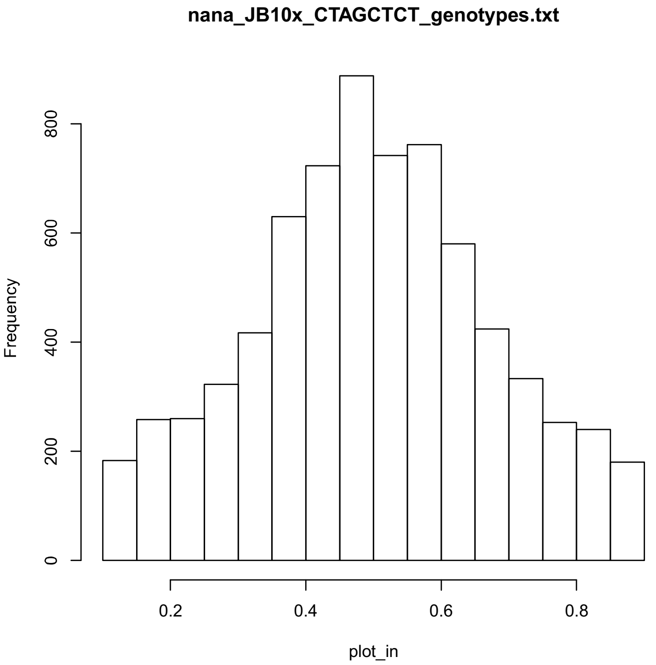
nana_JB10x_CTAGCTCT_genotypes.txt
Frequency
0
200
400
600
800
0.2
0.4
0.6
0.8
plot_in

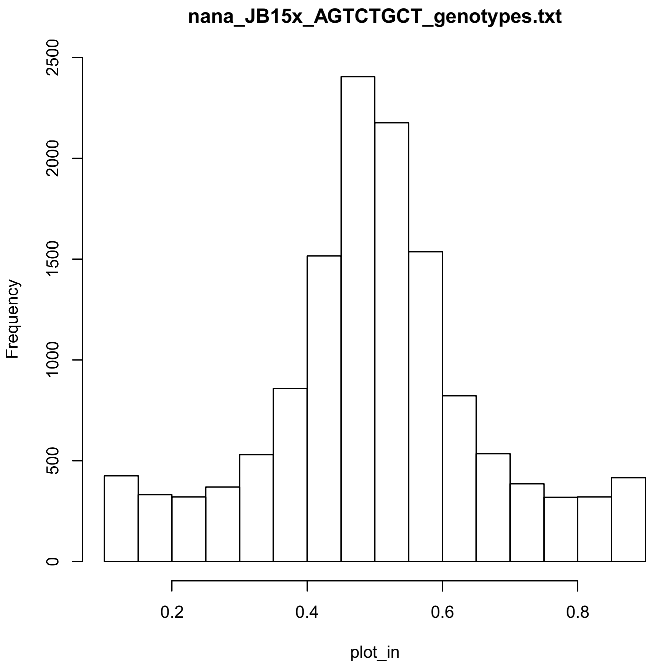
nana_JB15x_AGTCTGCT_genotypes.txt
Frequency
0
500
1000
1500
2000
2500
0.2
0.4
0.6
0.8
plot_in

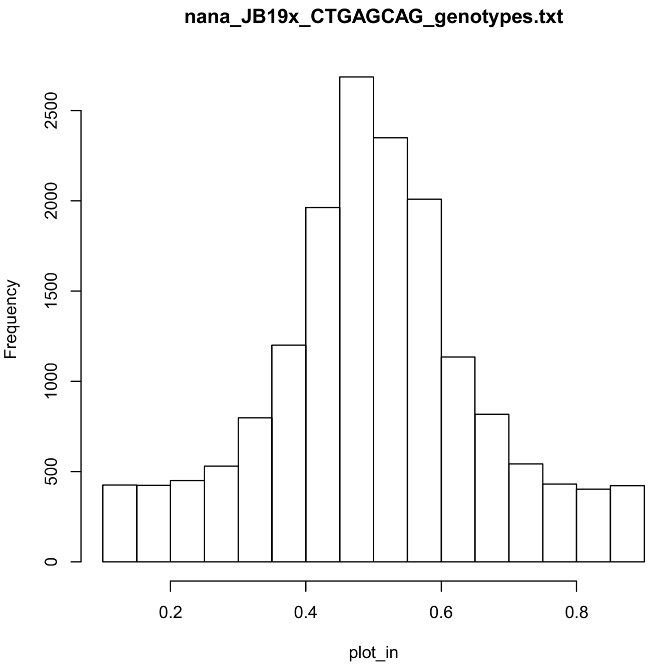
nana_JB19x_CTGAGCAG_genotypes.txt
Frequency
0
500
1000
1500
2000
2500
0.2
0.4
0.6
0.8
plot_in

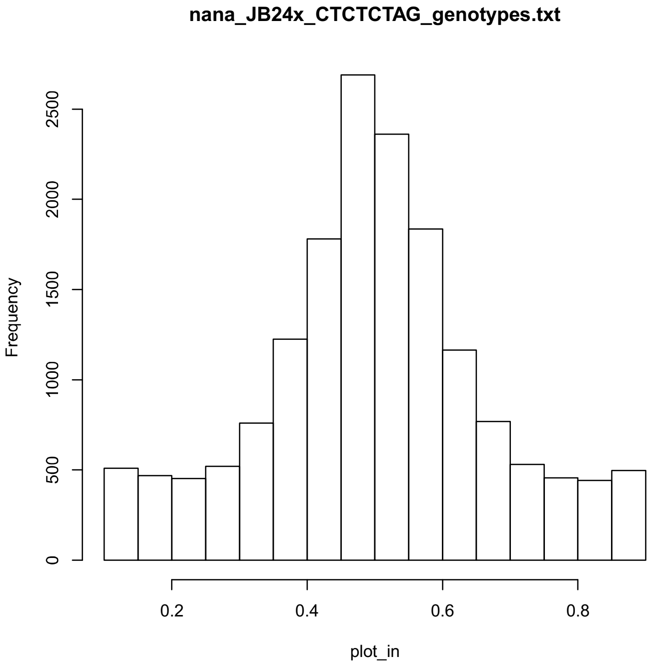
nana_JB24x_CTCTCTAG_genotypes.txt
Frequency
0
500
1000
1500
2000
2500
0.2
0.4
0.6
0.8
plot_in

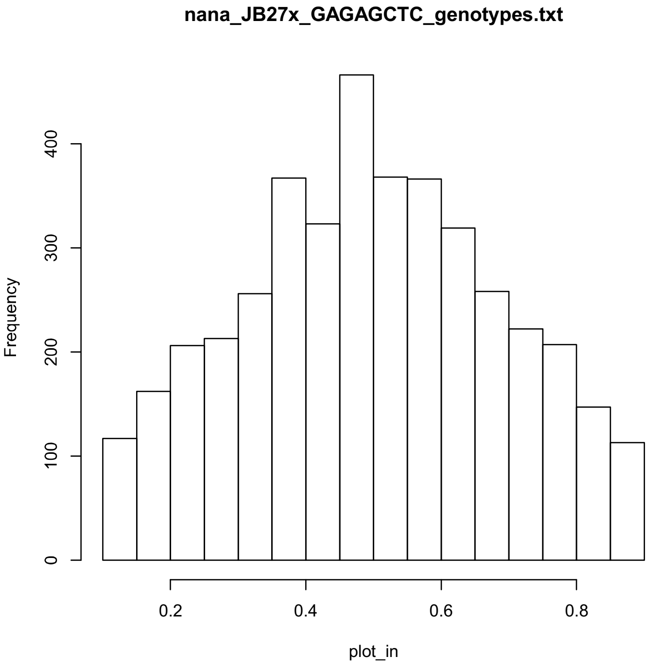
nana_JB27x_GAGAGCTC_genotypes.txt
Frequency
0
100
200
300
400
0.2
0.4
0.6
0.8
plot_in

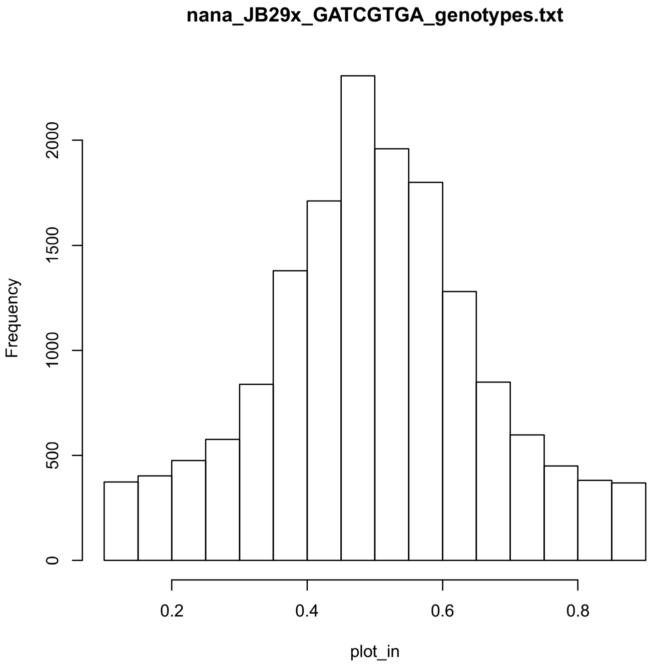
nana_JB29x_GATCGTGA_genotypes.txt
Frequency
0
500
1000
1500
2000
0.2
0.4
0.6
0.8
plot_in

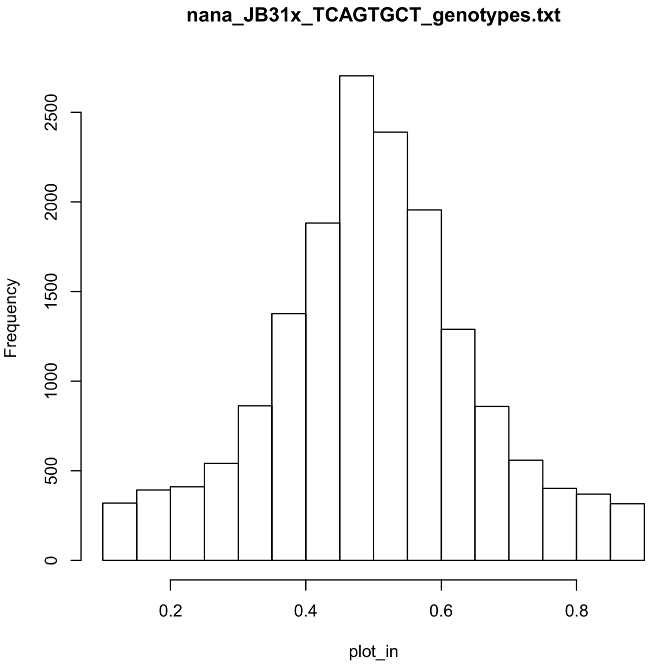
nana_JB31x_TCAGTGCT_genotypes.txt
Frequency
0
500
1000
1500
2000
2500
0.2
0.4
0.6
0.8
plot_in

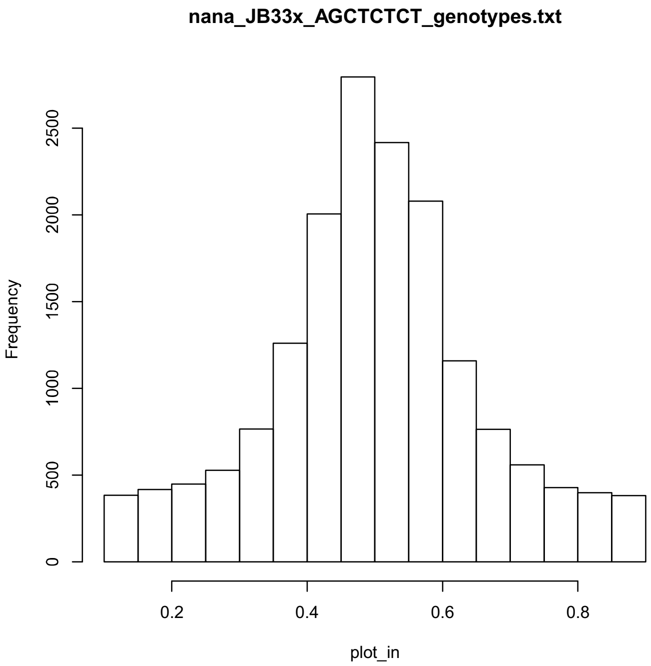
nana_JB33x_AGCTCTCT_genotypes.txt
Frequency
0
500
1000
1500
2000
2500
0.2
0.4
0.6
0.8
plot_in

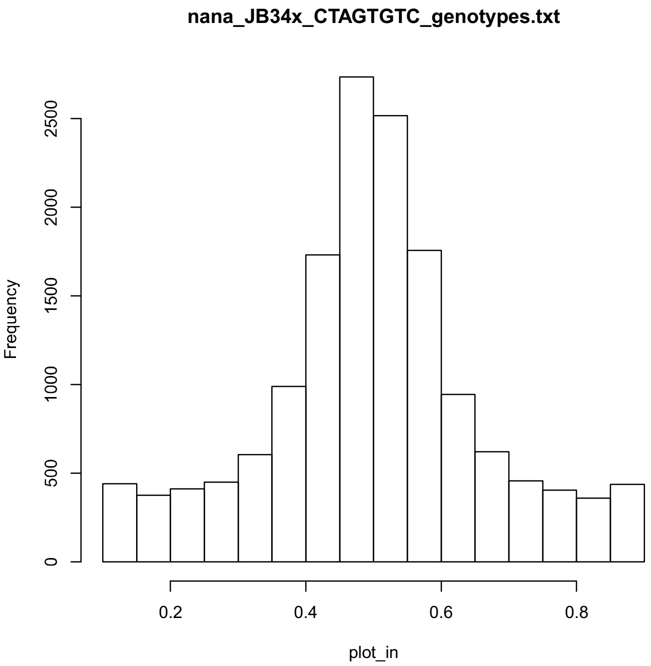
nana_JB34x_CTAGTGTC_genotypes.txt
Frequency
0
500
1000
1500
2000
2500
0.2
0.4
0.6
0.8
plot_in

nana_JB36x_AGTCGCAG_genotypes.txt

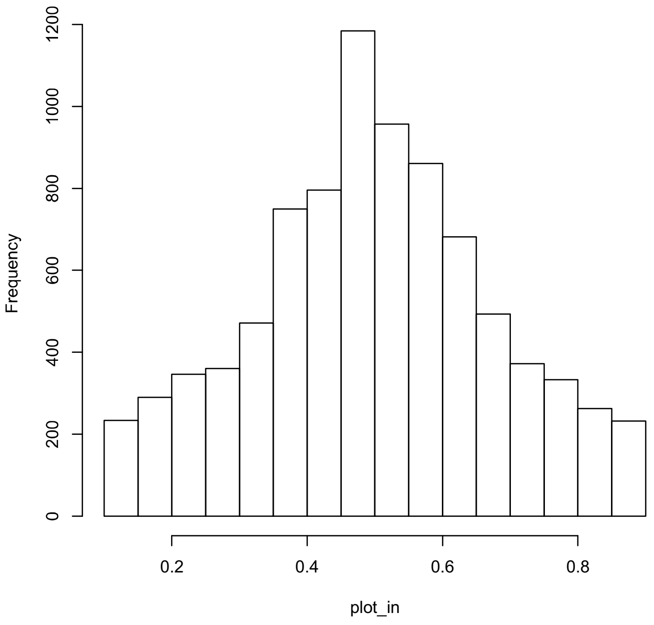

nana_JB39x_TCGACTCT_genotypes.txt

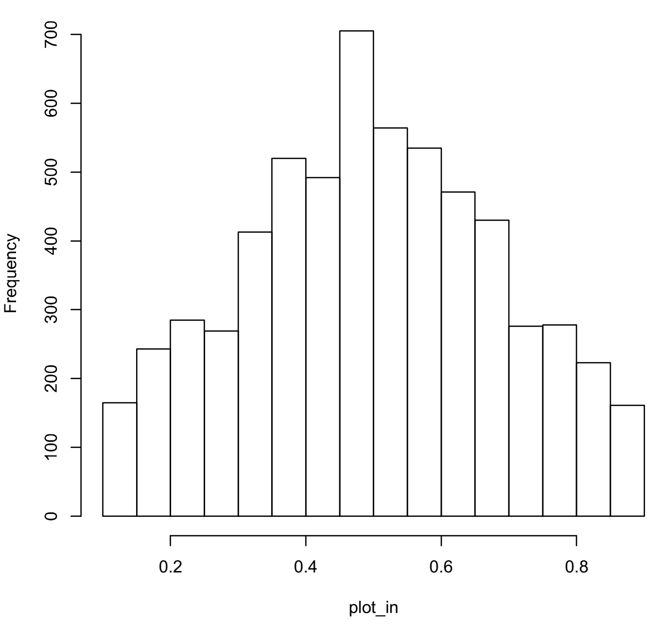

nana_JB42x_CTAGATAG_genotypes.txt

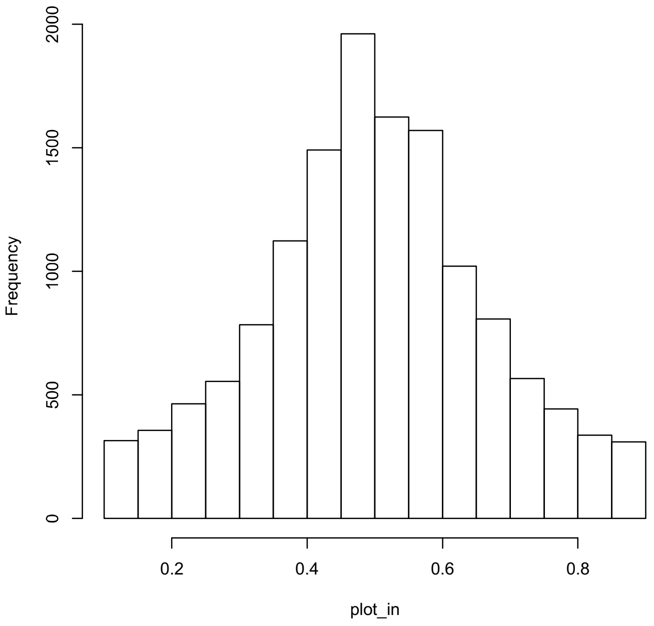

nana_JB4xx_AGTCACGA_genotypes.txt

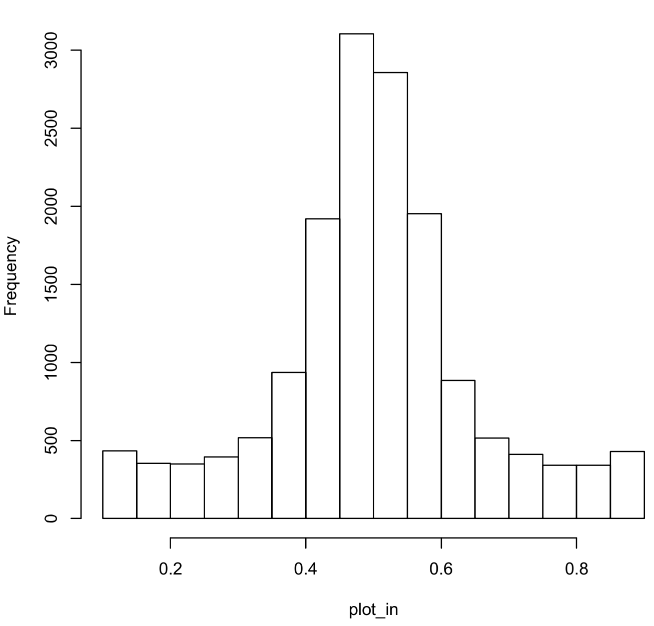

nana_JB7xx_TCGAGTGA_genotypes.txt

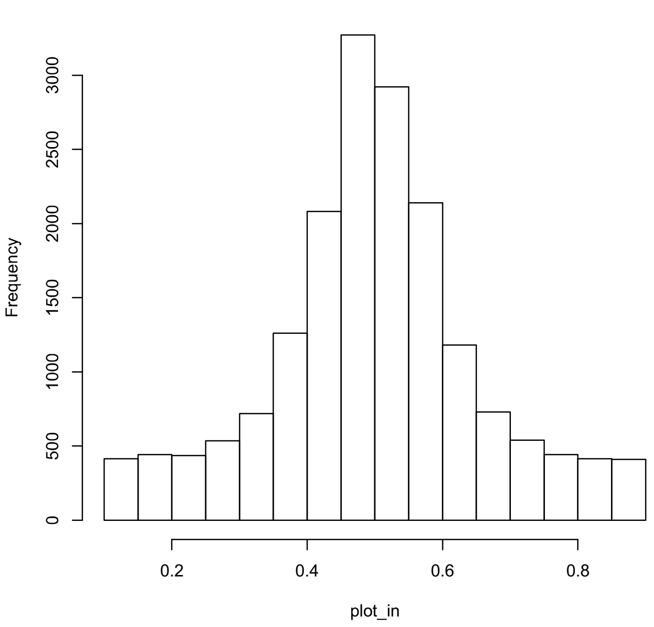

nana_JB8xx_TAGCGACG_genotypes.txt

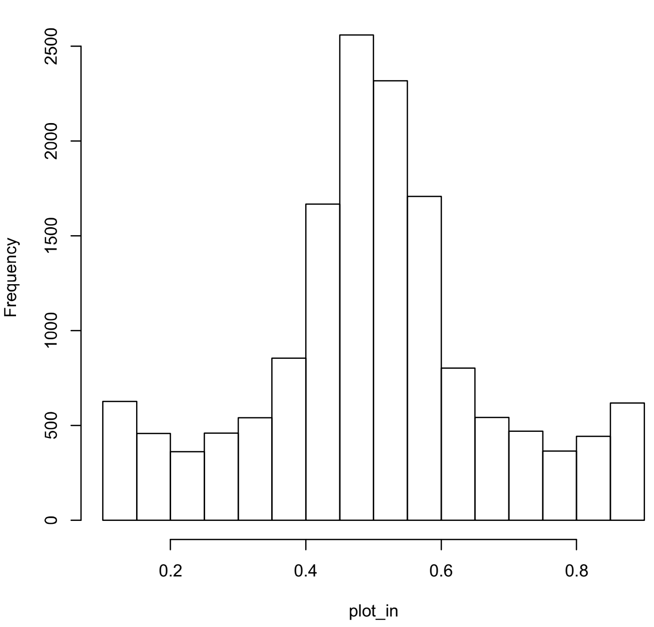

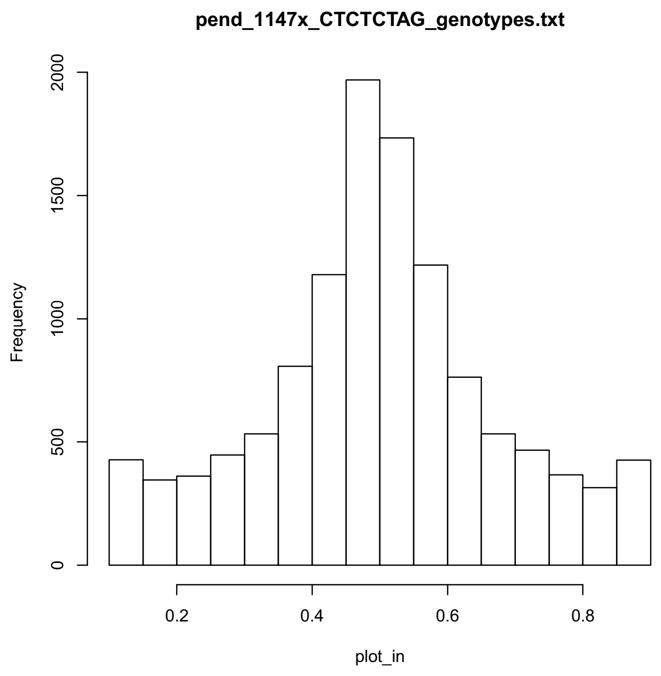
pend_1147x_CTCTCTAG_genotypes.txt
Frequency
0
500
1000
1500
2000
0.2
0.4
0.6
0.8
plot_in

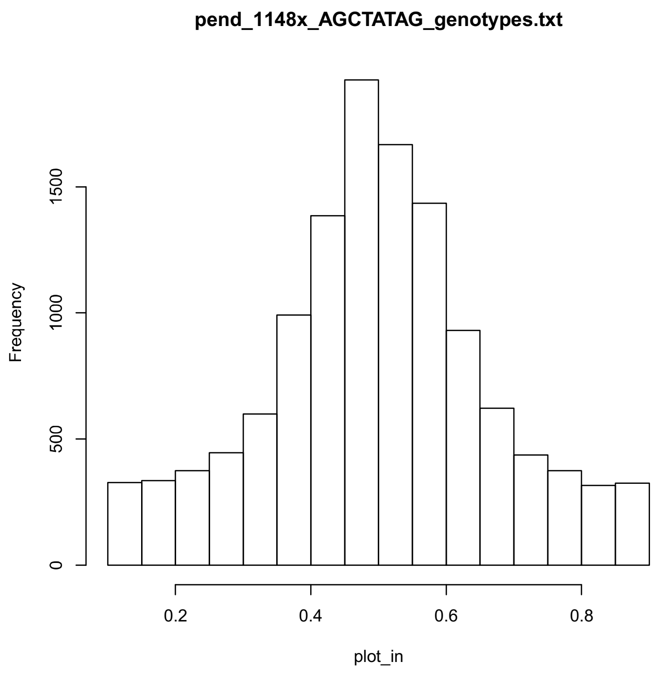
pend_1148x_AGCTATAG_genotypes.txt
Frequency
0
500
1000
1500
0.2
0.4
0.6
0.8
plot_in

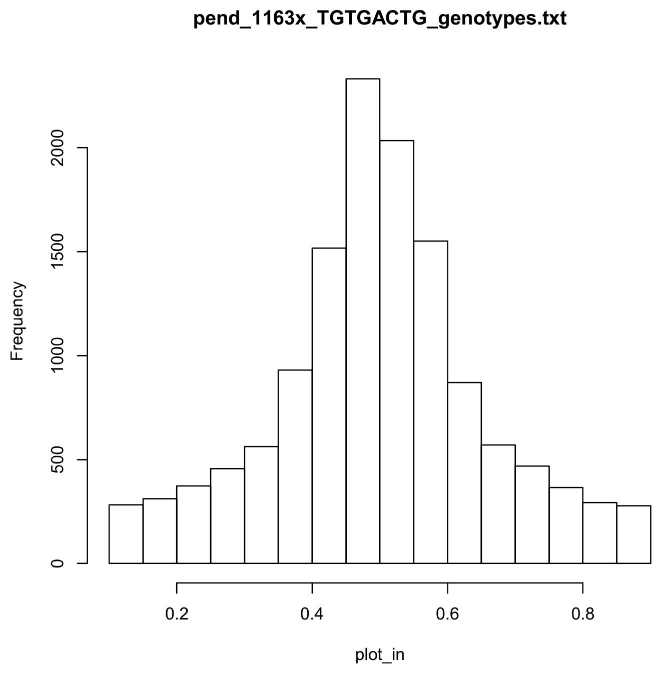
pend_1163x_TGTGACTG_genotypes.txt
Frequency
0
500
1000
1500
2000
0.2
0.4
0.6
0.8
plot_in

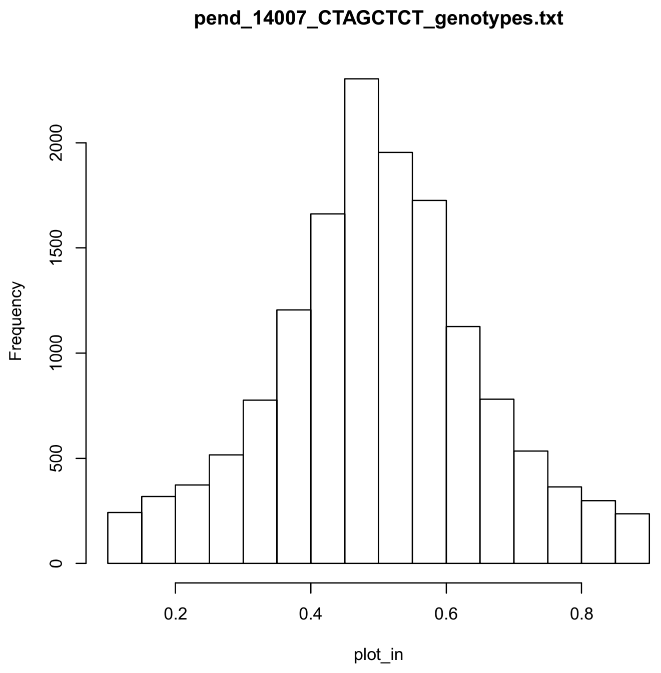
pend_14007_CTAGCTCT_genotypes.txt
Frequency
0
500
1000
1500
2000
0.2
0.4
0.6
0.8
plot_in

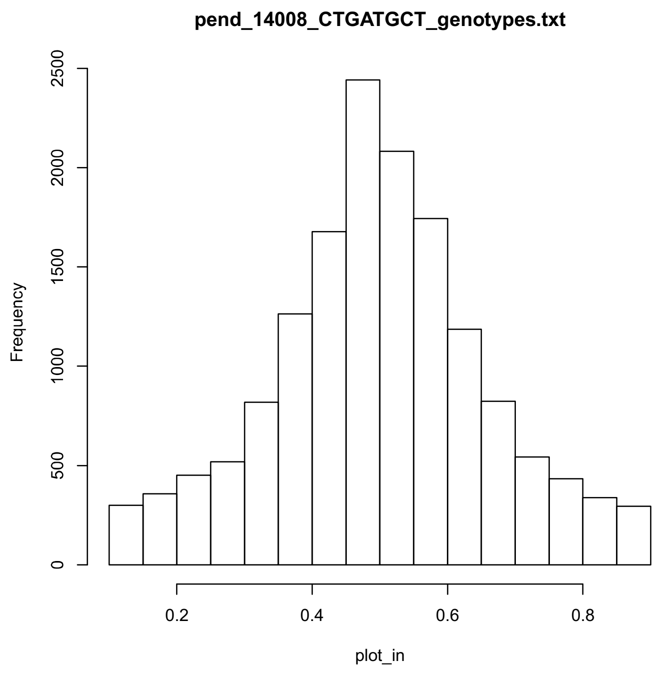
pend_14008_CTGATGCT_genotypes.txt
Frequency
0
500
1000
1500
2000
2500
0.2
0.4
0.6
0.8
plot_in

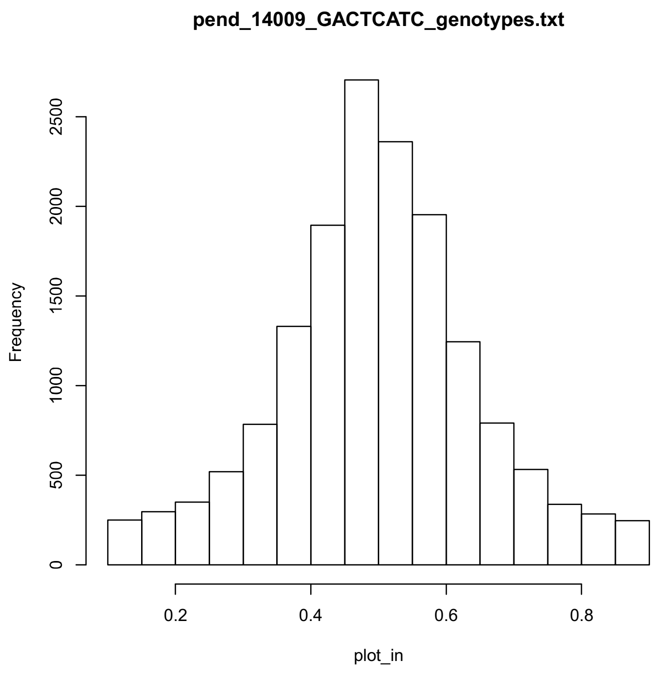
pend_14009_GACTCATC_genotypes.txt
Frequency
0
500
1000
1500
2000
2500
0.2
0.4
0.6
0.8
plot_in

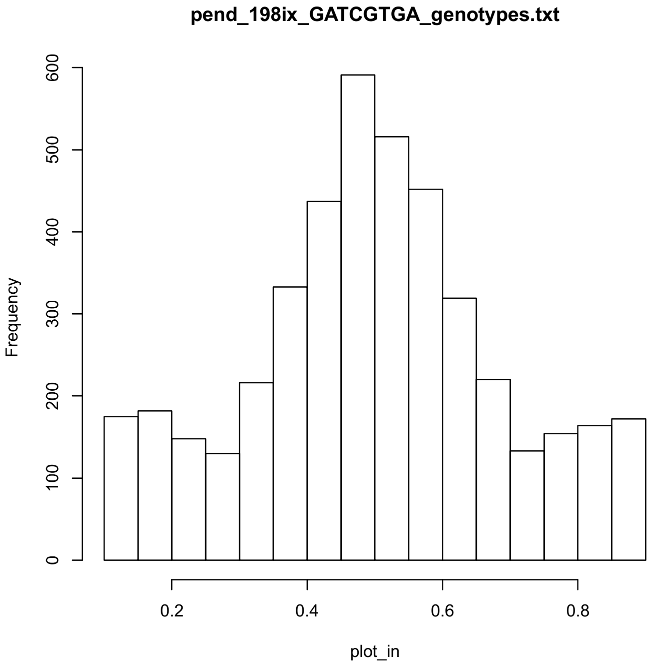
pend_198ix_GATCGTGA_genotypes.txt
Frequency
0
100
200
300
400
500
600
0.2
0.4
0.6
0.8
plot_in

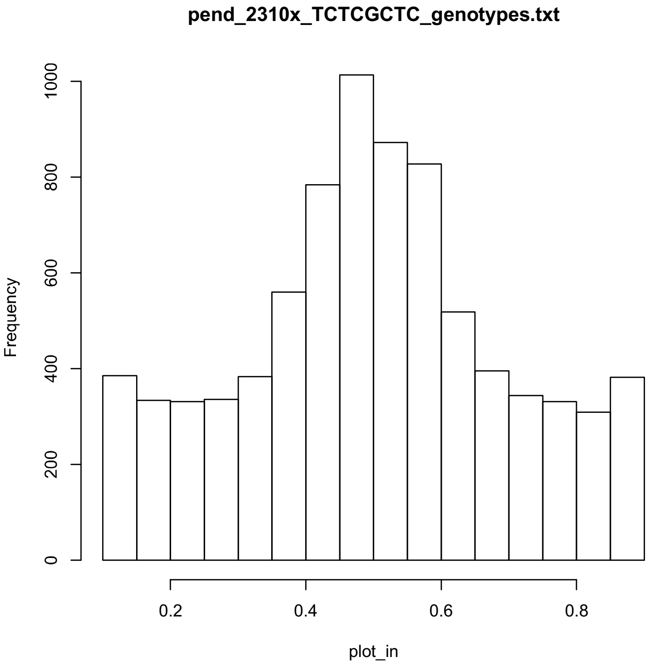
pend_2310x_TCTCGCTC_genotypes.txt
Frequency
0
200
400
600
800
1000
0.2
0.4
0.6
0.8
plot_in

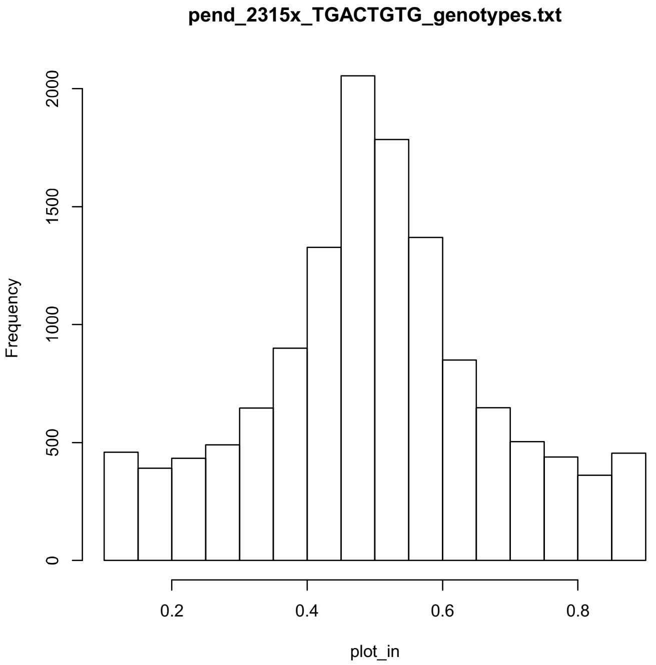
pend_2315x_TGACTGTG_genotypes.txt
Frequency
0
500
1000
1500
2000
0.2
0.4
0.6
0.8
plot_in

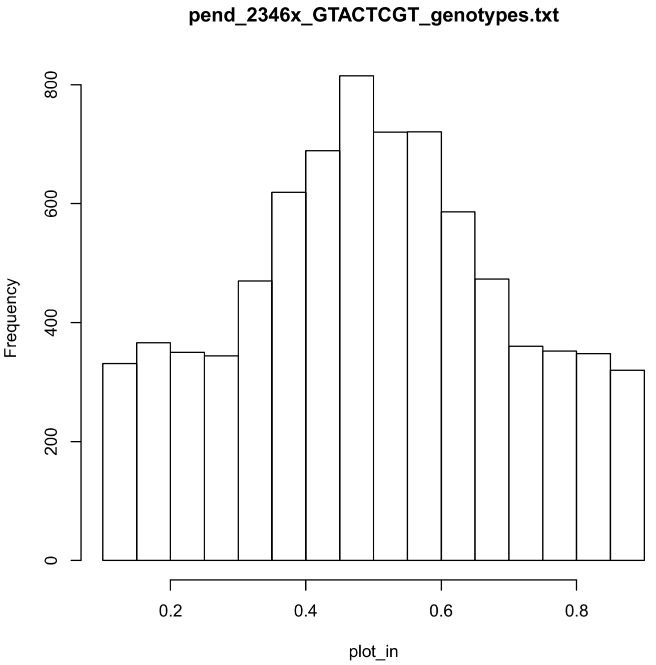
pend_2346x_GTACTCGT_genotypes.txt
Frequency
0
200
400
600
800
0.2
0.4
0.6
0.8
plot_in

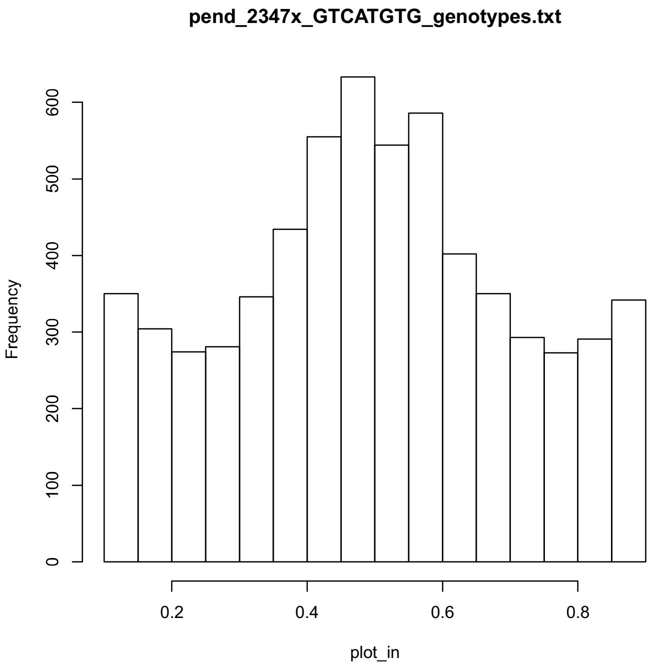
pend_2347x_GTCATGTG_genotypes.txt
Frequency
0
100
200
300
400
500
600
0.2
0.4
0.6
0.8
plot_in

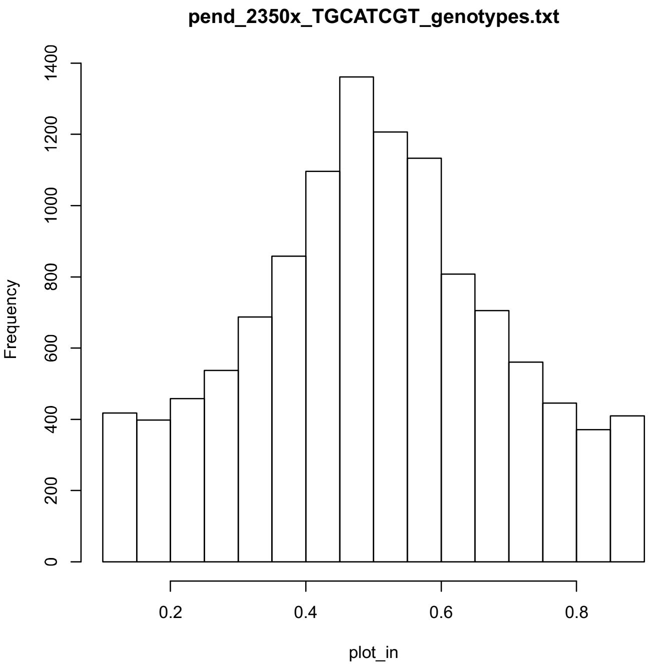
pend_2350x_TGCATCGT_genotypes.txt
Frequency
0
200
400
600
800
1000
1200
1400
0.2
0.4
0.6
0.8
plot_in

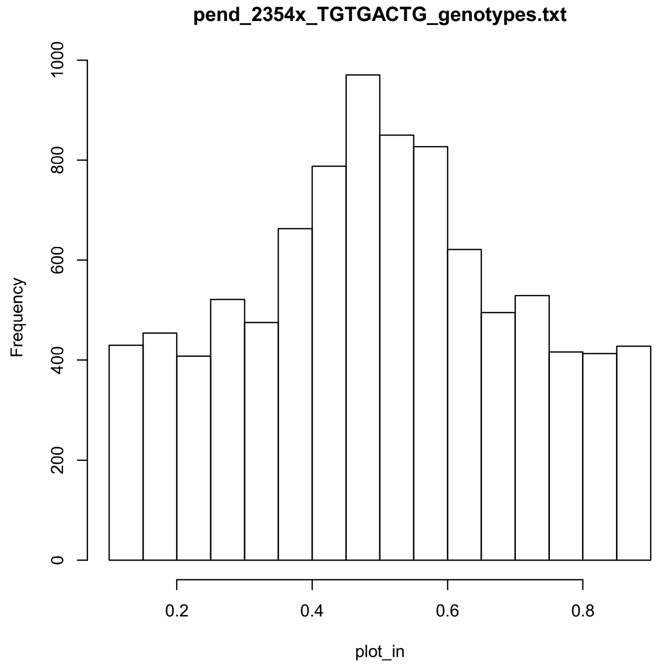
pend_2354x_TGTGACTG_genotypes.txt
Frequency
0
200
400
600
800
1000
0.2
0.4
0.6
0.8
plot_in

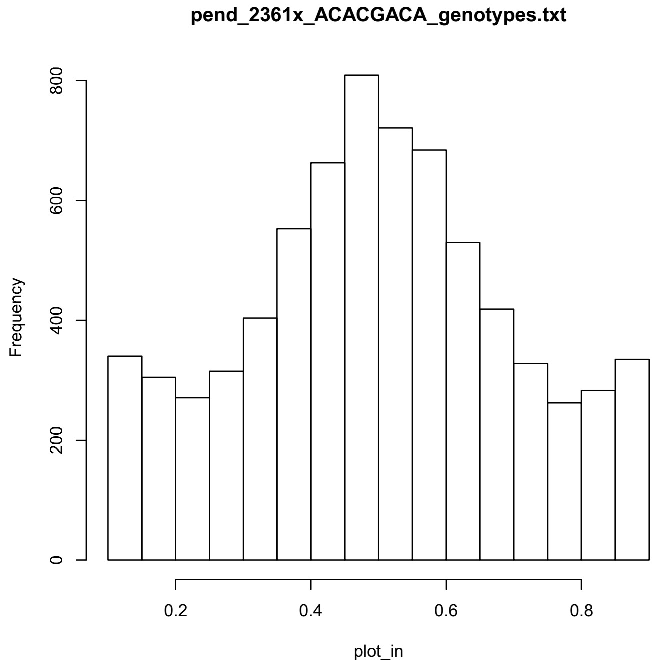
pend_2361x_ACACGACA_genotypes.txt
Frequency
0
200
400
600
800
0.2
0.4
0.6
0.8
plot_in

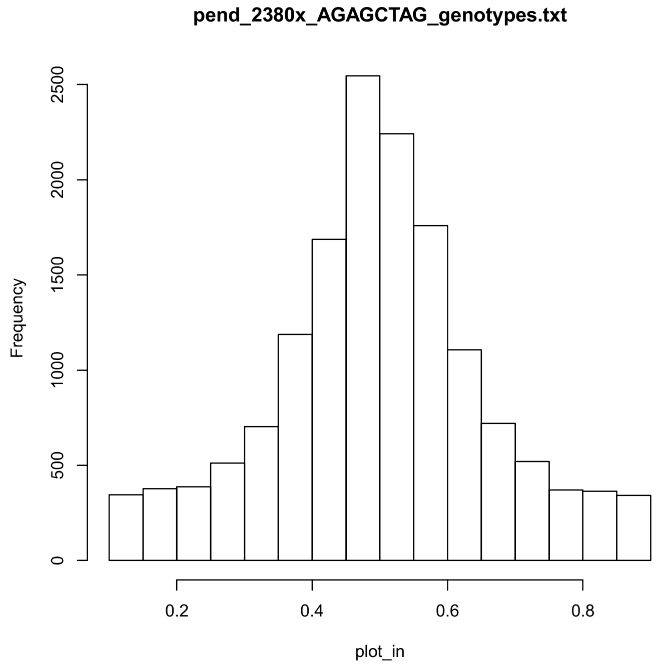
pend_2380x_AGAGCTAG_genotypes.txt
Frequency
0
500
1000
1500
2000
2500
0.2
0.4
0.6
0.8
plot_in

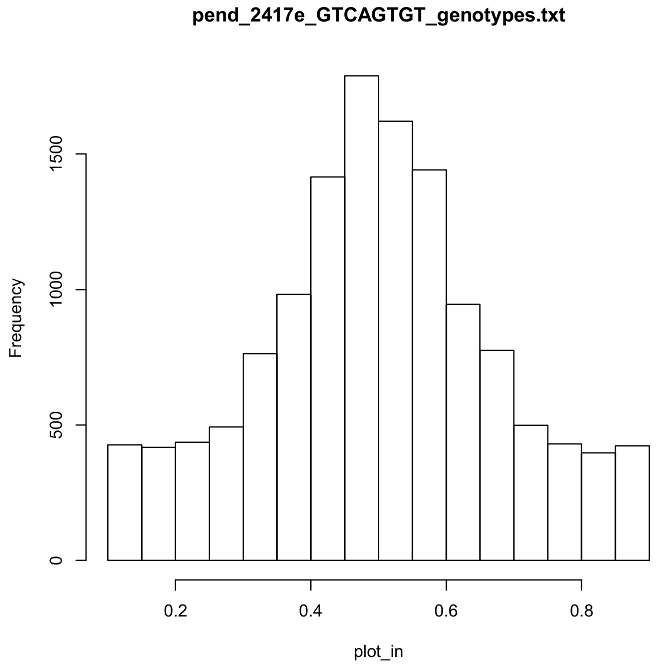
pend_2417e_GTCAGTGT_genotypes.txt
Frequency
0
500
1000
1500
0.2
0.4
0.6
0.8
plot_in

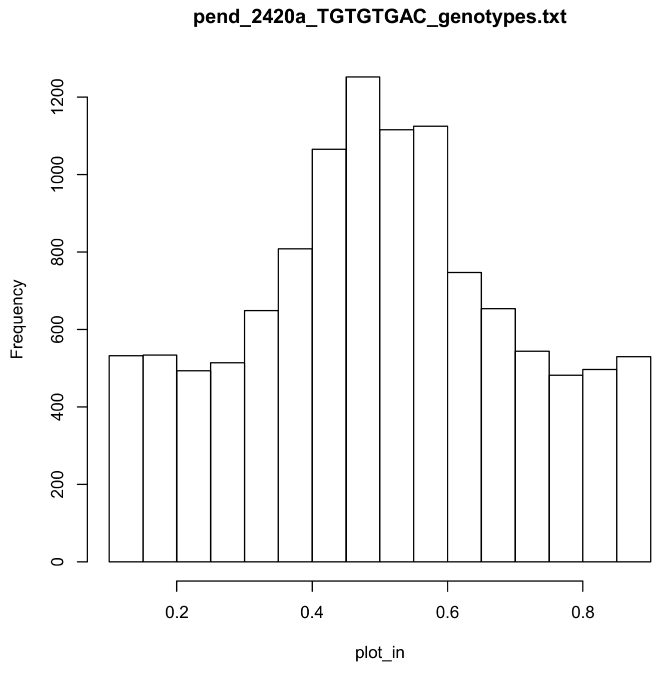
pend_2420a_TGTGTGAC_genotypes.txt
Frequency
0
200
400
600
800
1000
1200
0.2
0.4
0.6
0.8
plot_in

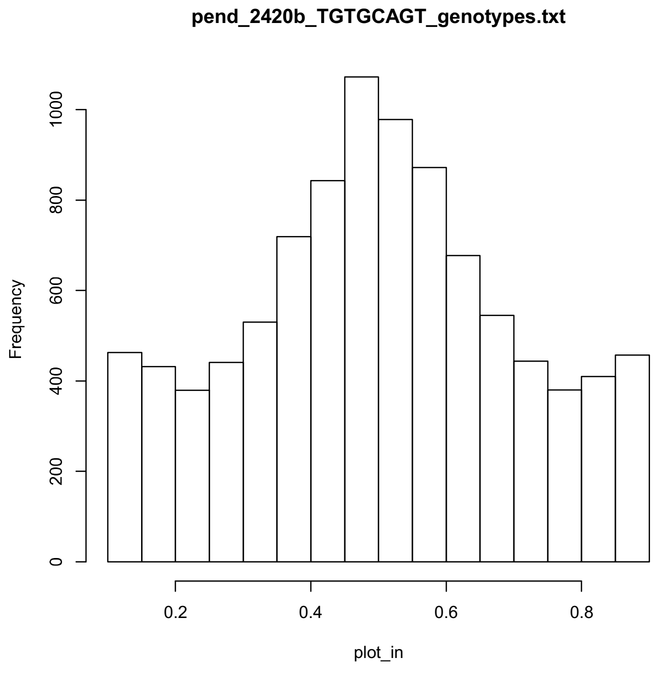
pend_2420b_TGTGCAGT_genotypes.txt
Frequency
0
200
400
600
800
1000
0.2
0.4
0.6
0.8
plot_in

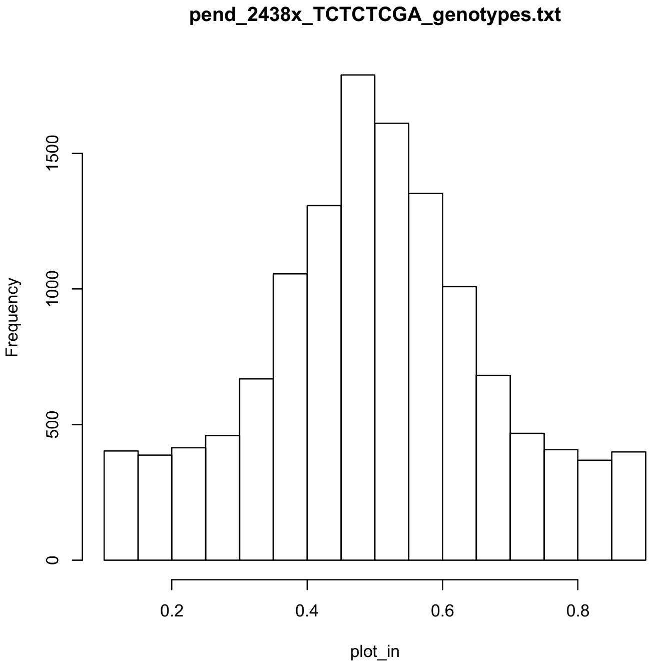
pend_2438x_TCTCTCGA_genotypes.txt
Frequency
0
500
1000
1500
0.2
0.4
0.6
0.8
plot_in

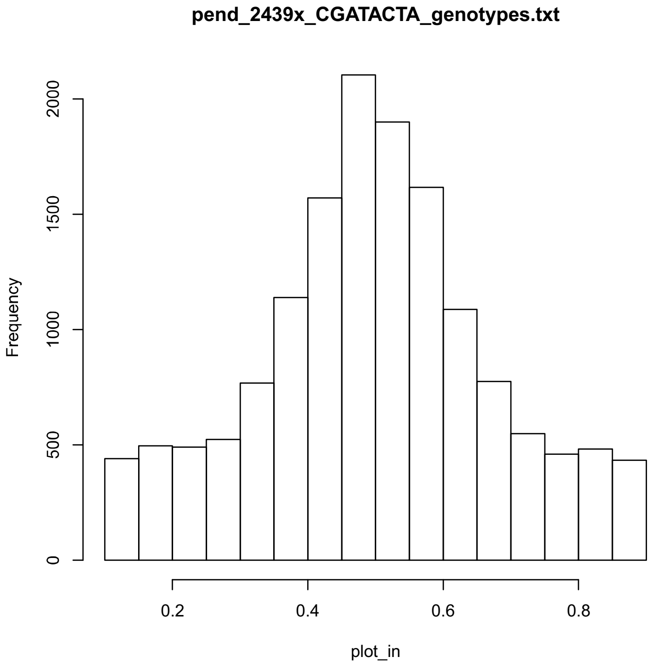
pend_2439x_CGATACTA_genotypes.txt
Frequency
0
500
1000
1500
2000
0.2
0.4
0.6
0.8
plot_in

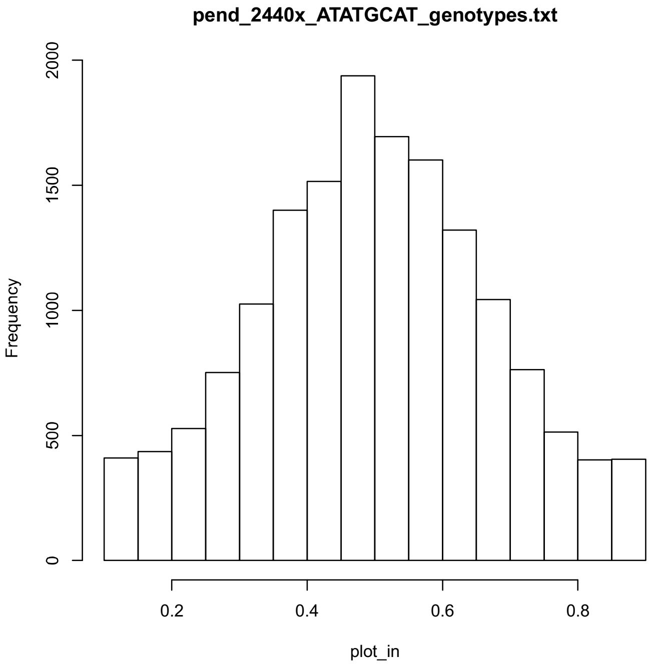
pend_2440x_ATATGCAT_genotypes.txt
Frequency
0
500
1000
1500
2000
0.2
0.4
0.6
0.8
plot_in

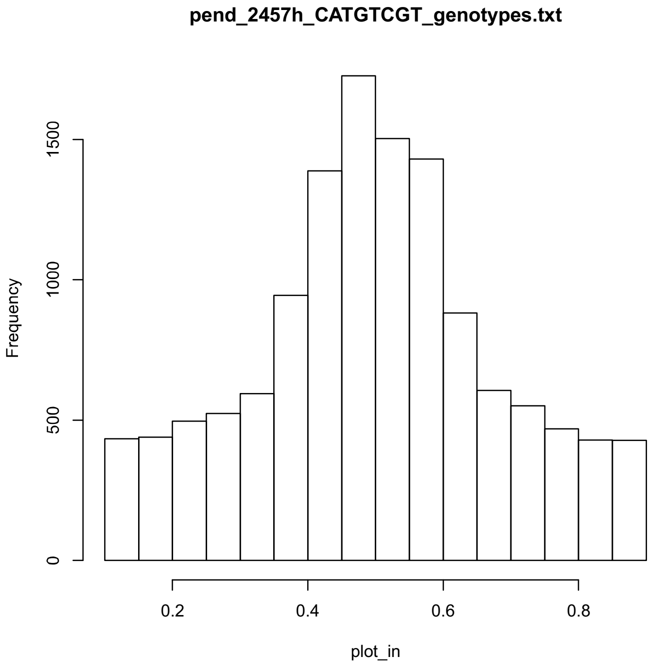
pend_2457h_CATGTCGT_genotypes.txt
Frequency
0
500
1000
1500
0.2
0.4
0.6
0.8
plot_in

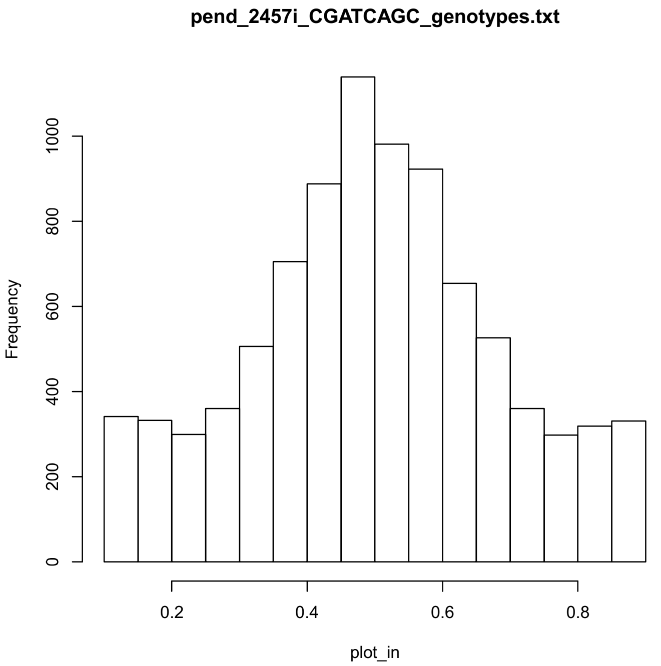
pend_2457i_CGATCAGC_genotypes.txt
Frequency
0
200
400
600
800
1000
0.2
0.4
0.6
0.8
plot_in

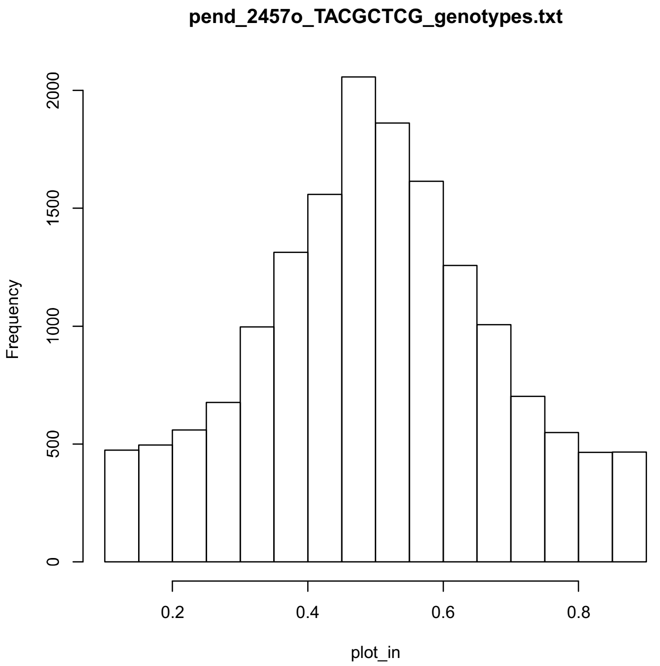
pend_2457o_TACGCTCG_genotypes.txt
Frequency
0
500
1000
1500
2000
0.2
0.4
0.6
0.8
plot_in

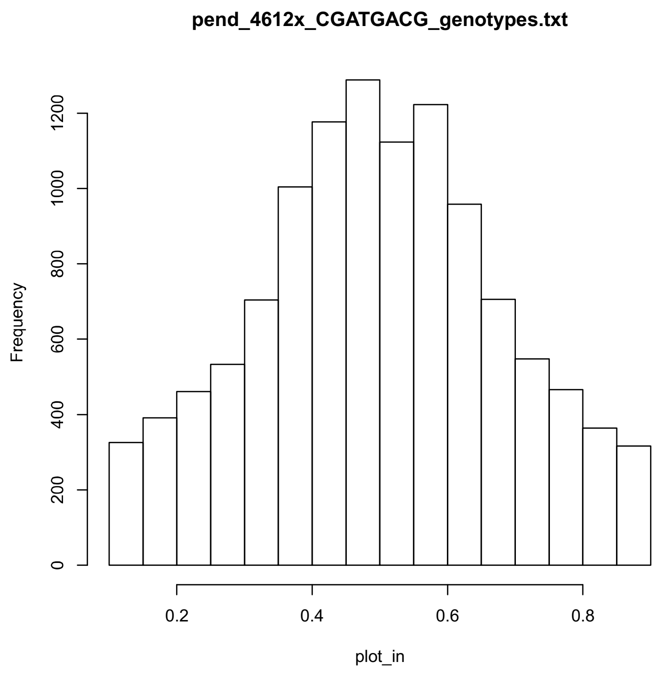
pend_4612x_CGATGACG_genotypes.txt
Frequency
plot_in

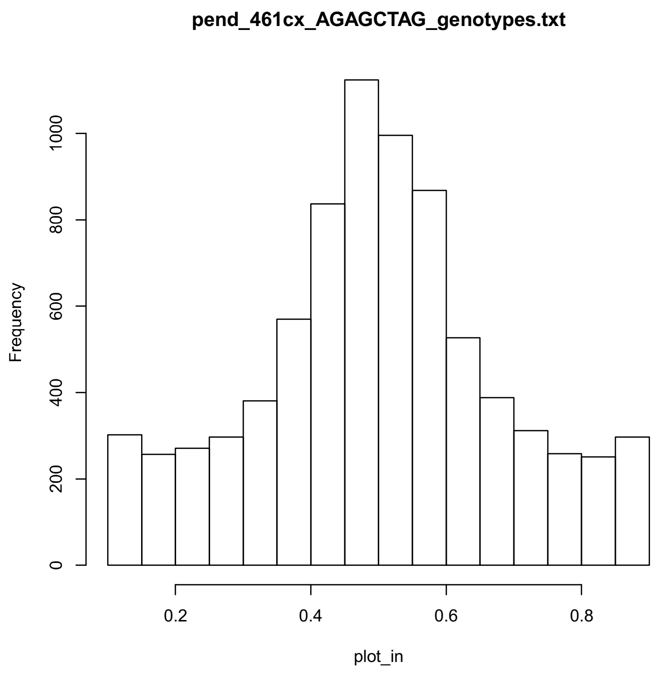
pend_461cx_AGAGCTAG_genotypes.txt
Frequency
plot_in

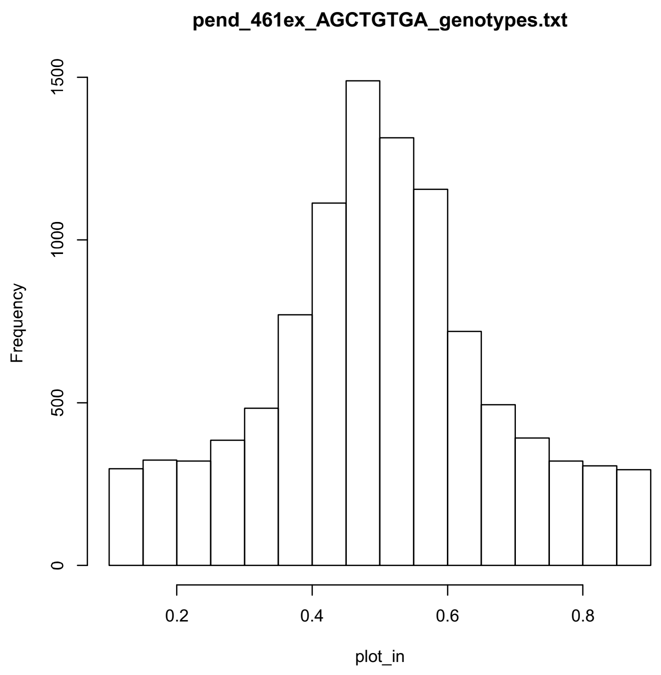
pend_461ex_AGCTGTGA_genotypes.txt
Frequency
plot_in

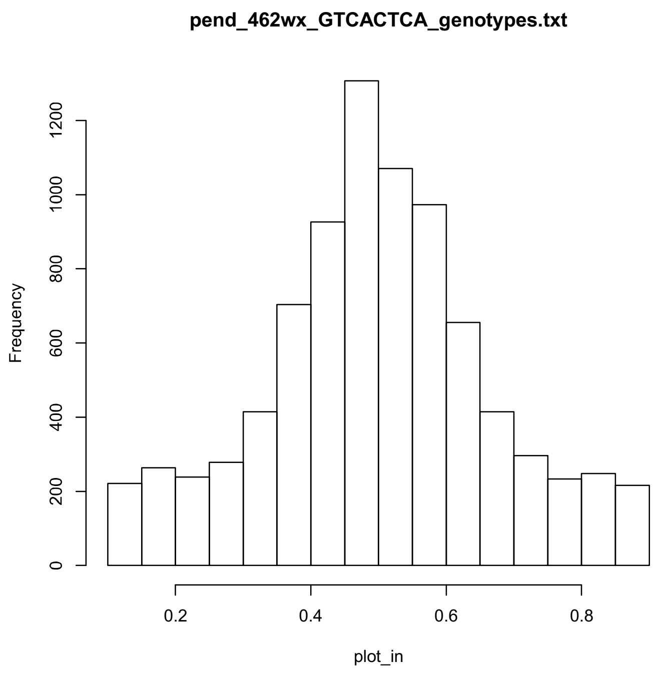
pend_462wx_GTCACTCA_genotypes.txt
Frequency
plot_in

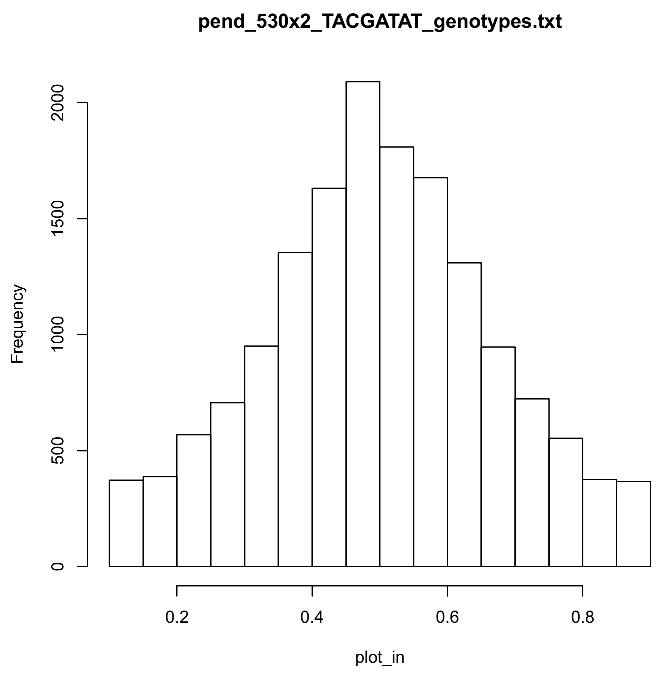
pend_530x2_TACGATAT_genotypes.txt
Frequency
plot_in

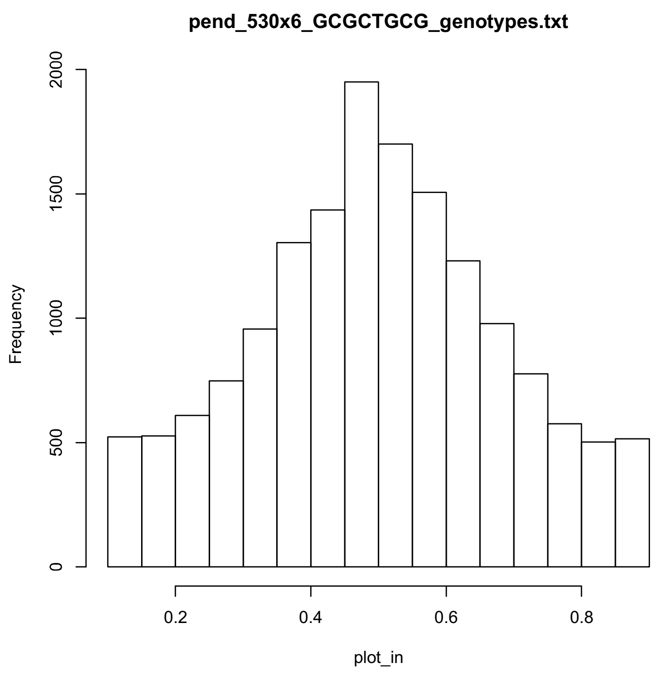
pend_530x6_GCGCTGCG_genotypes.txt
Frequency
plot_in

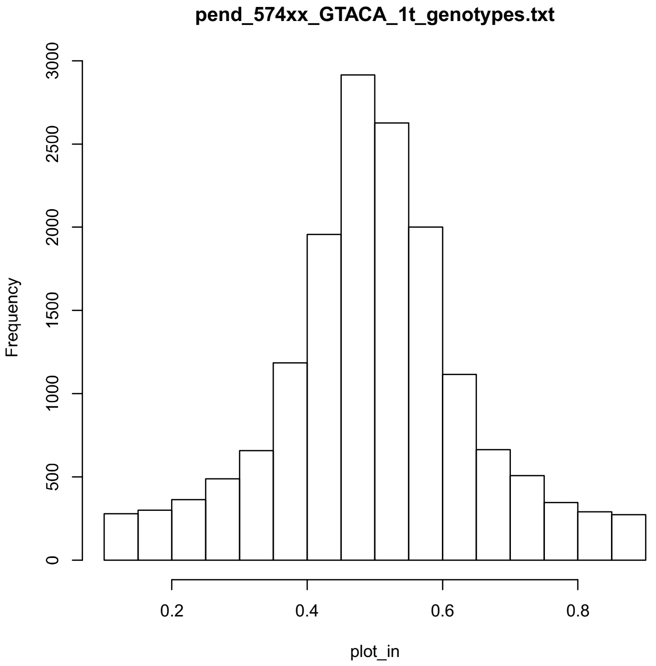
pend_574xx_GTACA_1t_genotypes.txt
Frequency
0
500
1000
1500
2000
2500
3000
0.2
0.4
0.6
0.8
plot_in

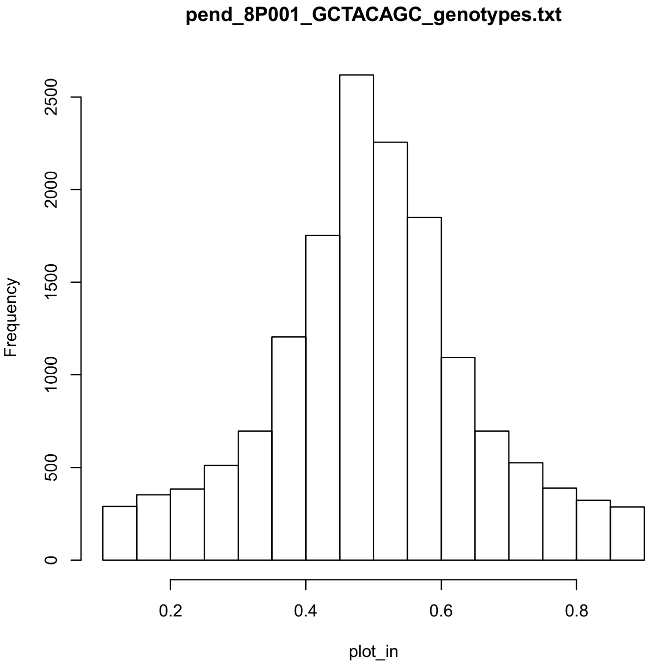
pend_8P001_GCTACAGC_genotypes.txt
Frequency
0
500
1000
1500
2000
2500
0.2
0.4
0.6
0.8
plot_in

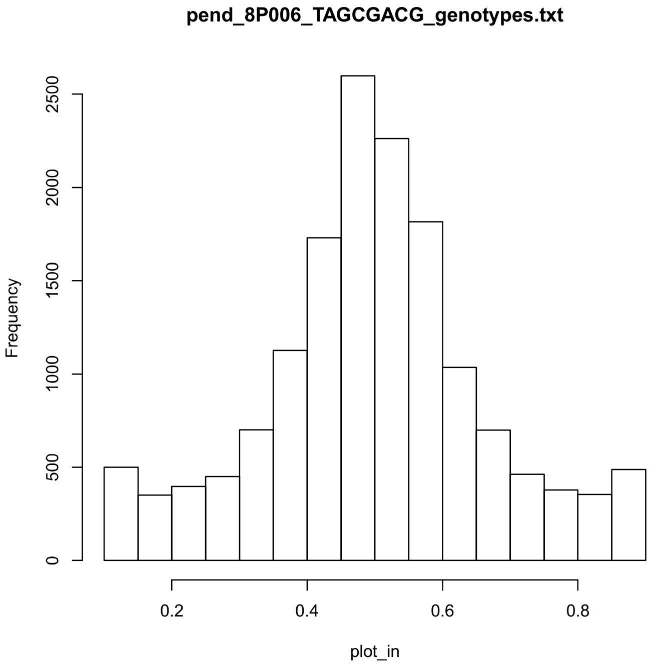
pend_8P006_TAGCGACG_genotypes.txt
Frequency
0
500
1000
1500
2000
2500
0.2
0.4
0.6
0.8
plot_in

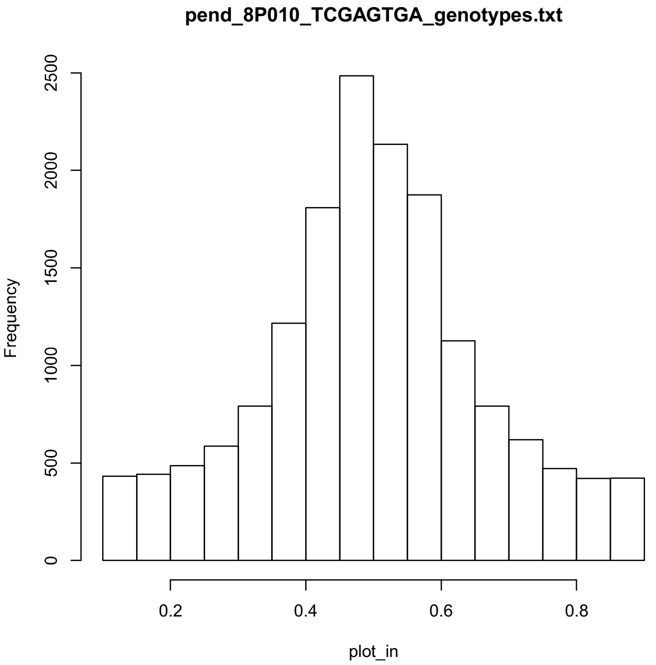
pend_8P010_TCGAGTGA_genotypes.txt
Frequency
0
500
1000
1500
2000
2500
0.2
0.4
0.6
0.8
plot_in

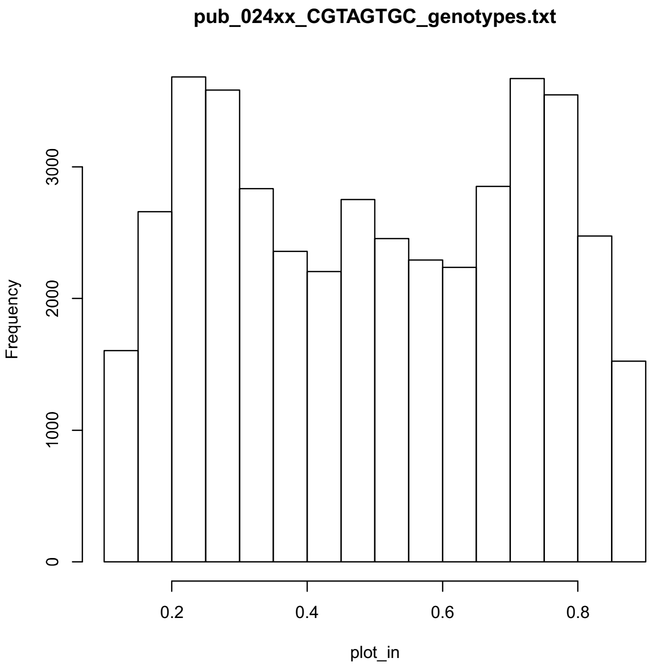
pub_024xx_CGTAGTGC_genotypes.txt
Frequency
0
1000
2000
3000
0.2
0.4
0.6
0.8
plot_in

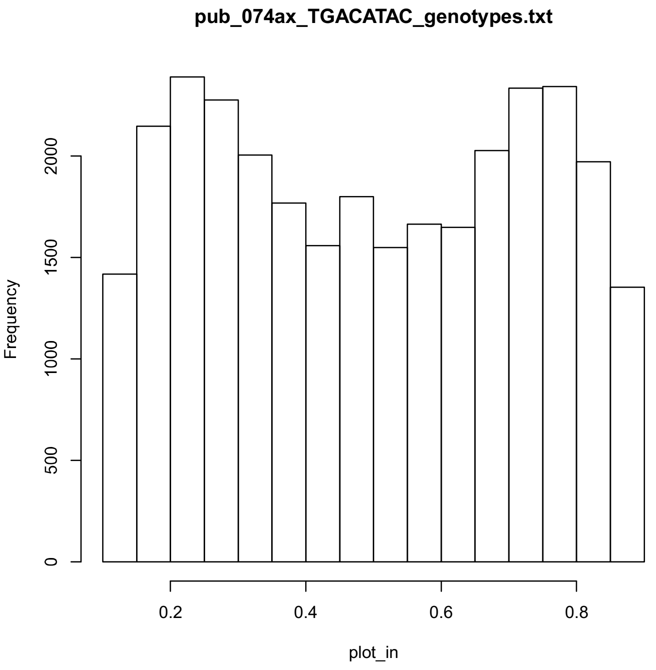
pub_074ax_TGACATAC_genotypes.txt
Frequency
0
500
1000
1500
2000
0.2
0.4
0.6
0.8
plot_in

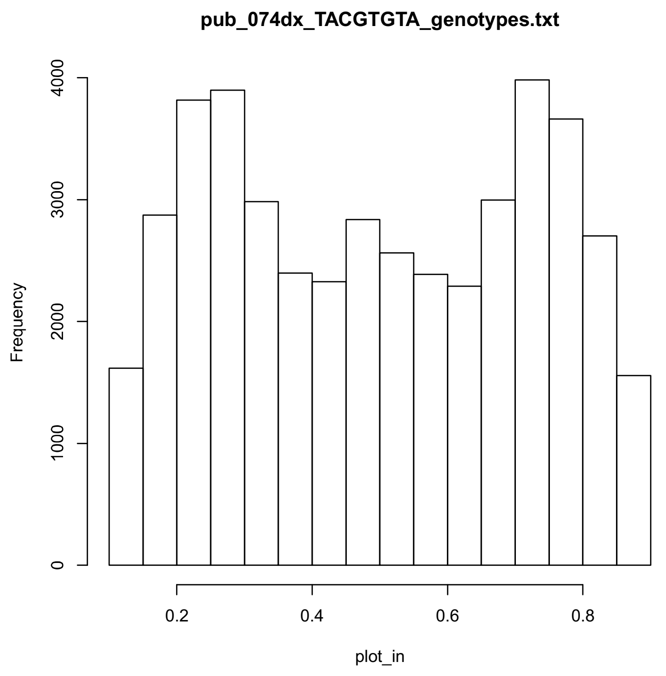
pub_074dx_TACGTGTA_genotypes.txt
Frequency
0
1000
2000
3000
4000
0.2
0.4
0.6
0.8
plot_in

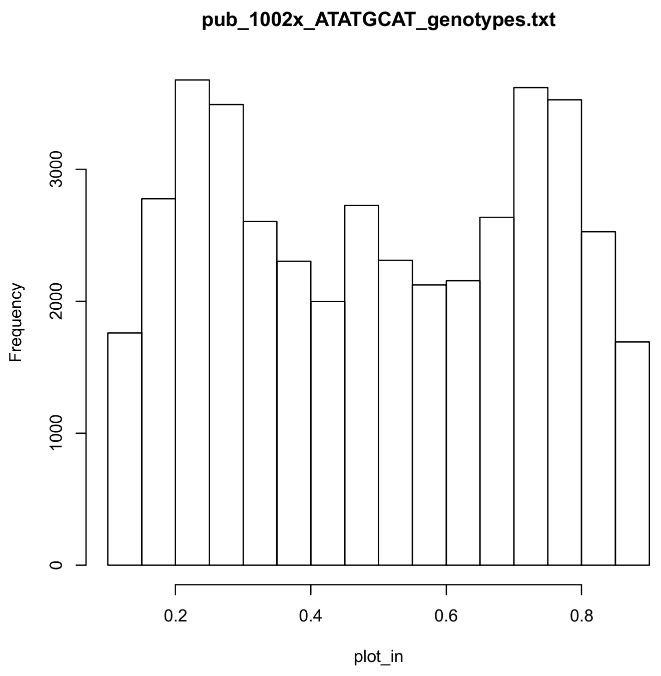
pub_1002x_ATATGCAT_genotypes.txt
Frequency
0
1000
2000
3000
0.2
0.4
0.6
0.8
plot_in

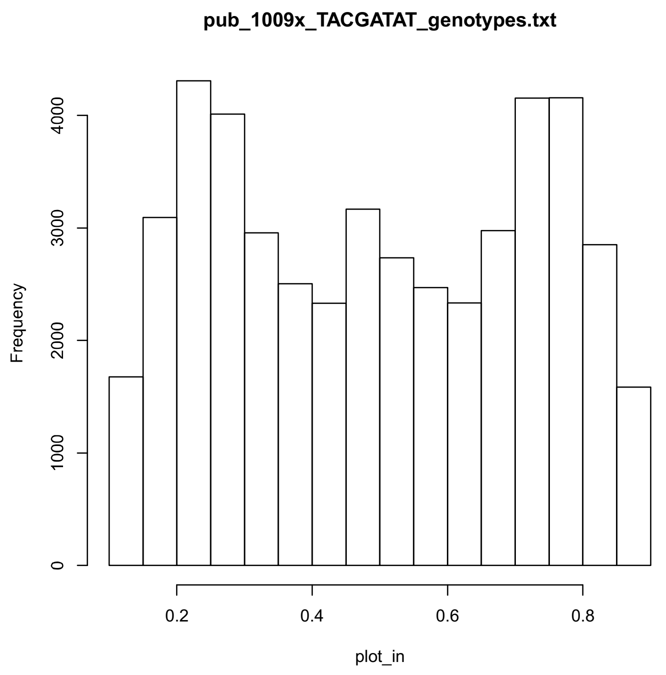
pub_1009x_TACGATAT_genotypes.txt
Frequency
0
1000
2000
3000
4000
0.2
0.4
0.6
0.8
plot_in

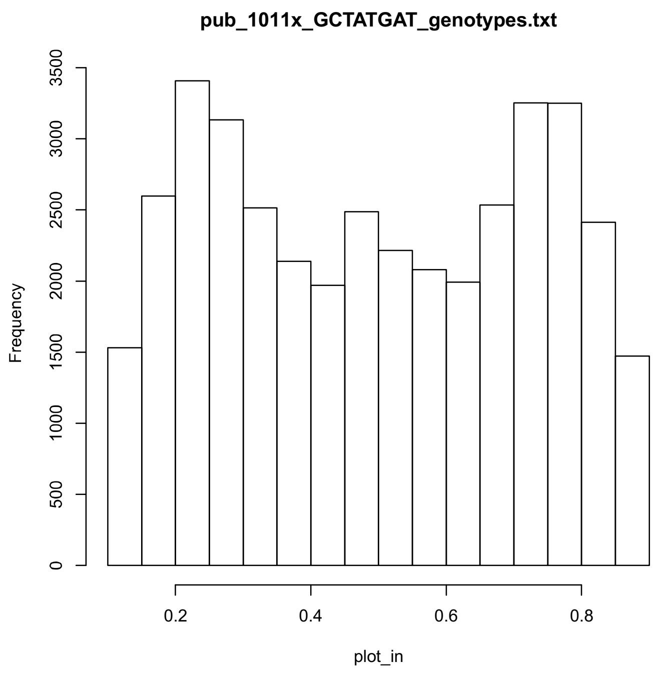
pub_1011x_GCTATGAT_genotypes.txt
Frequency
0
500
1000
1500
2000
2500
3000
3500
0.2
0.4
0.6
0.8
plot_in

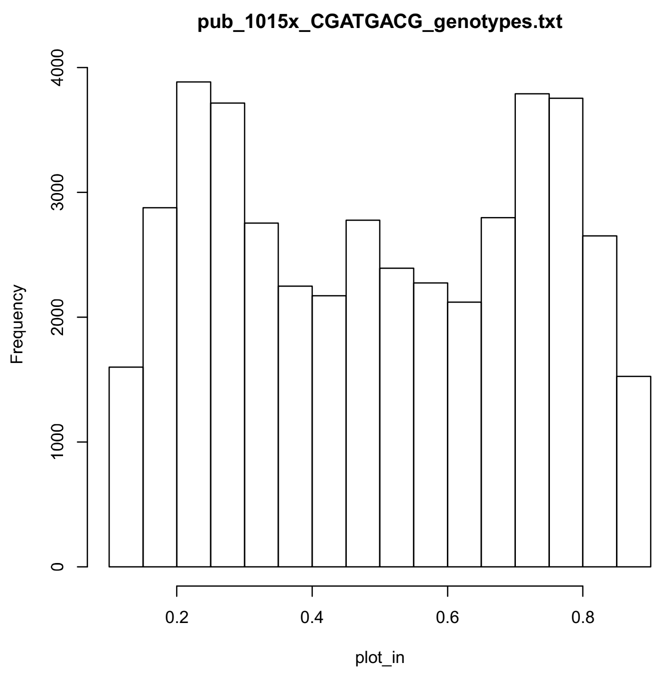
pub_1015x_CGATGACG_genotypes.txt
Frequency
0
1000
2000
3000
4000
0.2
0.4
0.6
0.8
plot_in

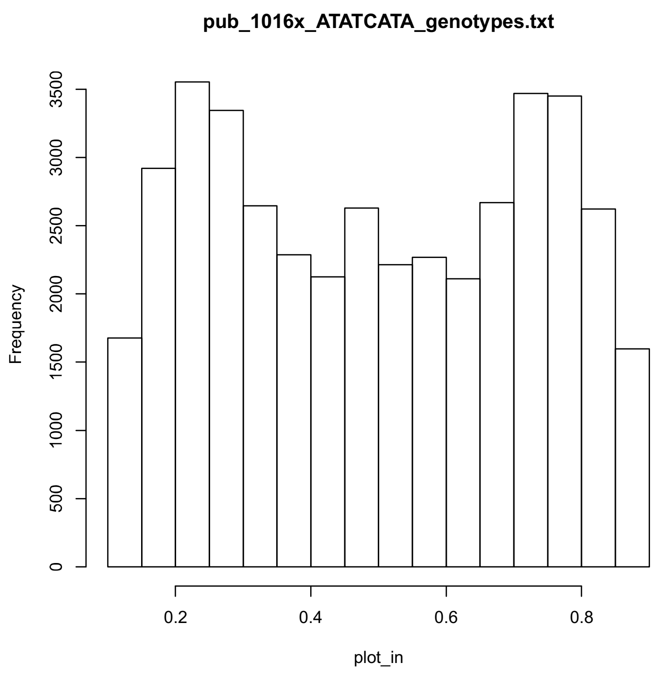
pub_1016x_ATATCATA_genotypes.txt
Frequency
0
500
1000
1500
2000
2500
3000
3500
0.2
0.4
0.6
0.8
plot_in

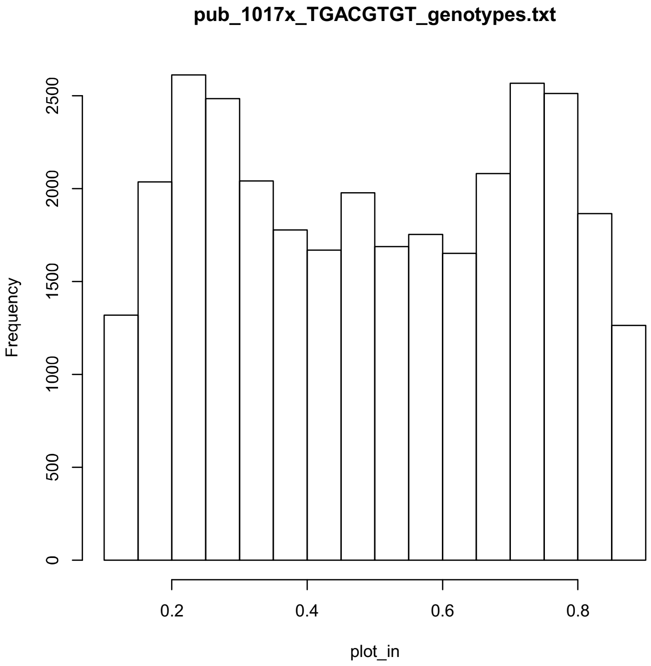
pub_1017x_TGACGTGT_genotypes.txt
Frequency
0
500
1000
1500
2000
2500
0.2
0.4
0.6
0.8
plot_in

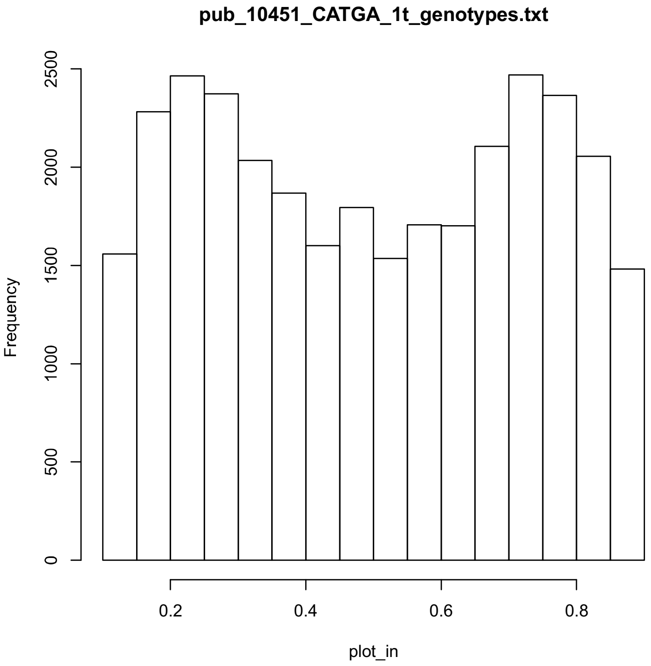
pub_10451_CATGA_1t_genotypes.txt
Frequency
0
500
1000
1500
2000
2500
0.2
0.4
0.6
0.8
plot_in

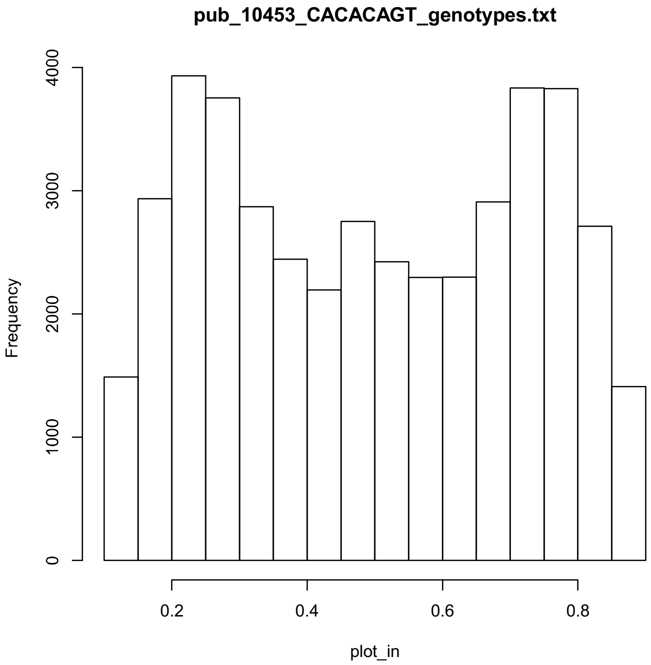
pub_10453_CACACAGT_genotypes.txt
Frequency
0
1000
2000
3000
4000
0.2
0.4
0.6
0.8
plot_in

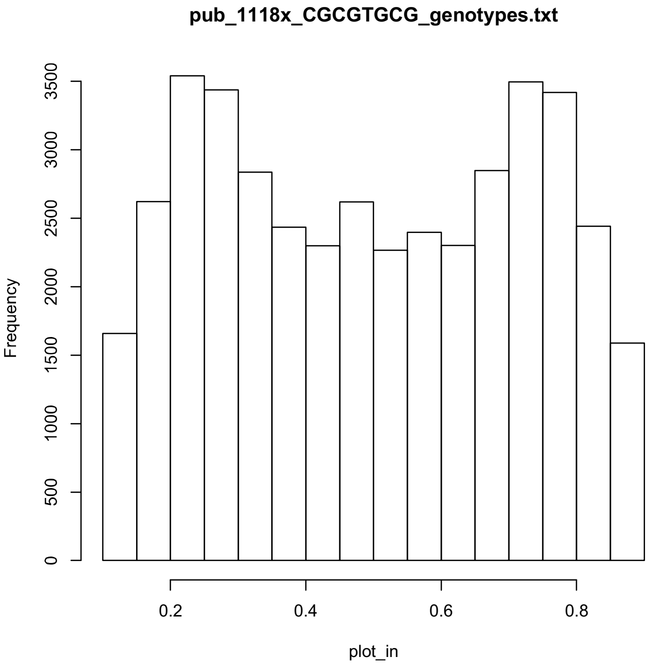
pub_1118x_CGCGTGCG_genotypes.txt
Frequency
0
500
1000
1500
2000
2500
3000
3500
0.2
0.4
0.6
0.8
plot_in

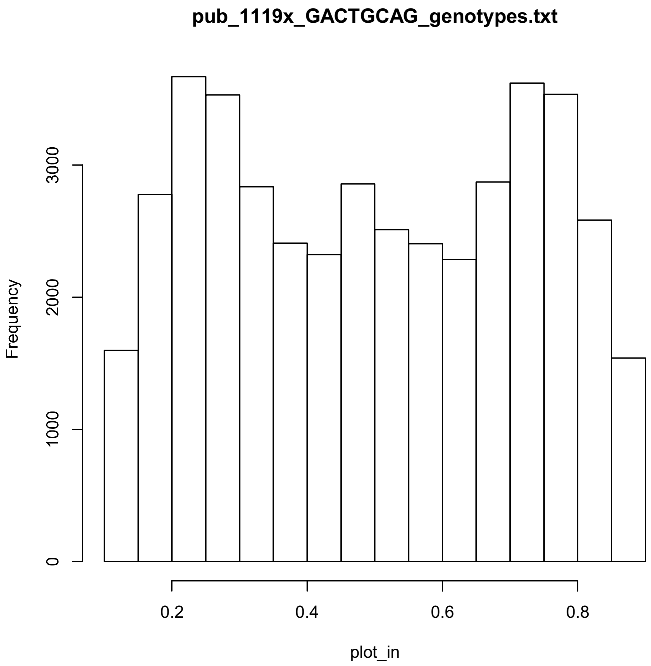
pub_1119x_GACTGCAG_genotypes.txt
Frequency
0
1000
2000
3000
0.2
0.4
0.6
0.8
plot_in

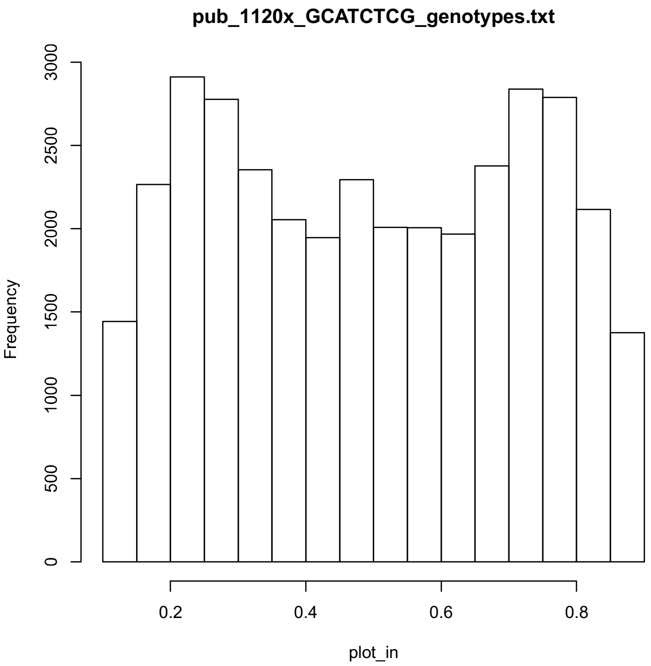
pub_1120x_GCATCTCG_genotypes.txt
Frequency
0
500
1000
1500
2000
2500
3000
0.2
0.4
0.6
0.8
plot_in

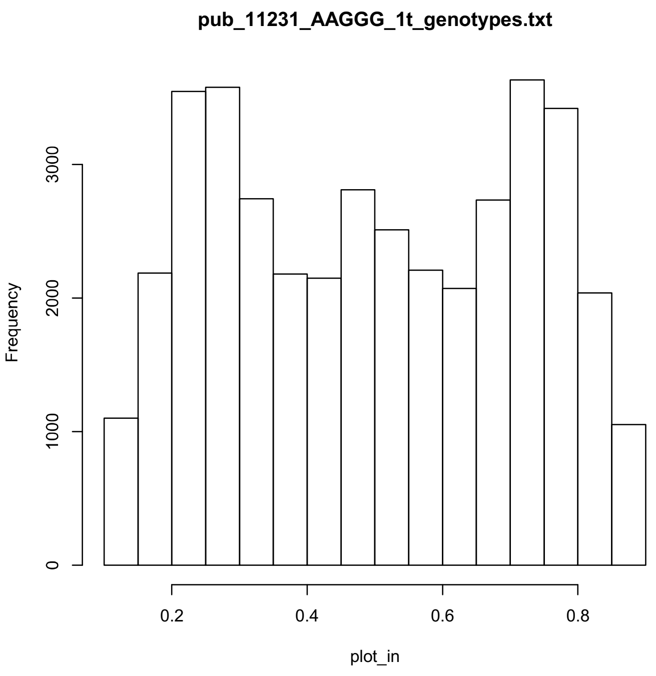
pub_11231_AAGGG_1t_genotypes.txt
Frequency
0
1000
2000
3000
0.2
0.4
0.6
0.8
plot_in

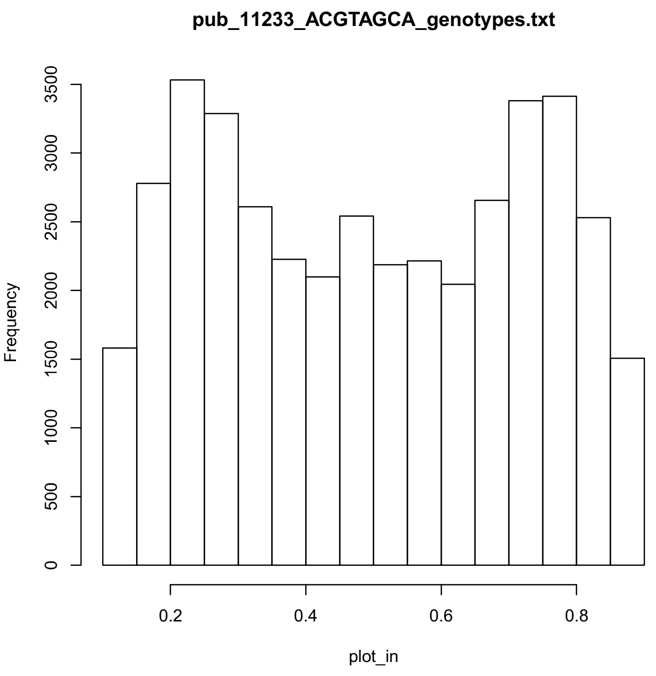
pub_11233_ACGTAGCA_genotypes.txt
Frequency
0
500
1000
1500
2000
2500
3000
3500
0.2
0.4
0.6
0.8
plot_in

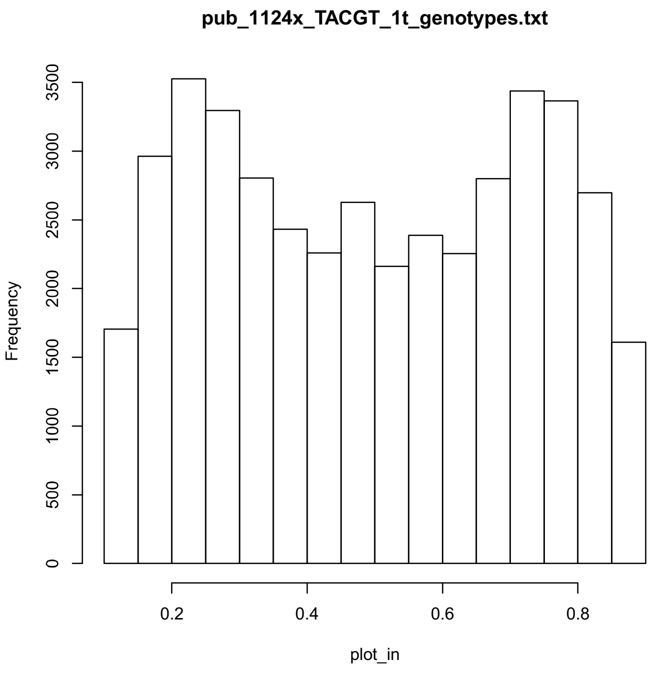
pub_1124x_TACGT_1t_genotypes.txt
Frequency
0
500
1000
1500
2000
2500
3000
3500
0.2
0.4
0.6
0.8
plot_in

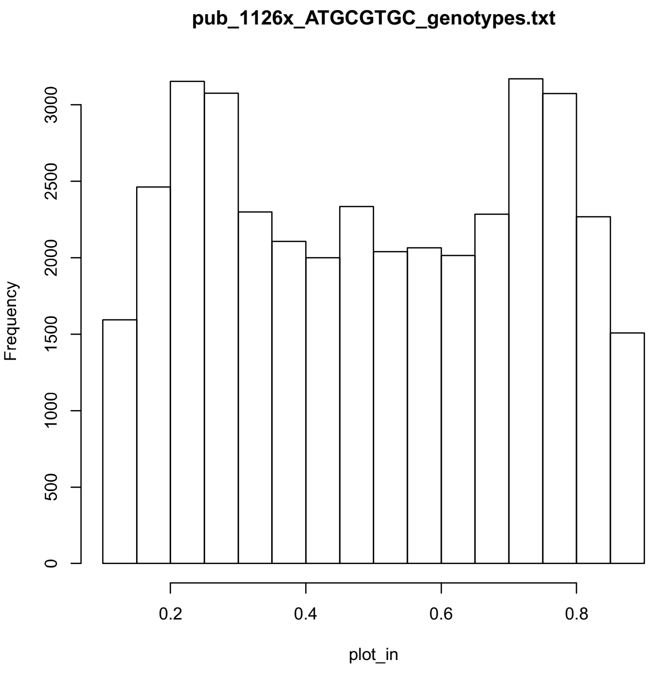
pub_1126x_ATGCGTGC_genotypes.txt
Frequency
0
500
1000
1500
2000
2500
3000
0.2
0.4
0.6
0.8
plot_in

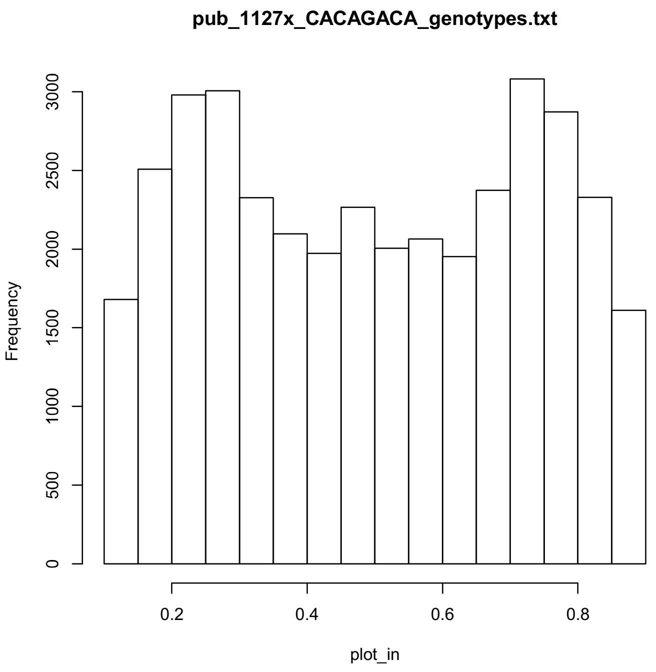
pub_1127x_CACAGACA_genotypes.txt
Frequency
0
500
1000
1500
2000
2500
3000
0.2
0.4
0.6
0.8
plot_in

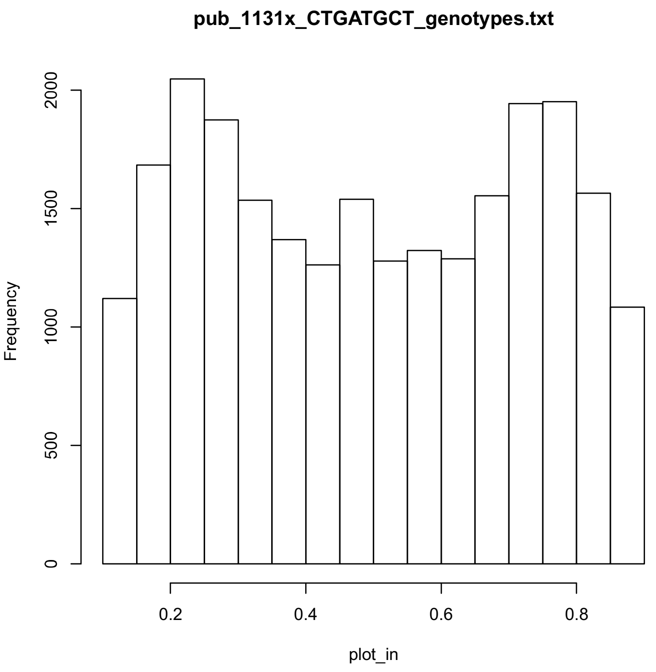
pub_1131x_CTGATGCT_genotypes.txt
Frequency
0
500
1000
1500
2000
0.2
0.4
0.6
0.8
plot_in

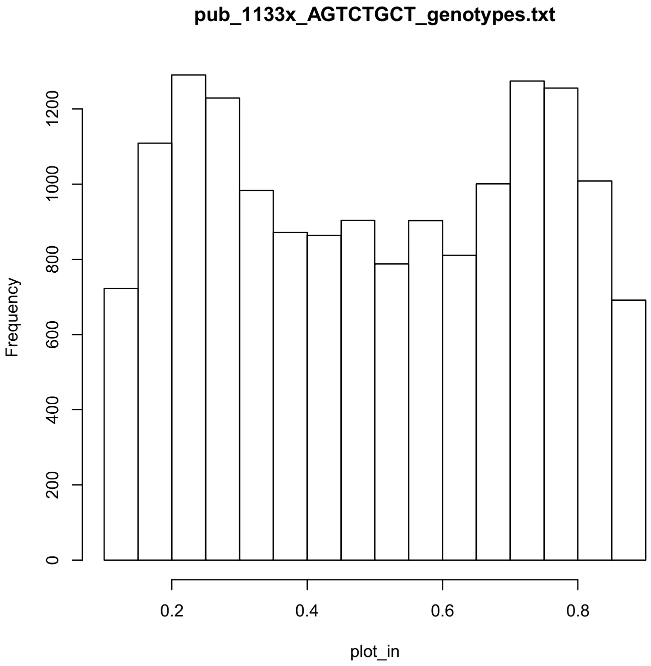
pub_1133x_AGTCTGCT_genotypes.txt
Frequency
0
200
400
600
800
1000
1200
0.2
0.4
0.6
0.8
plot_in

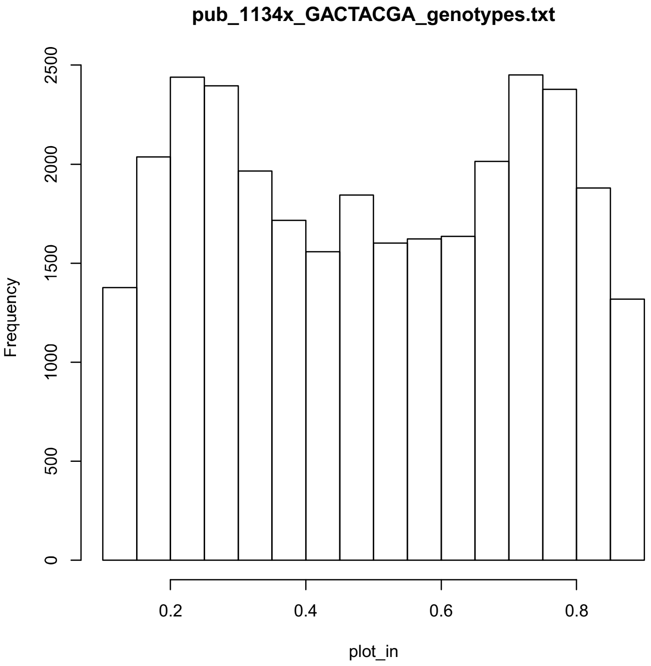
pub_1134x_GACTACGA_genotypes.txt
Frequency
0
500
1000
1500
2000
2500
0.2
0.4
0.6
0.8
plot_in

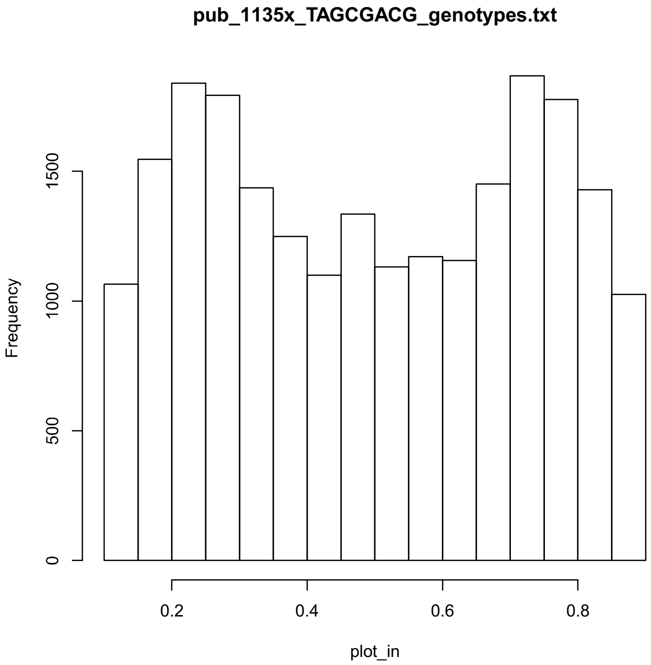
pub_1135x_TAGCGACG_genotypes.txt
Frequency
0
500
1000
1500
0.2
0.4
0.6
0.8
plot_in

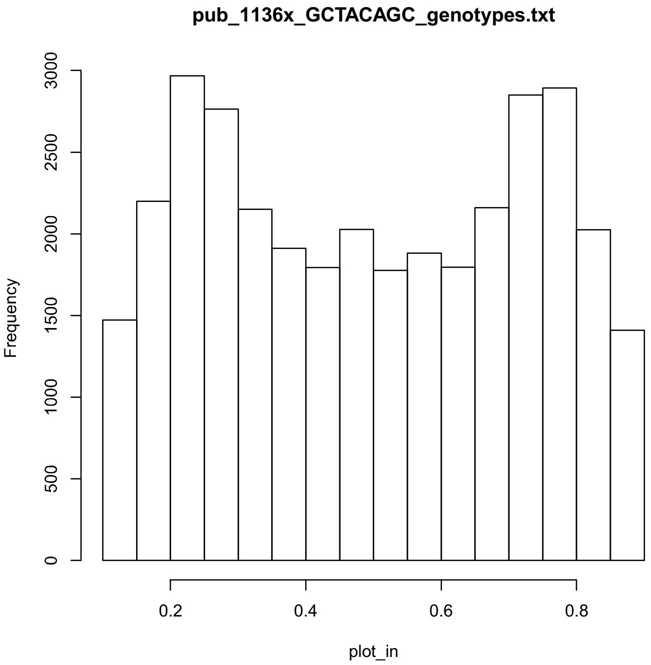
pub_1136x_GCTACAGC_genotypes.txt
Frequency
0
500
1000
1500
2000
2500
3000
0.2
0.4
0.6
0.8
plot_in

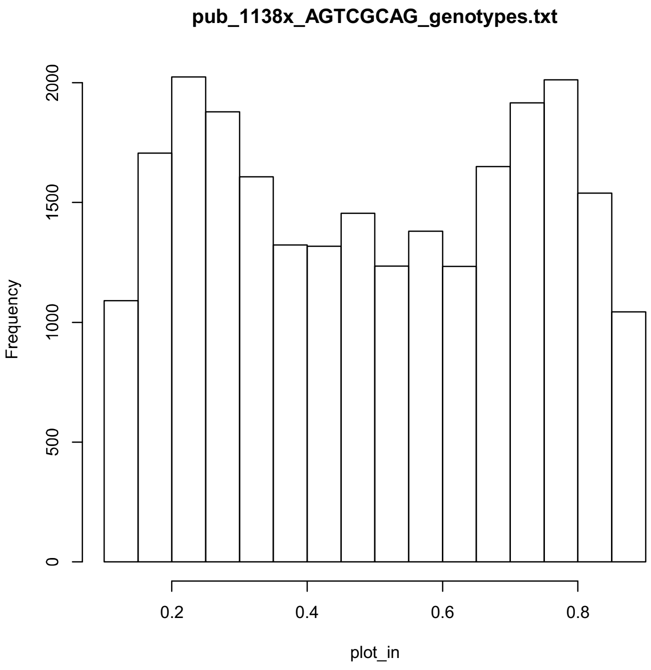
pub_1138x_AGTCGCAG_genotypes.txt
Frequency
0
500
1000
1500
2000
0.2
0.4
0.6
0.8
plot_in

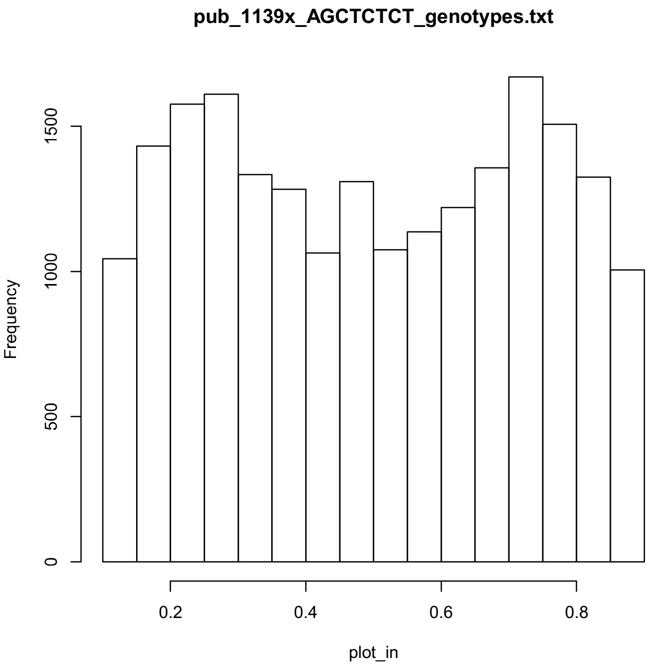
pub_1139x_AGCTCTCT_genotypes.txt
Frequency
0
500
1000
1500
0.2
0.4
0.6
0.8
plot_in

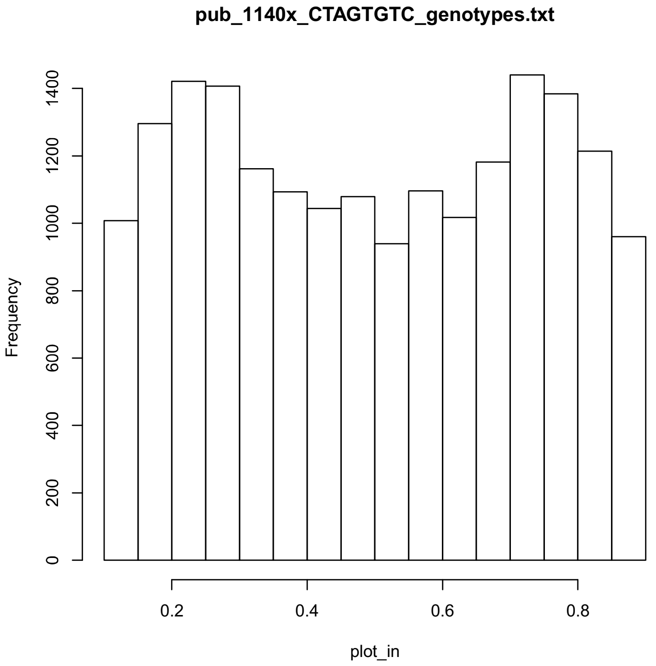
pub_1140x_CTAGTGTC_genotypes.txt
Frequency
0
200
400
600
800
1000
1200
1400
0.2
0.4
0.6
0.8
plot_in

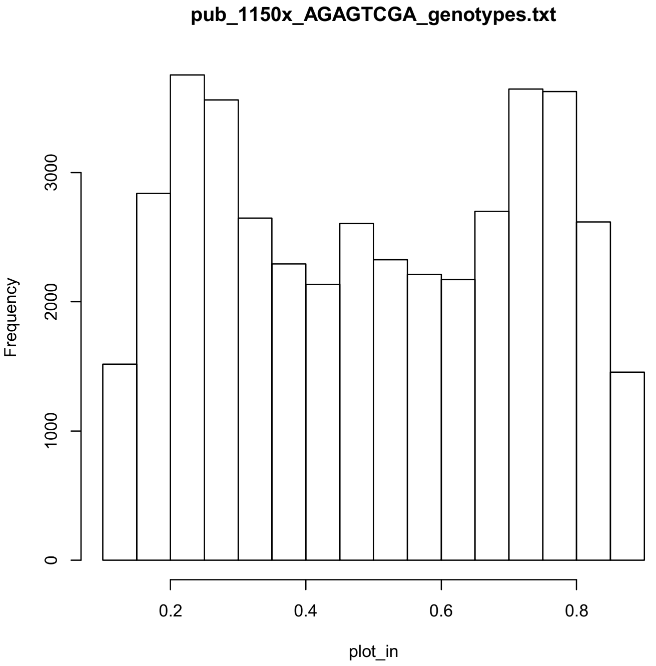
pub_1150x_AGAGTCGA_genotypes.txt
Frequency
0
1000
2000
3000
0.2
0.4
0.6
0.8
plot_in

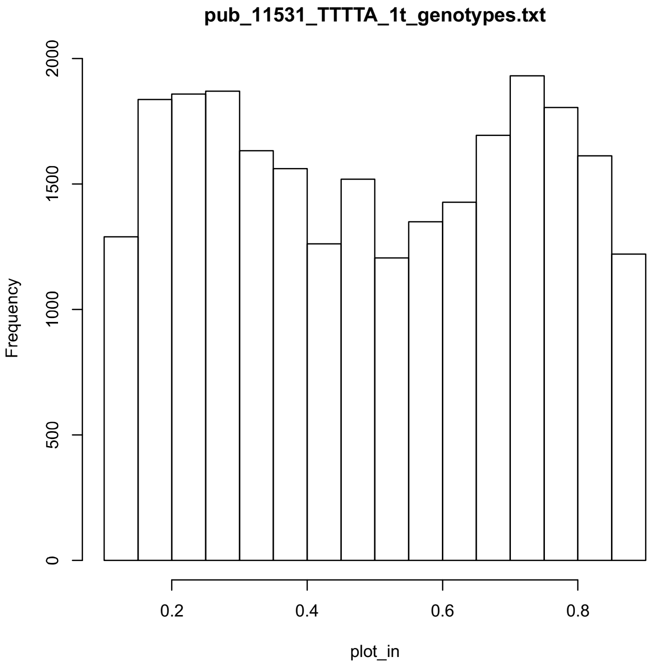
pub_11531_TTTTA_1t_genotypes.txt
Frequency
0
500
1000
1500
2000
0.2
0.4
0.6
0.8
plot_in

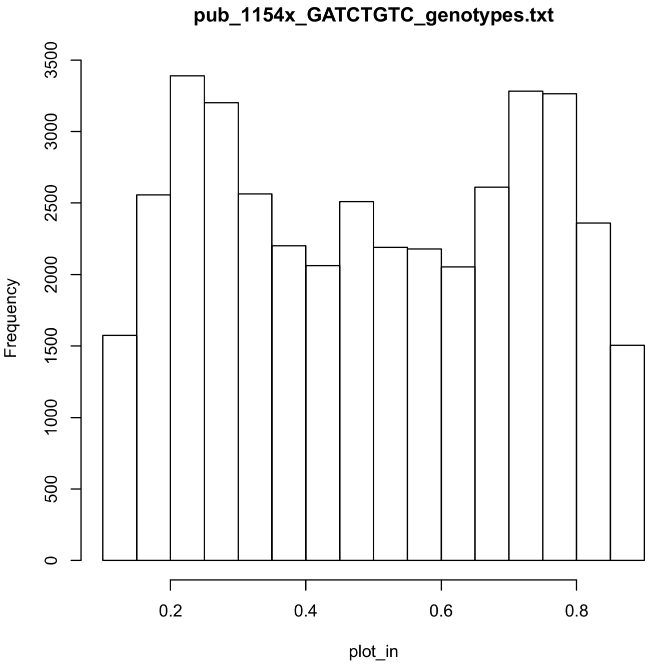
pub_1154x_GATCTGTC_genotypes.txt
Frequency
0
500
1000
1500
2000
2500
3000
3500
0.2
0.4
0.6
0.8
plot_in

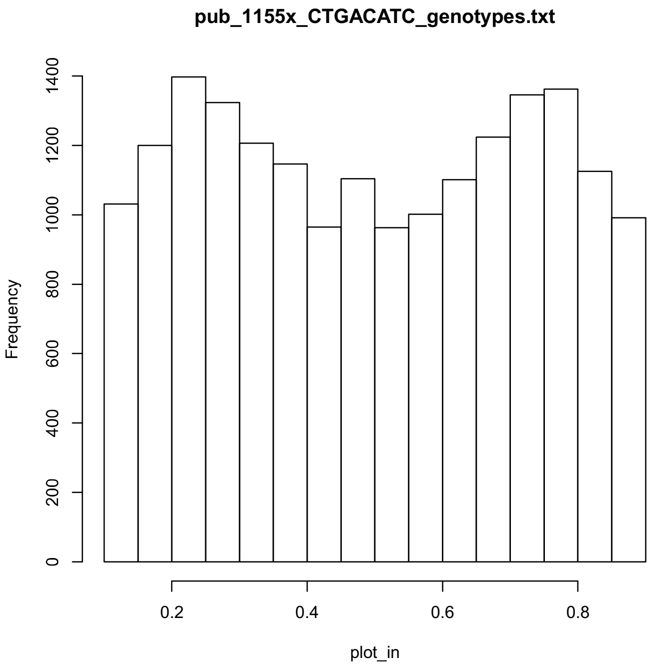
pub_1155x_CTGACATC_genotypes.txt
Frequency
0
200
400
600
800
1000
1200
1400
0.2
0.4
0.6
0.8
plot_in

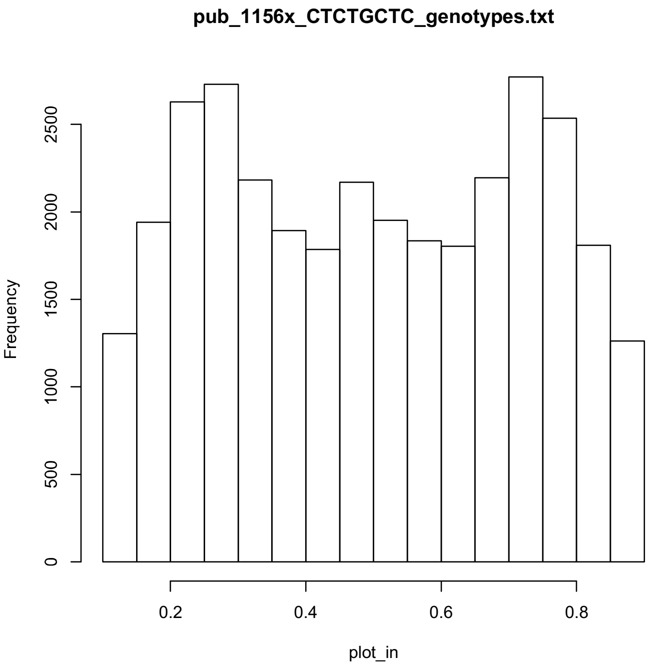
pub_1156x_CTCTGCTC_genotypes.txt
Frequency
0
500
1000
1500
2000
2500
0.2
0.4
0.6
0.8
plot_in

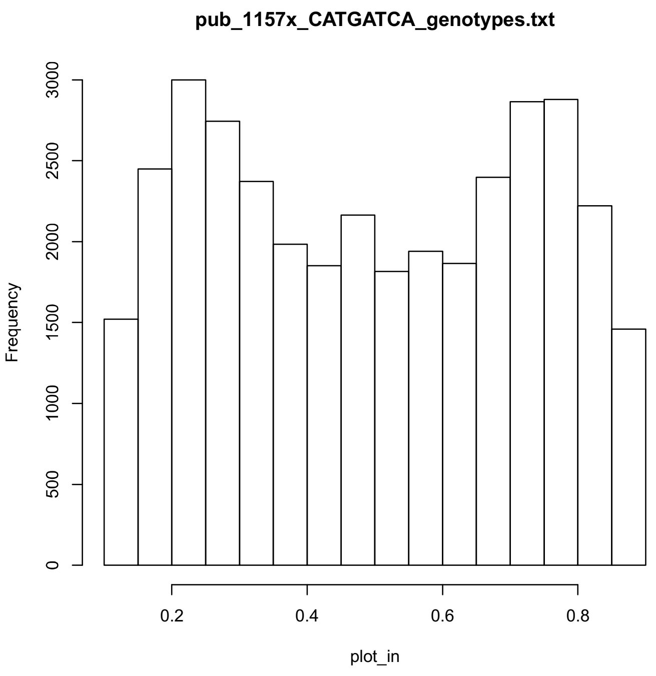
pub_1157x_CATGATCA_genotypes.txt
Frequency
0
500
1000
1500
2000
2500
3000
0.2
0.4
0.6
0.8
plot_in

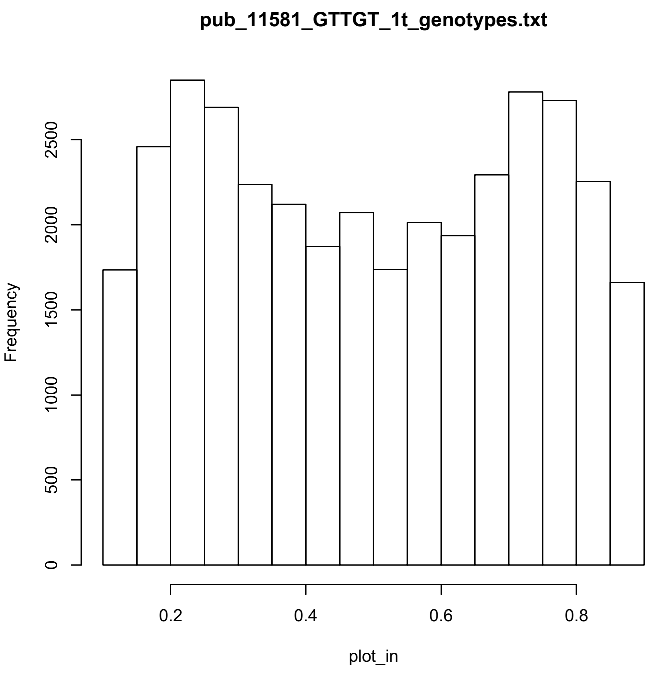
pub_11581_GTTGT_1t_genotypes.txt
Frequency
0
500
1000
1500
2000
2500
0.2
0.4
0.6
0.8
plot_in

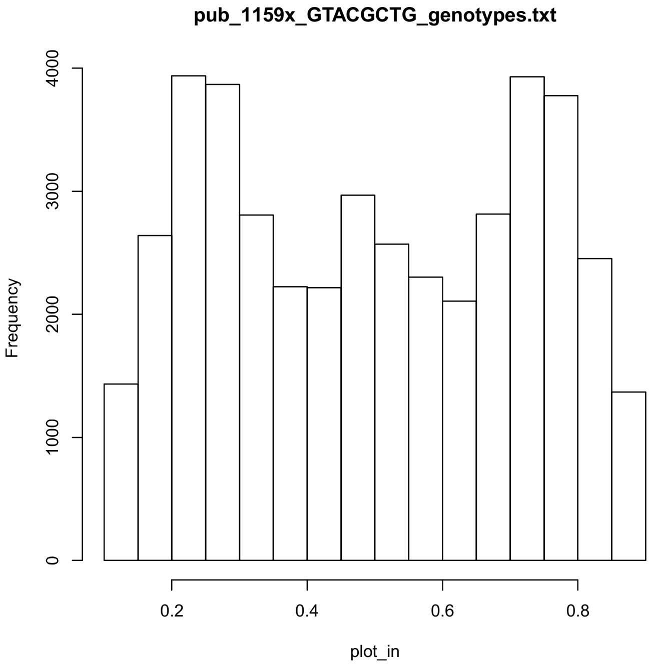
pub_1159x_GTACGCTG_genotypes.txt
Frequency
0
1000
2000
3000
4000
0.2
0.4
0.6
0.8
plot_in

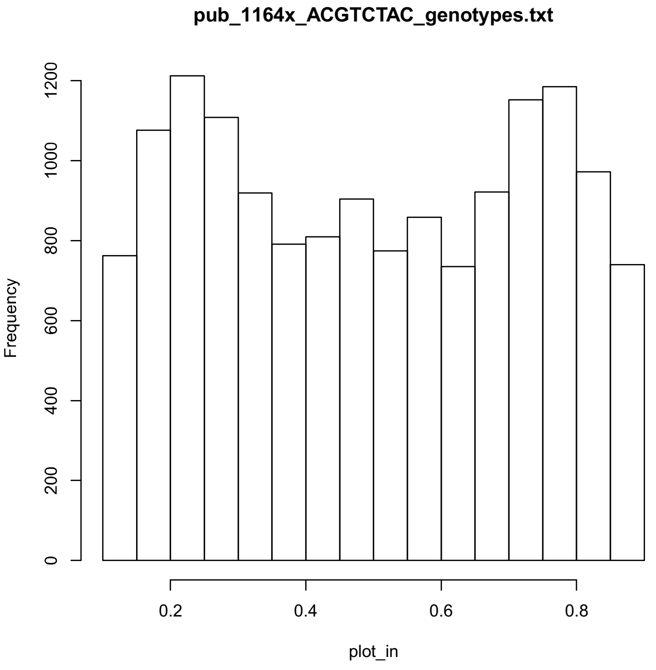
pub_1164x_ACGTCTAC_genotypes.txt
Frequency
0
200
400
600
800
1000
1200
0.2
0.4
0.6
0.8
plot_in

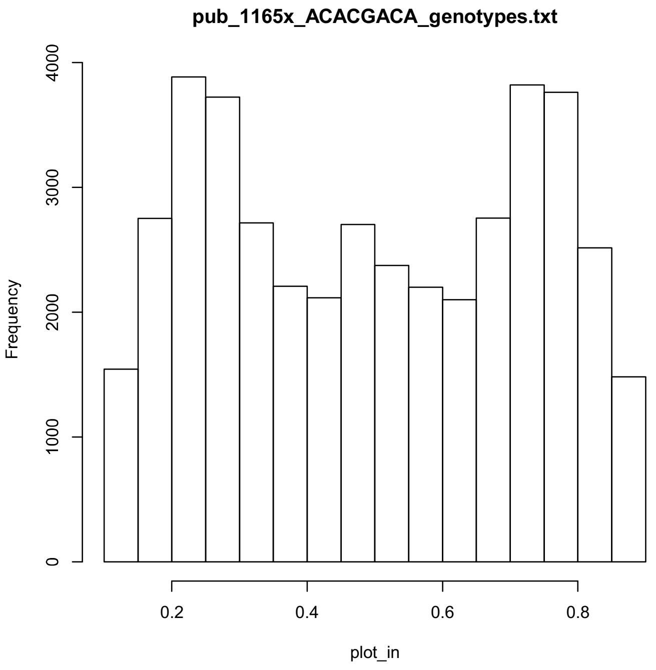
pub_1165x_ACACGACA_genotypes.txt
Frequency
0
1000
2000
3000
4000
0.2
0.4
0.6
0.8
plot_in

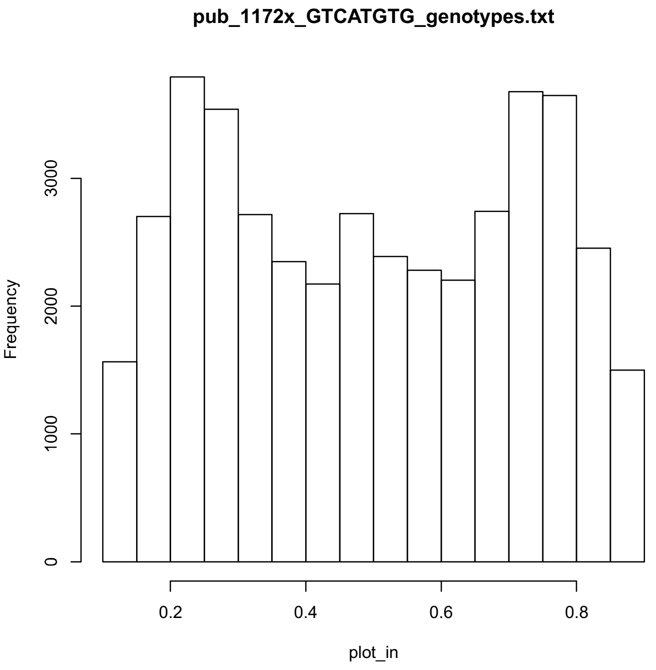
pub_1172x_GTCATGTG_genotypes.txt
Frequency
0
1000
2000
3000
0.2
0.4
0.6
0.8
plot_in

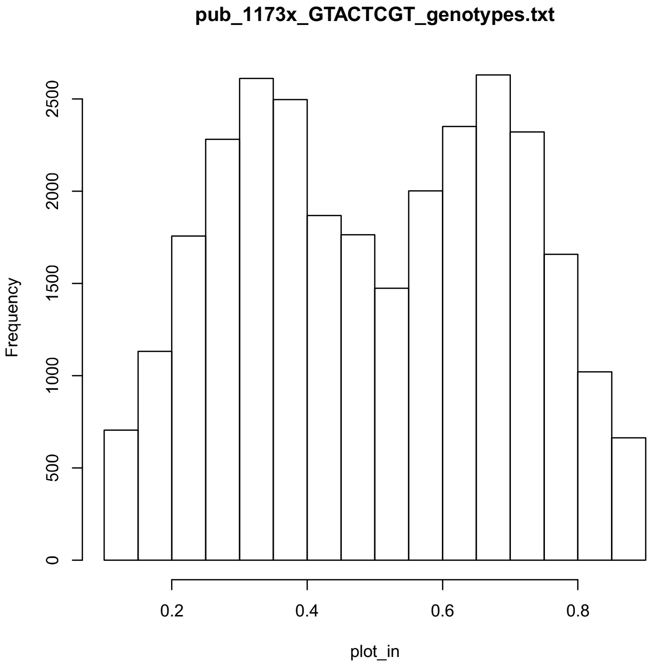
pub_1173x_GTACTCGT_genotypes.txt
Frequency
0
500
1000
1500
2000
2500
0.2
0.4
0.6
0.8
plot_in

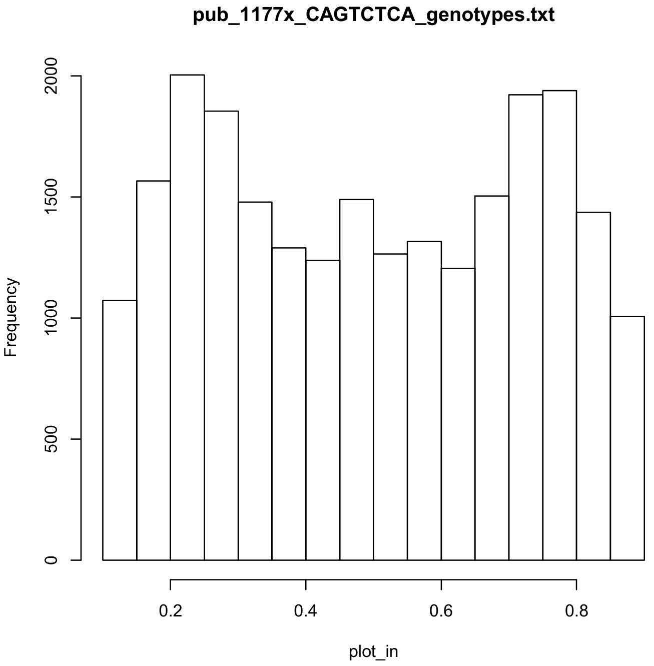
pub_1177x_CAGTCTCA_genotypes.txt
Frequency
0
500
1000
1500
2000
0.2
0.4
0.6
0.8
plot_in

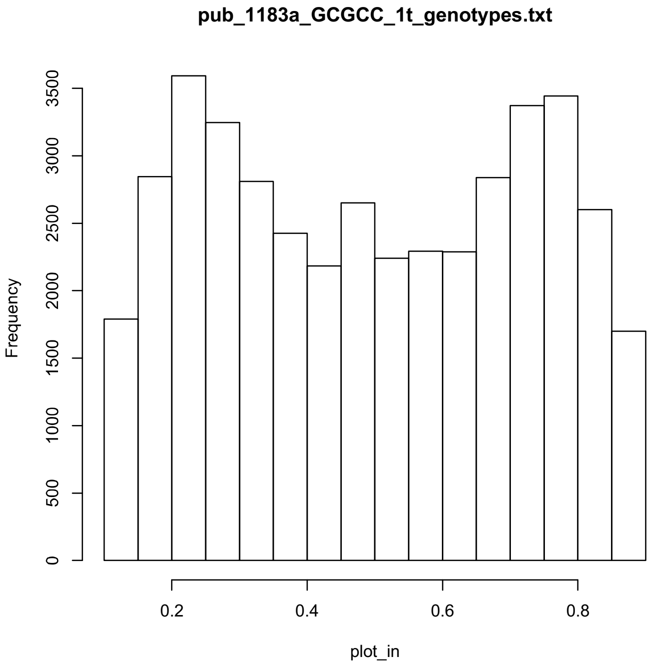
pub_1183a_GCGCC_1t_genotypes.txt
Frequency
0
500
1000
1500
2000
2500
3000
3500
0.2
0.4
0.6
0.8
plot_in

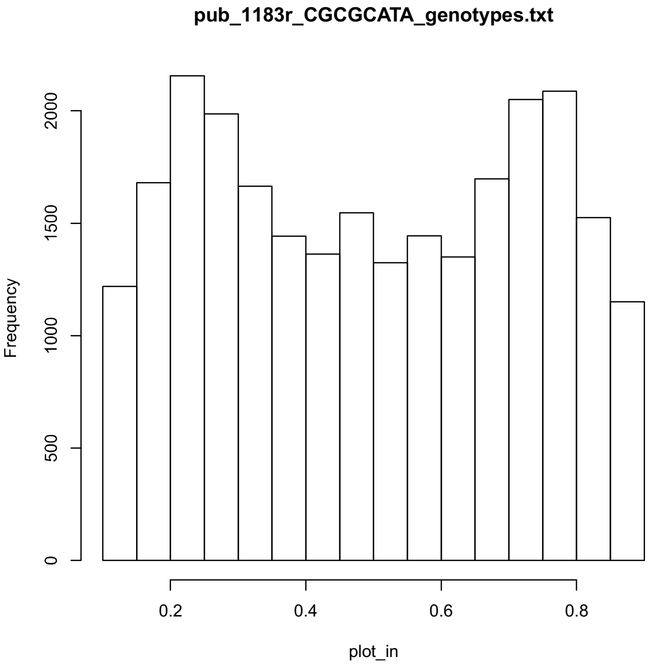
pub_1183r_CGCGCATA_genotypes.txt
Frequency
0
500
1000
1500
2000
0.2
0.4
0.6
0.8
plot_in

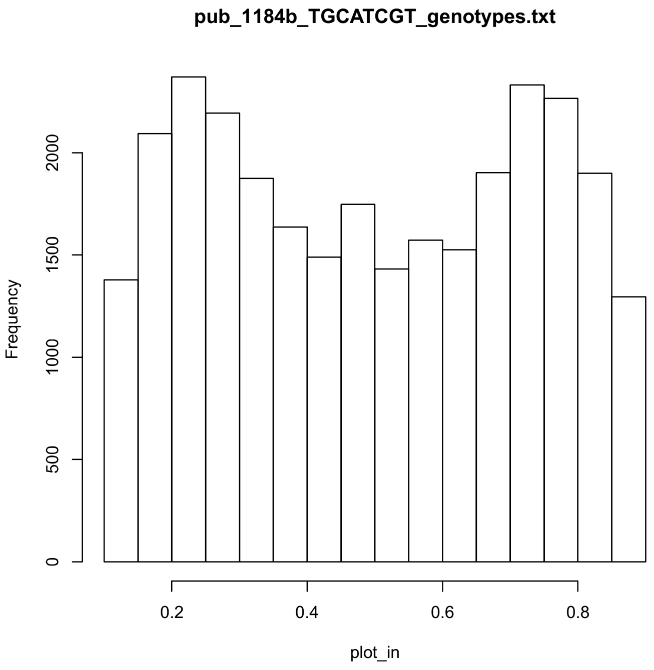
pub_1184b_TGCATCGT_genotypes.txt
Frequency
0
500
1000
1500
2000
0.2
0.4
0.6
0.8
plot_in

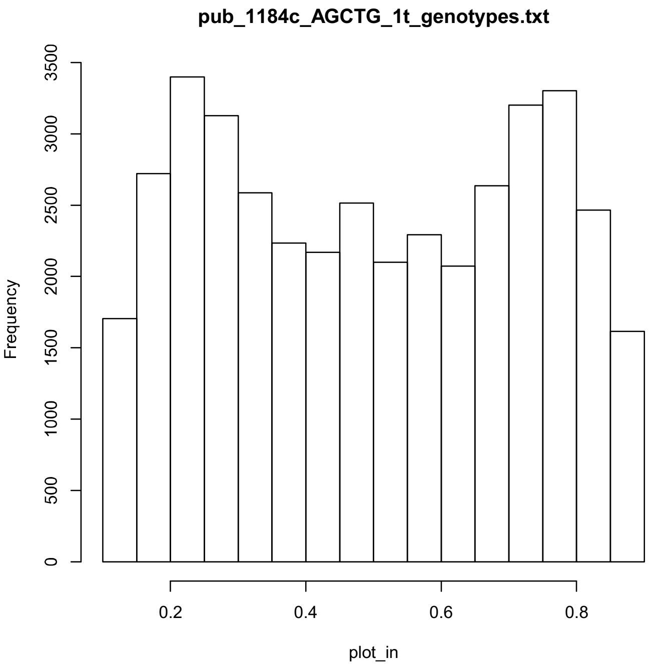
pub_1184c_AGCTG_1t_genotypes.txt
Frequency
0
500
1000
1500
2000
2500
3000
3500
0.2
0.4
0.6
0.8
plot_in

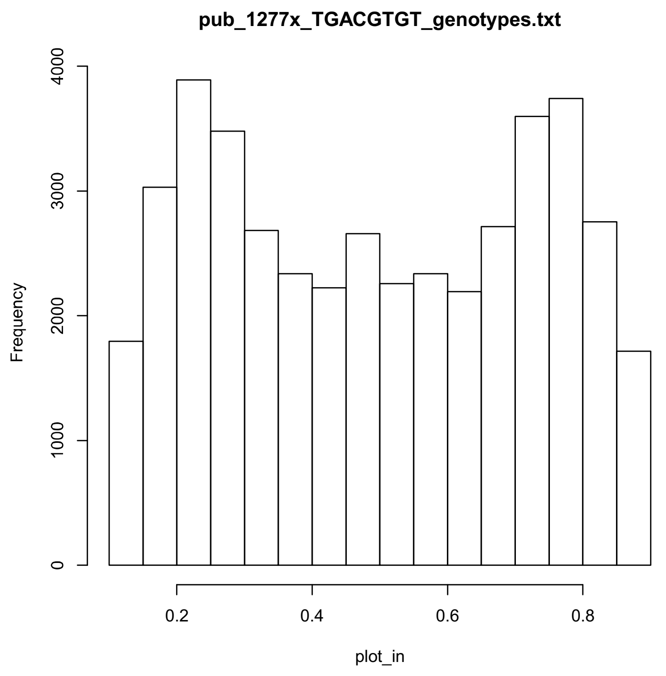
pub_1277x_TGACGTGT_genotypes.txt
Frequency
0
1000
2000
3000
4000
0.2
0.4
0.6
0.8
plot_in

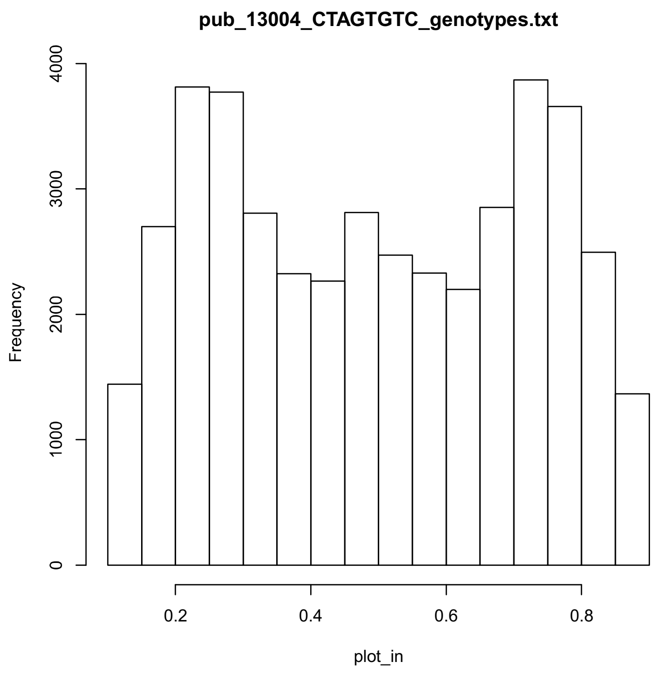
pub_13004_CTAGTGTC_genotypes.txt
Frequency
0
1000
2000
3000
4000
0.2
0.4
0.6
0.8
plot_in

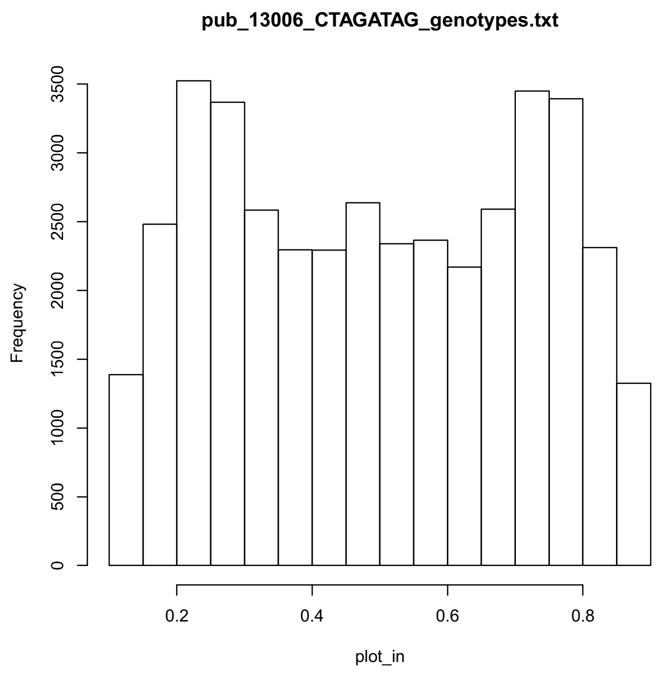
pub_13006_CTAGATAG_genotypes.txt
Frequency
0
500
1000
1500
2000
2500
3000
3500
0.2
0.4
0.6
0.8
plot_in

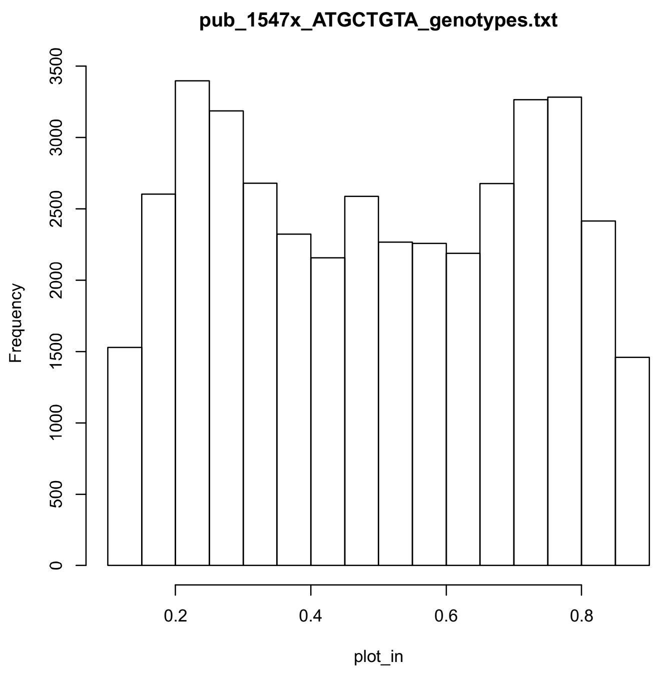
pub_1547x_ATGCTGTA_genotypes.txt
Frequency
0
500
1000
1500
2000
2500
3000
3500
0.2
0.4
0.6
0.8
plot_in

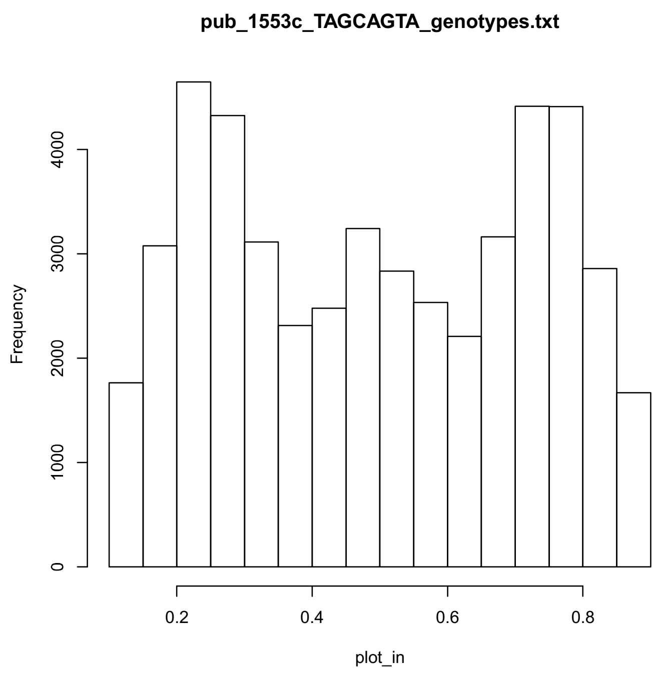
pub_1553c_TAGCAGTA_genotypes.txt
Frequency
0
1000
2000
3000
4000
0.2
0.4
0.6
0.8
plot_in

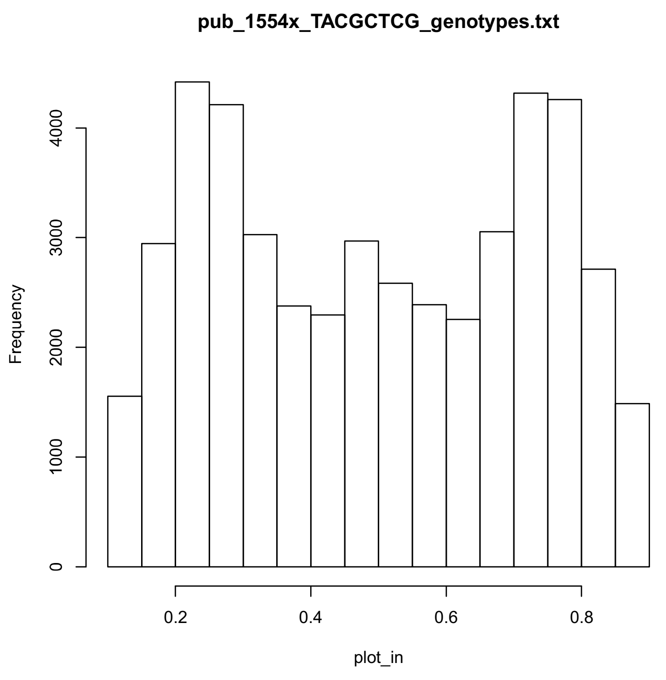
pub_1554x_TACGCTCG_genotypes.txt
Frequency
0
1000
2000
3000
4000
0.2
0.4
0.6
0.8
plot_in

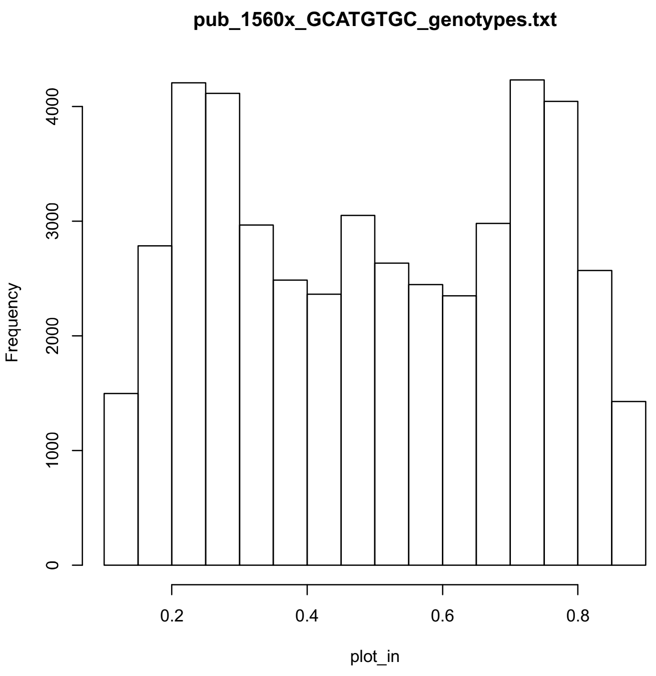
pub_1560x_GCATGTGC_genotypes.txt
Frequency
0
1000
2000
3000
4000
0.2
0.4
0.6
0.8
plot_in

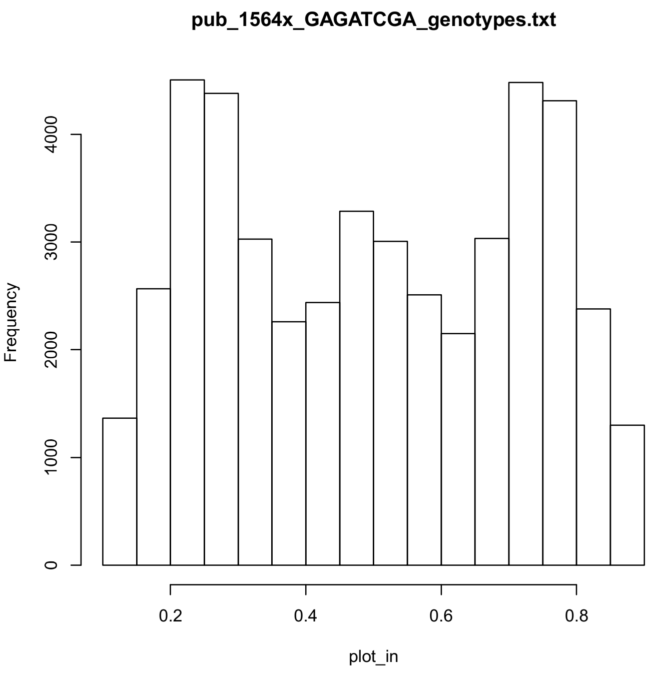
pub_1564x_GAGATCGA_genotypes.txt
Frequency
0
1000
2000
3000
4000
0.2
0.4
0.6
0.8
plot_in

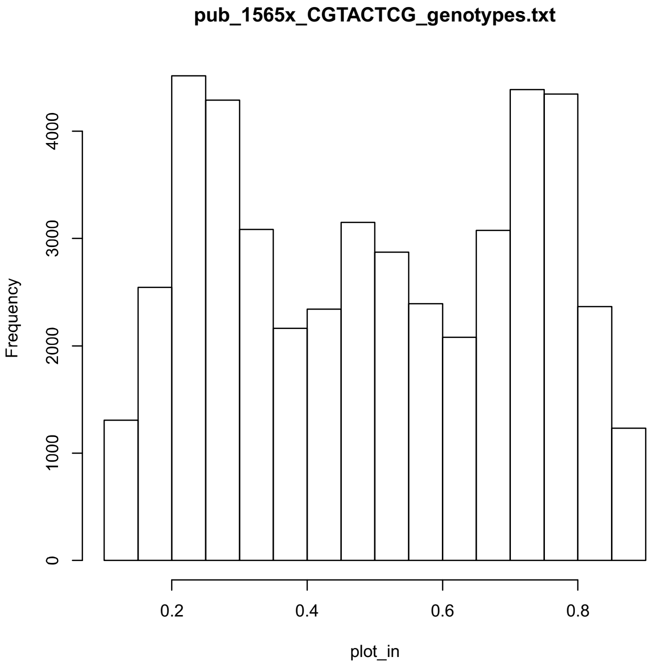
pub_1565x_CGTACTCG_genotypes.txt
Frequency
0
1000
2000
3000
4000
0.2
0.4
0.6
0.8
plot_in

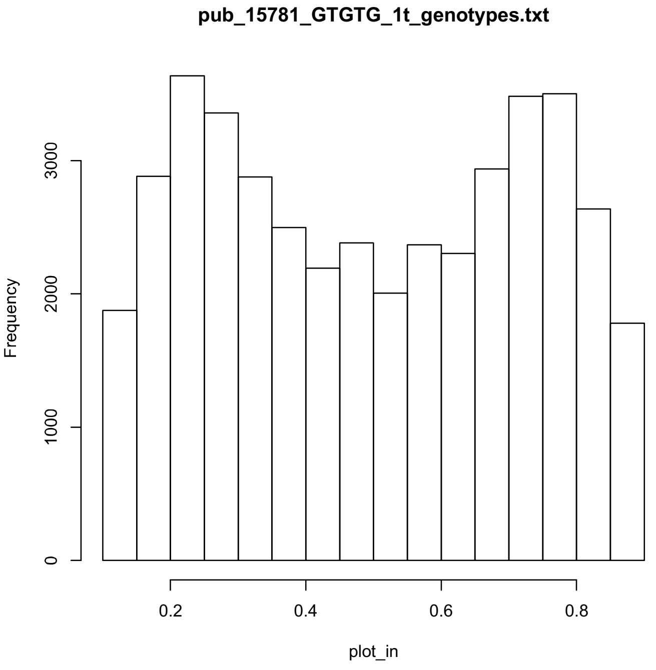
pub_15781_GTGTG_1t_genotypes.txt
Frequency
0
1000
2000
3000
0.2
0.4
0.6
0.8
plot_in

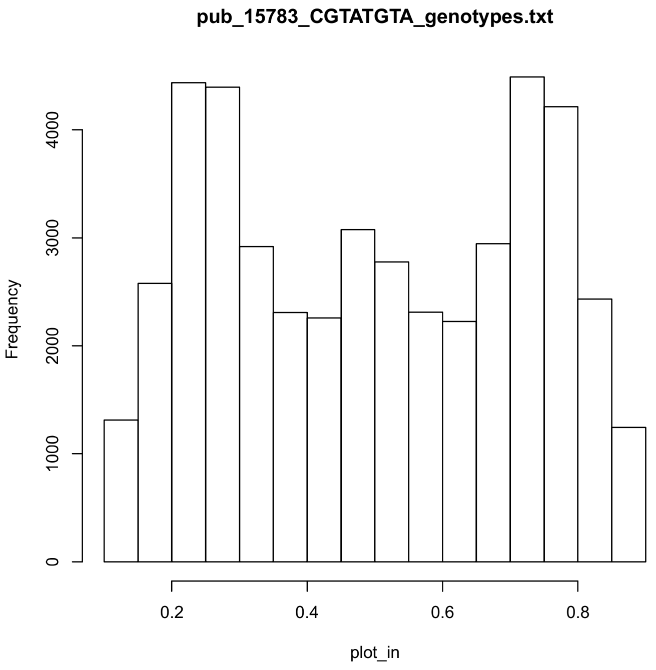
pub_15783_CGTATGTA_genotypes.txt
Frequency
0
1000
2000
3000
4000
0.2
0.4
0.6
0.8
plot_in

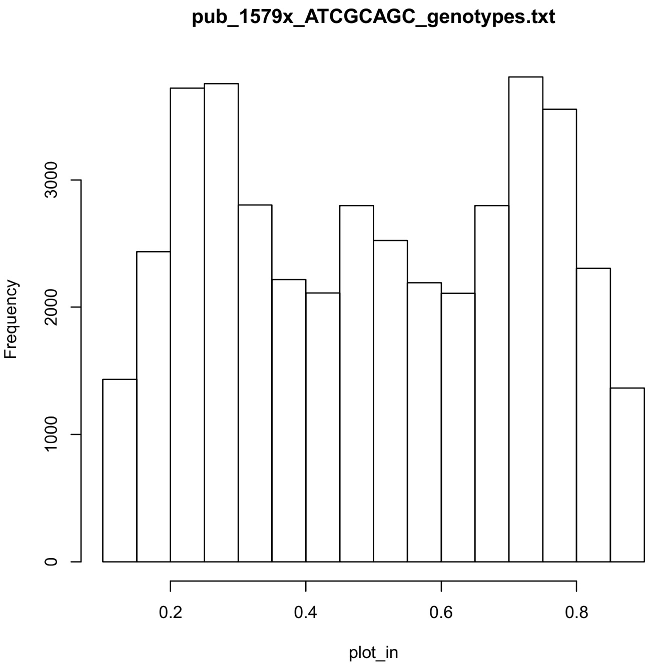
pub_1579x_ATCGCAGC_genotypes.txt
Frequency
0
1000
2000
3000
0.2
0.4
0.6
0.8
plot_in

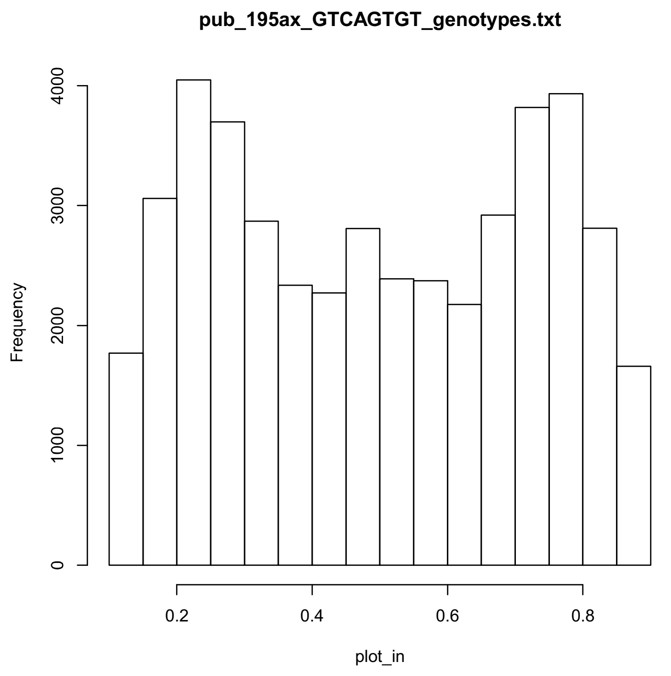
pub_195ax_GTCAGTGT_genotypes.txt
Frequency
0
1000
2000
3000
4000
0.2
0.4
0.6
0.8
plot_in
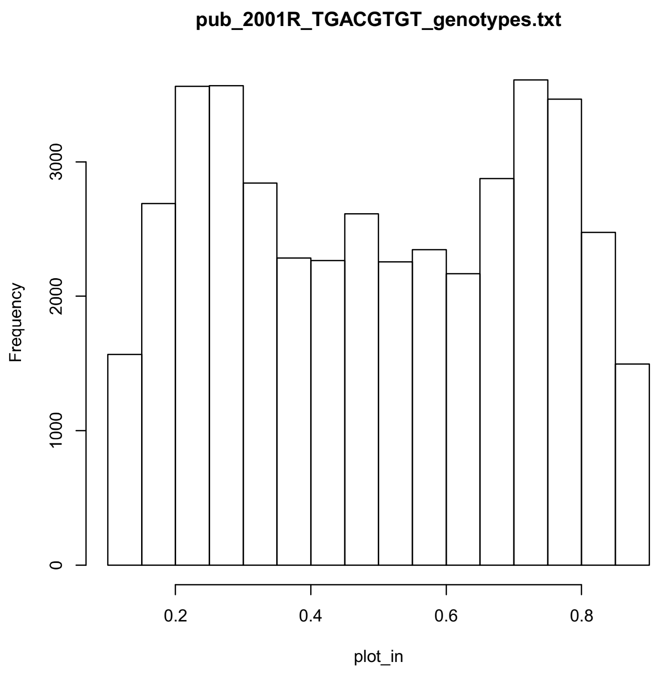
pub_2001R_TGACGTGT_genotypes.txt
Frequency
0
1000
2000
3000
0.2
0.4
0.6
0.8
plot_in
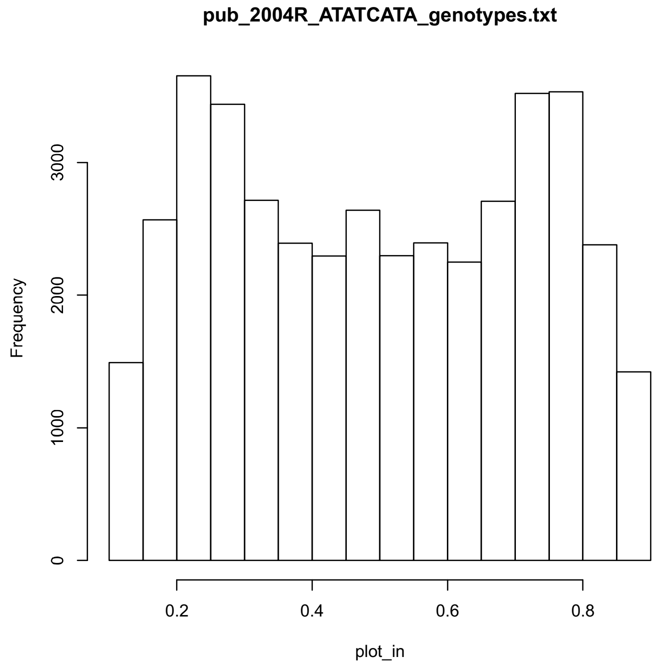
pub_2004R_ATATCATA_genotypes.txt
Frequency
0
1000
2000
3000
0.2
0.4
0.6
0.8
plot_in
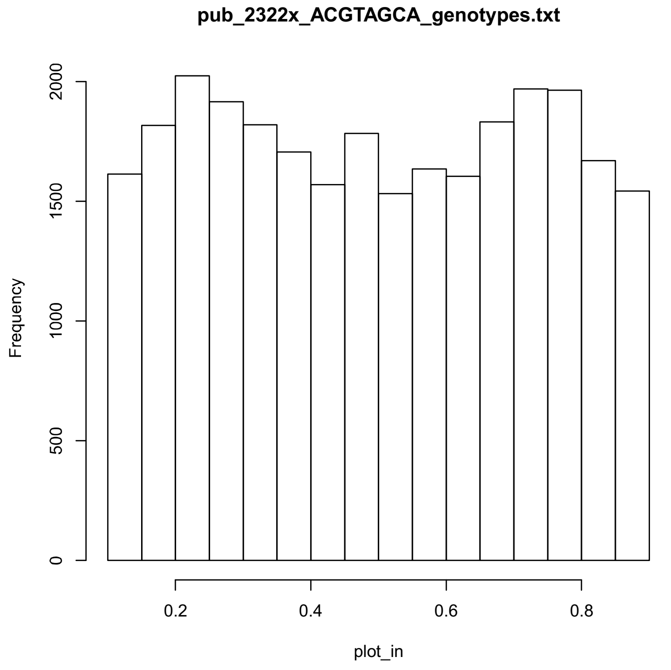
pub_2322x_ACGTAGCA_genotypes.txt
Frequency
0
500
1000
1500
2000
0.2
0.4
0.6
0.8
plot_in
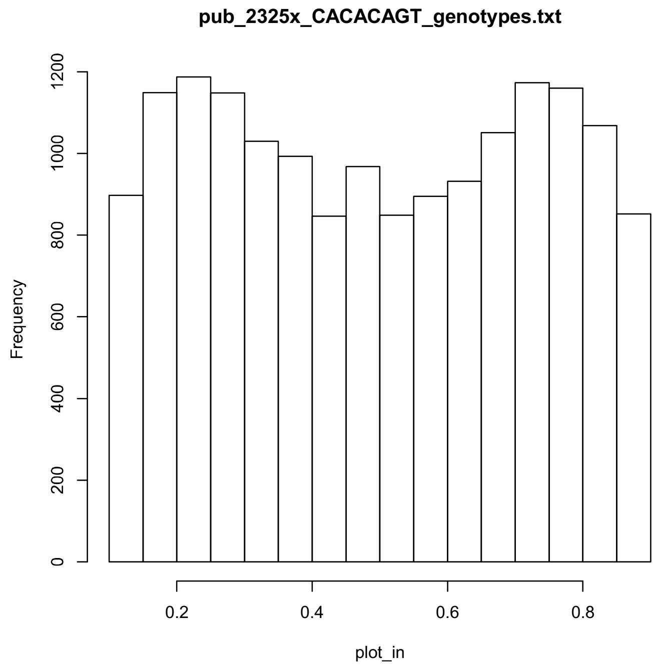
pub_2325x_CACACAGT_genotypes.txt
Frequency
0
200
400
600
800
1000
1200
0.2
0.4
0.6
0.8
plot_in
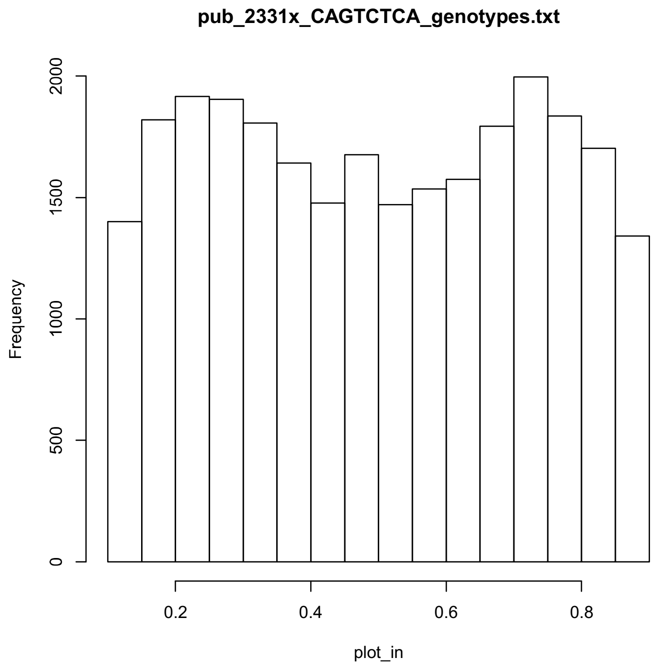
pub_2331x_CAGTCTCA_genotypes.txt
Frequency
0
500
1000
1500
2000
0.2
0.4
0.6
0.8
plot_in

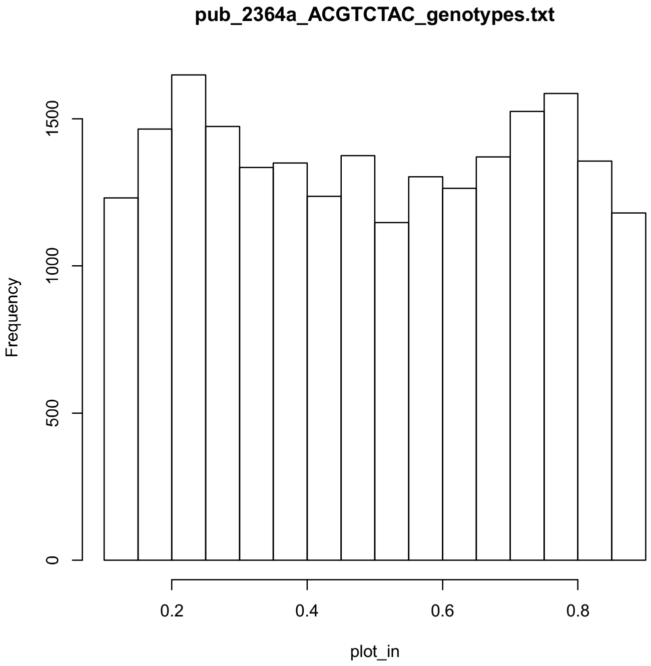
pub_2364a_ACGTCTAC_genotypes.txt
Frequency
0
500
1000
1500
0.2
0.4
0.6
0.8
plot_in

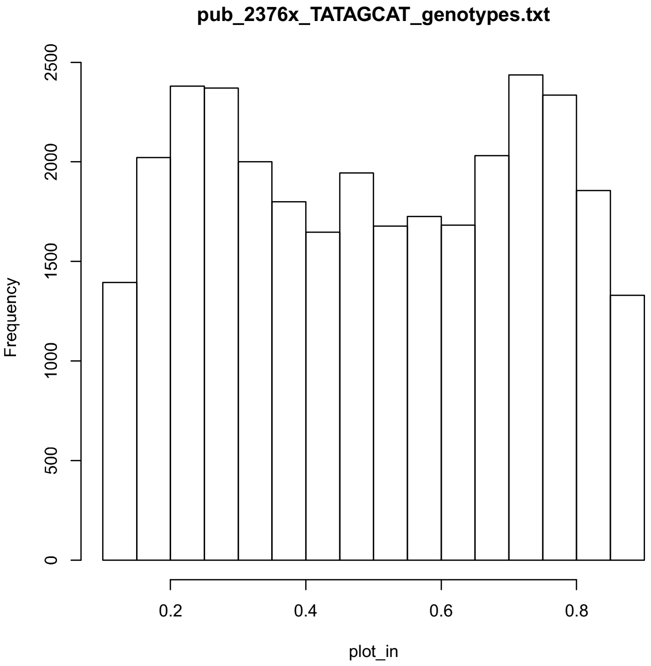
pub_2376x_TATAGCAT_genotypes.txt
Frequency
0
500
1000
1500
2000
2500
0.2
0.4
0.6
0.8
plot_in

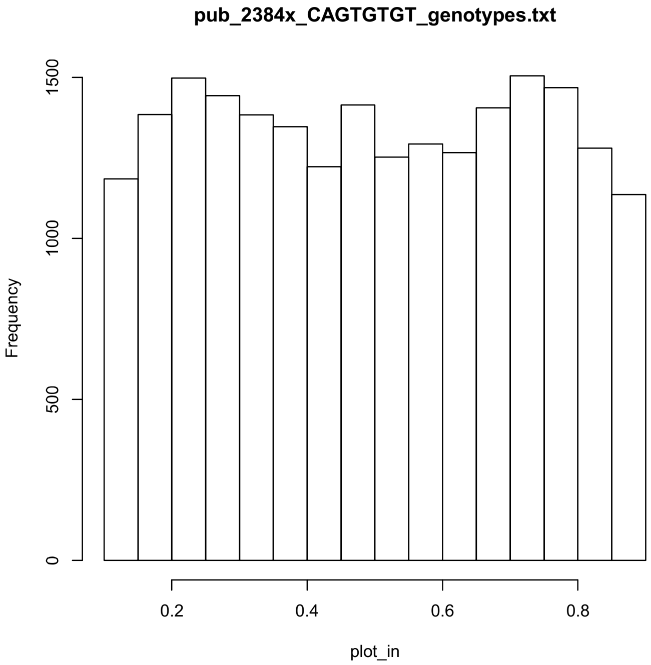
pub_2384x_CAGTGTGT_genotypes.txt
Frequency
0
500
1000
1500
0.2
0.4
0.6
0.8
plot_in

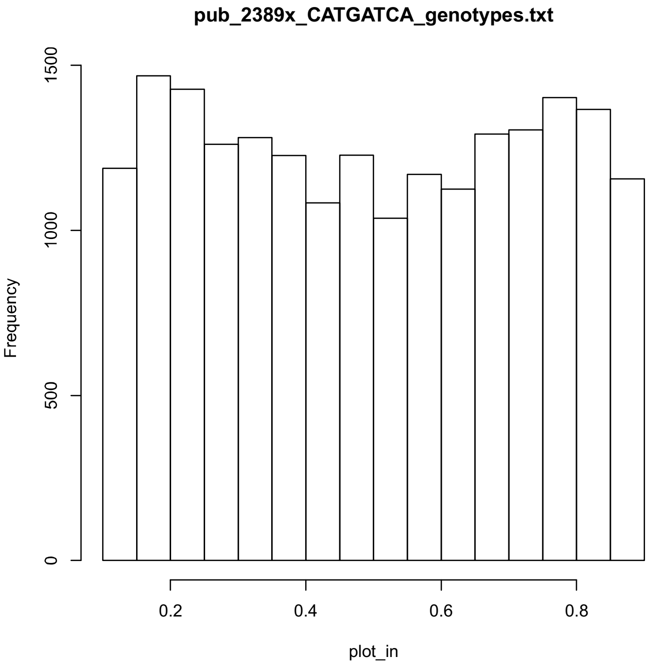
pub_2389x_CATGATCA_genotypes.txt
Frequency
0
500
1000
1500
0.2
0.4
0.6
0.8
plot_in

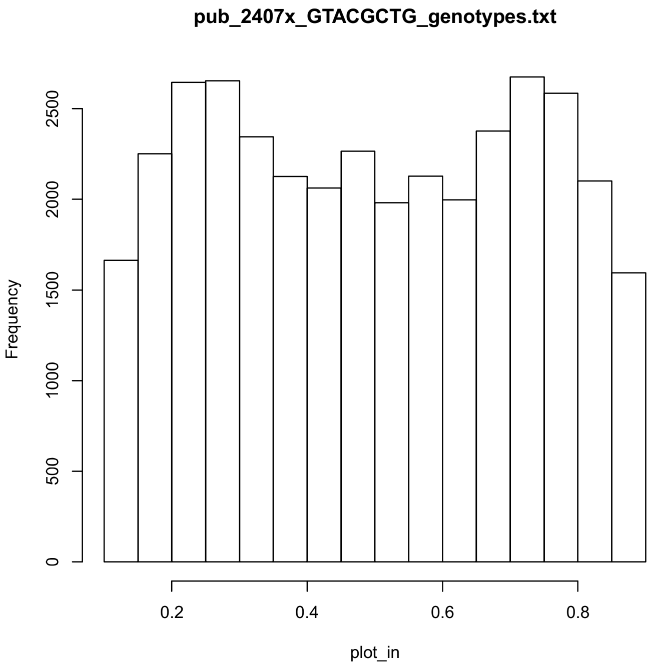
pub_2407x_GTACGCTG_genotypes.txt
Frequency
0
500
1000
1500
2000
2500
0.2
0.4
0.6
0.8
plot_in

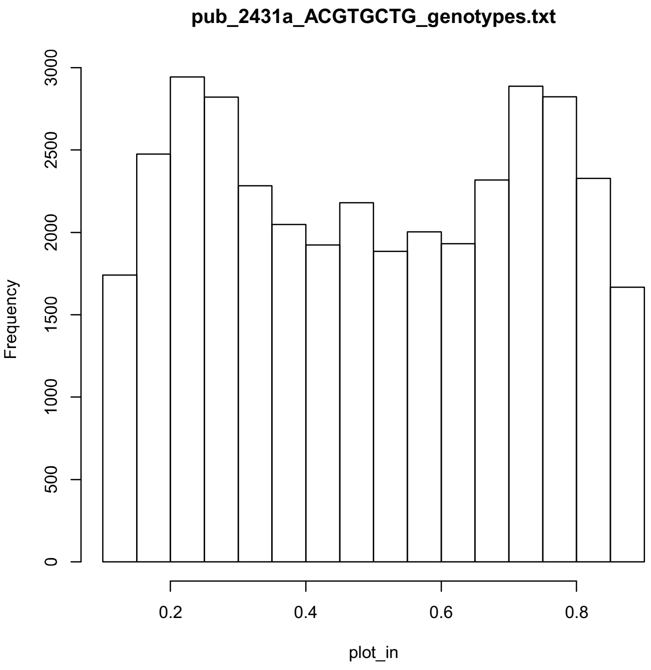
pub_2431a_ACGTGCTG_genotypes.txt
Frequency
0
500
1000
1500
2000
2500
3000
0.2
0.4
0.6
0.8
plot_in

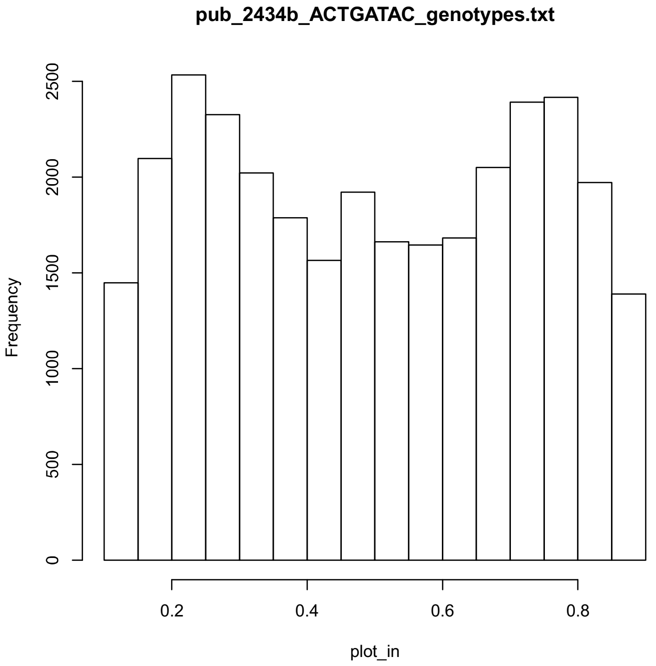
pub_2434b_ACTGATAC_genotypes.txt
Frequency
0
500
1000
1500
2000
2500
0.2
0.4
0.6
0.8
plot_in

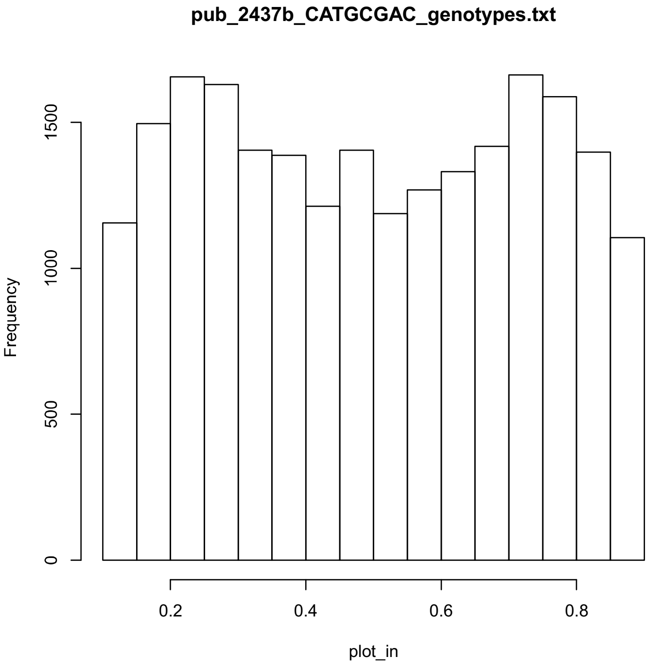
pub_2437b_CATGCGAC_genotypes.txt
Frequency
0
500
1000
1500
0.2
0.4
0.6
0.8
plot_in

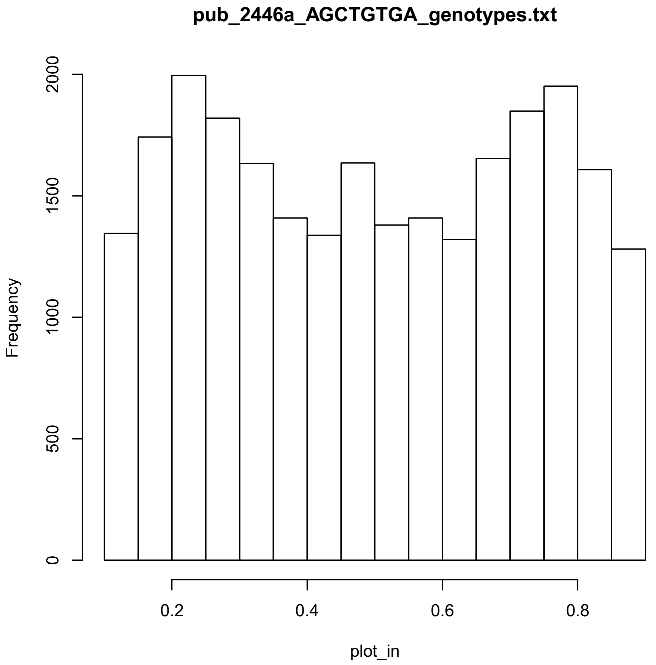
pub_2446a_AGCTGTGA_genotypes.txt
Frequency
0
500
1000
1500
2000
0.2
0.4
0.6
0.8
plot_in

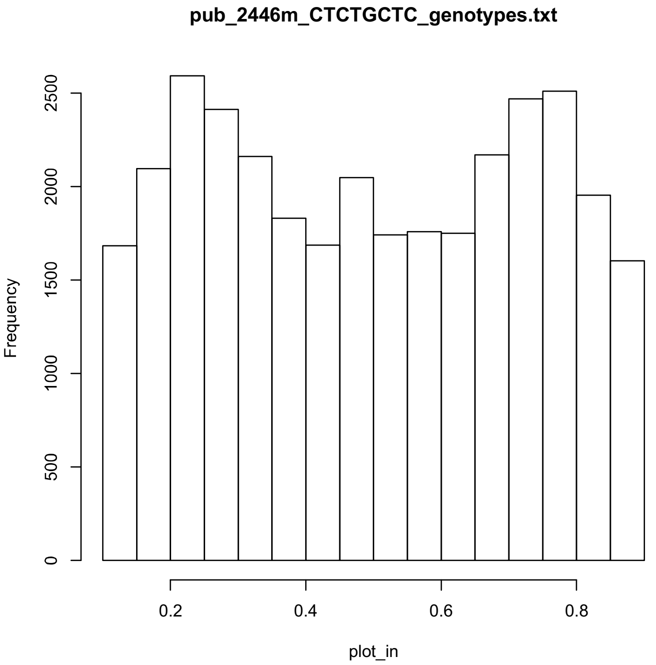
pub_2446m_CTCTGCTC_genotypes.txt
Frequency
0
500
1000
1500
2000
2500
0.2
0.4
0.6
0.8
plot_in

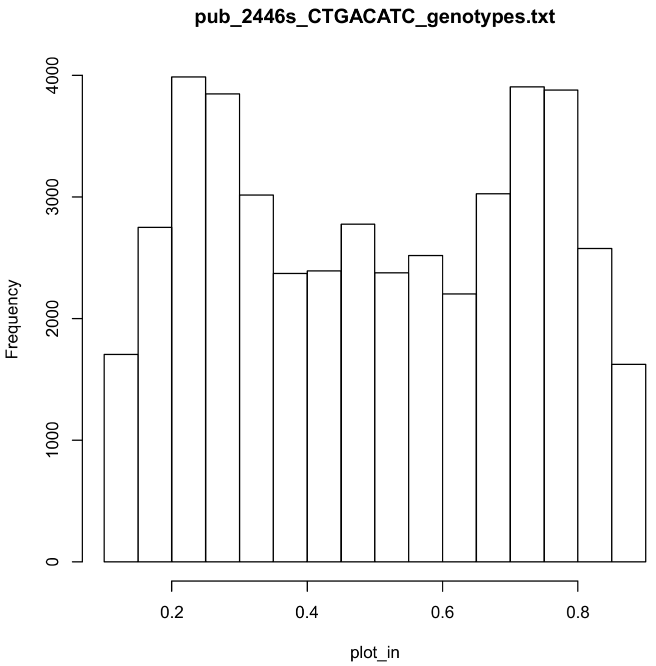
pub_2446s_CTGACATC_genotypes.txt
Frequency
0
1000
2000
3000
4000
0.2
0.4
0.6
0.8
plot_in

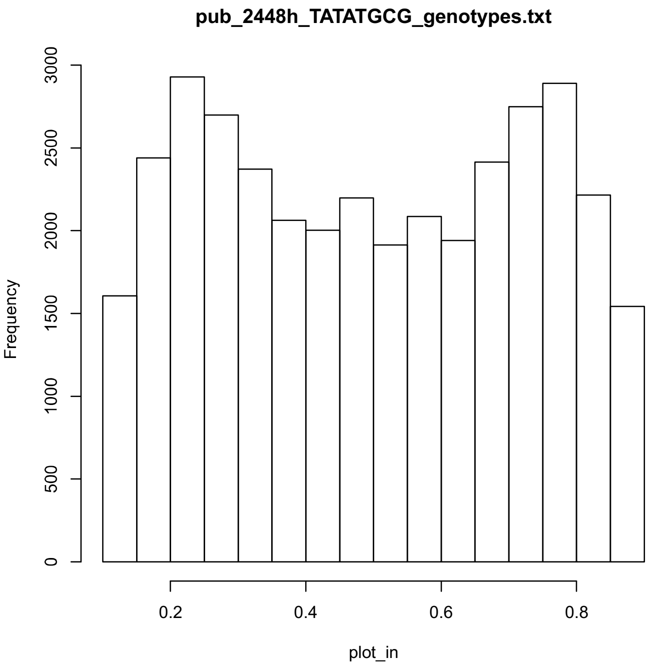
pub_2448h_TATATGCG_genotypes.txt
Frequency
0
500
1000
1500
2000
2500
3000
0.2
0.4
0.6
0.8
plot_in

pub_2448i_GTACATCA_genotypes.txt

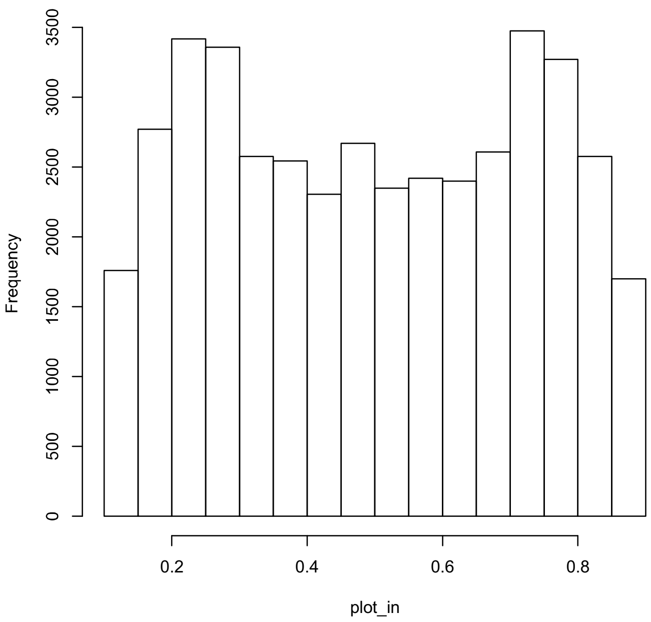

pub_2448j_GTGTACTG_genotypes.txt

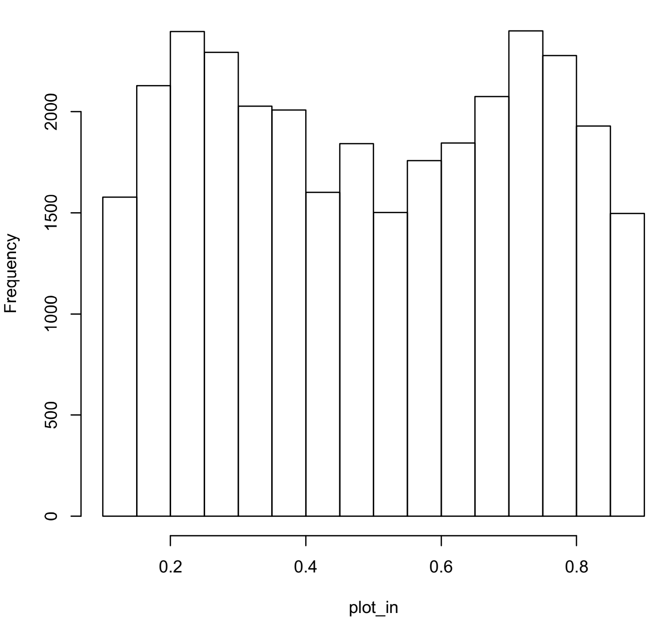

pub_2449b_ATCGCAGC_genotypes.txt

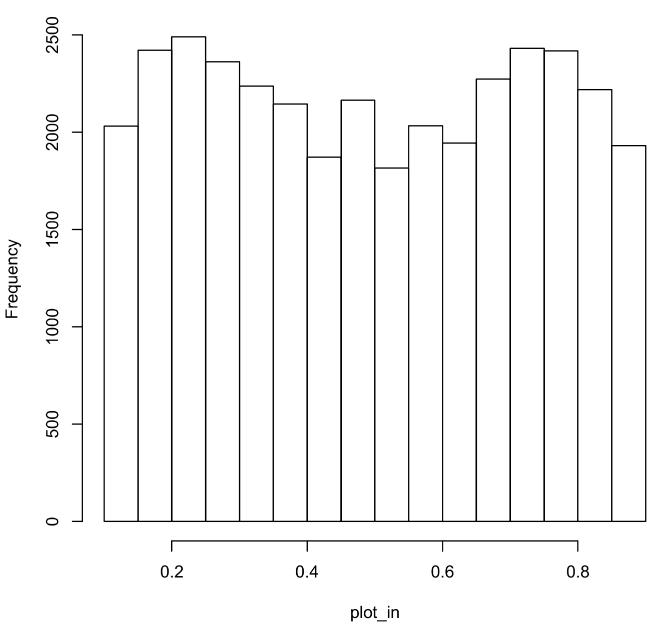

pub_2449c_CGTATGTA_genotypes.txt

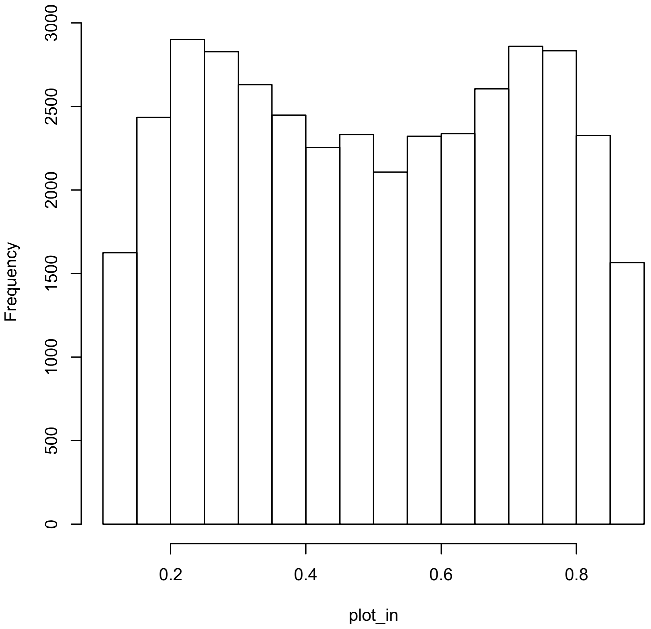

pub_2449d_CGTACTCG_genotypes.txt

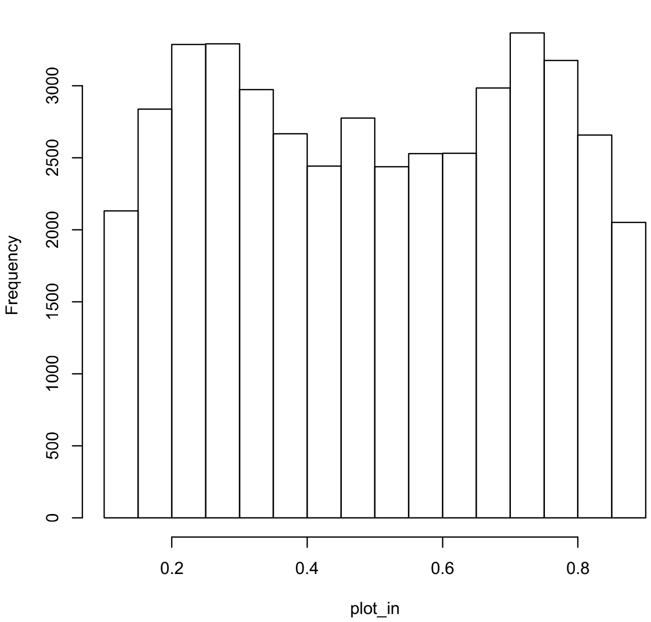

pub_2450a_TGCACTAC_genotypes.txt

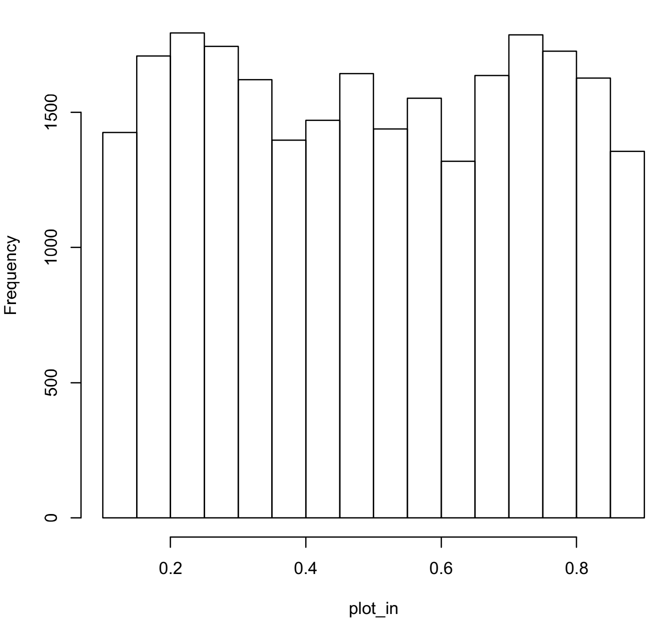

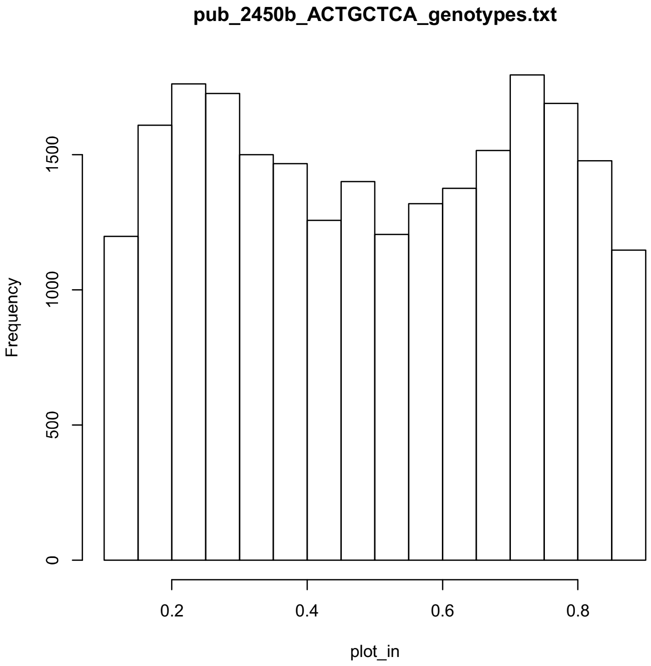
pub_2450b_ACTGCTCA_genotypes.txt
Frequency
0
500
1000
1500
0.2
0.4
0.6
0.8
plot_in

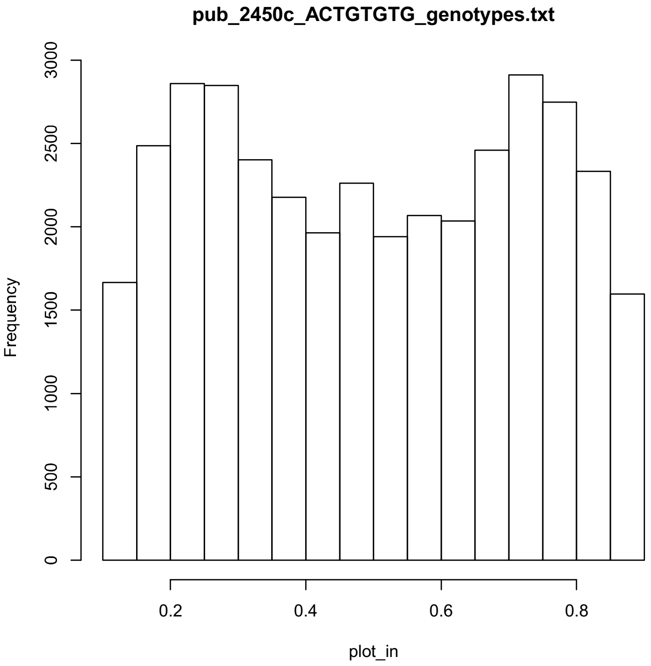
pub_2450c_ACTGTGTG_genotypes.txt
Frequency
0
500
1000
1500
2000
2500
3000
0.2
0.4
0.6
0.8
plot_in

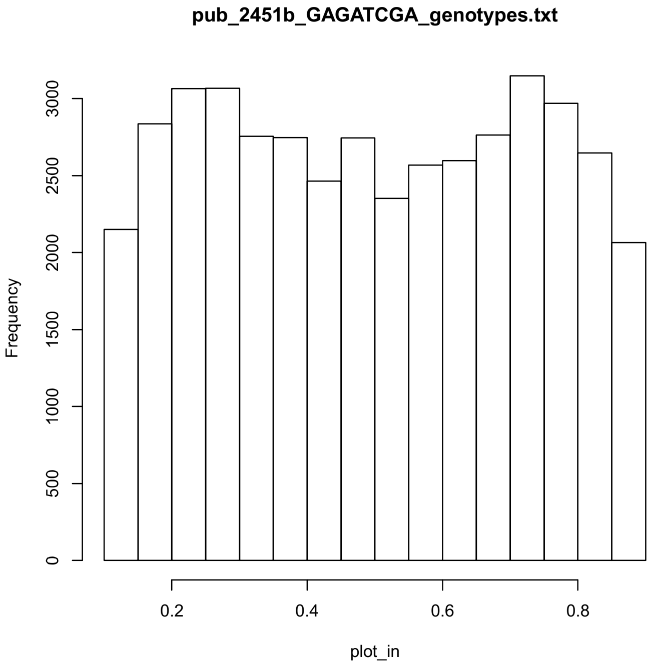
pub_2451b_GAGATCGA_genotypes.txt
Frequency
0
500
1000
1500
2000
2500
3000
0.2
0.4
0.6
0.8
plot_in

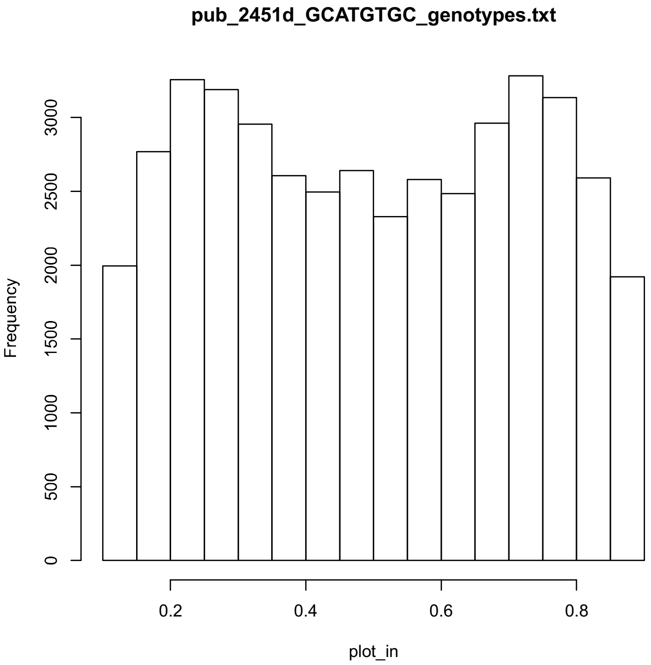
pub_2451d_GCATGTGC_genotypes.txt
Frequency
0
500
1000
1500
2000
2500
3000
0.2
0.4
0.6
0.8
plot_in

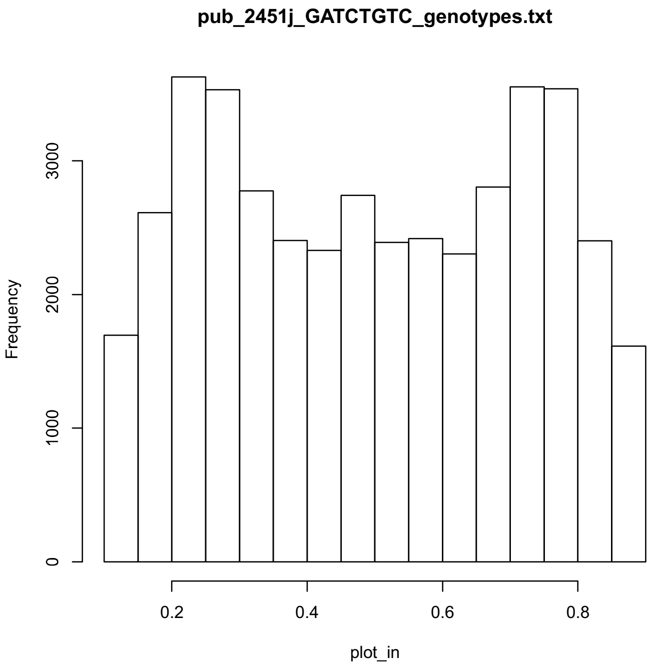
pub_2451j_GATCTGTC_genotypes.txt
Frequency
0
1000
2000
3000
0.2
0.4
0.6
0.8
plot_in

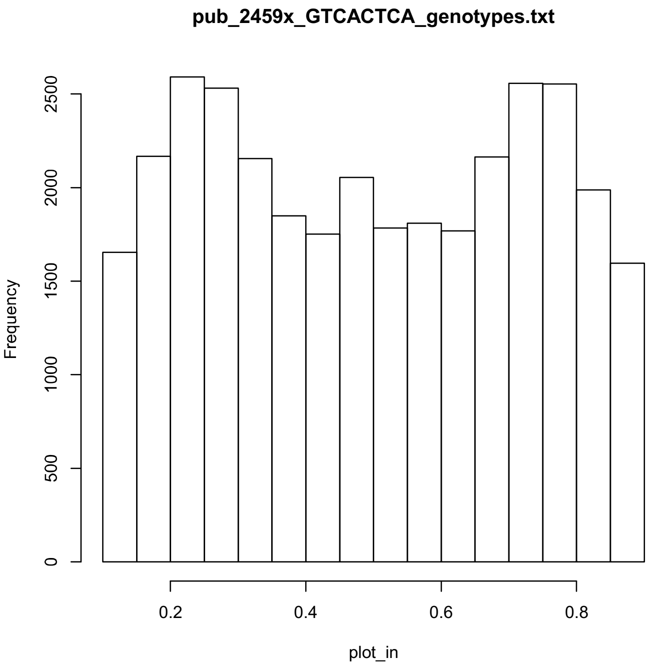
pub_2459x_GTCACTCA_genotypes.txt
Frequency
0
500
1000
1500
2000
2500
0.2
0.4
0.6
0.8
plot_in

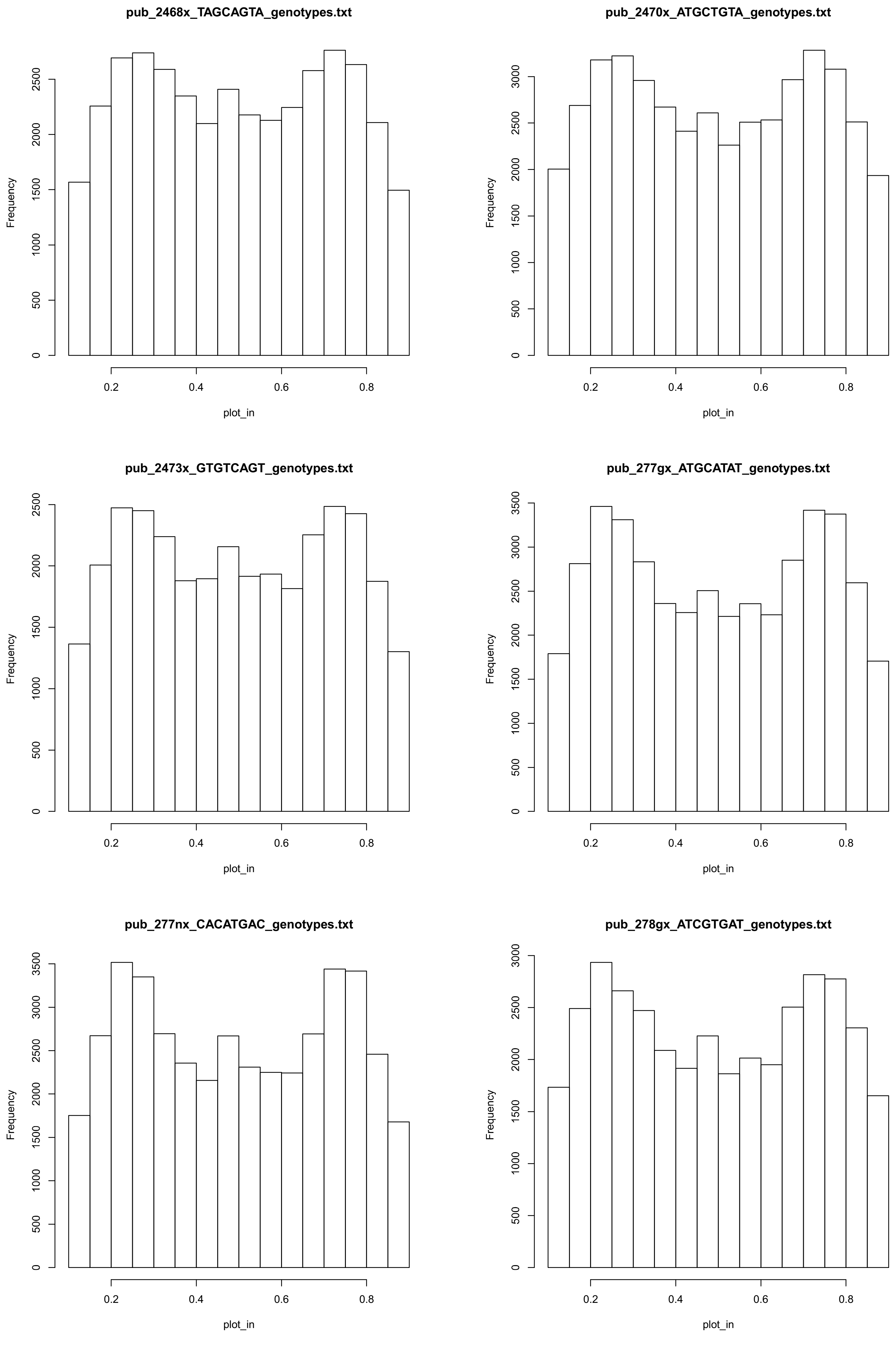
pub_2468x_TAGCAGTA_genotypes.txt
pub_2470x_ATGCTGTA_genotypes.txt
pub_2473x_GTGTCAGT_genotypes.txt
pub_277gx_ATGCATAT_genotypes.txt
pub_277nx_CACATGAC_genotypes.txt
pub_278gx_ATCGTGAT_genotypes.txt
Frequency
plot_in
0.2
0.4
0.6
0.8

pub_283ax_TCGAGTGA_genotypes.txt

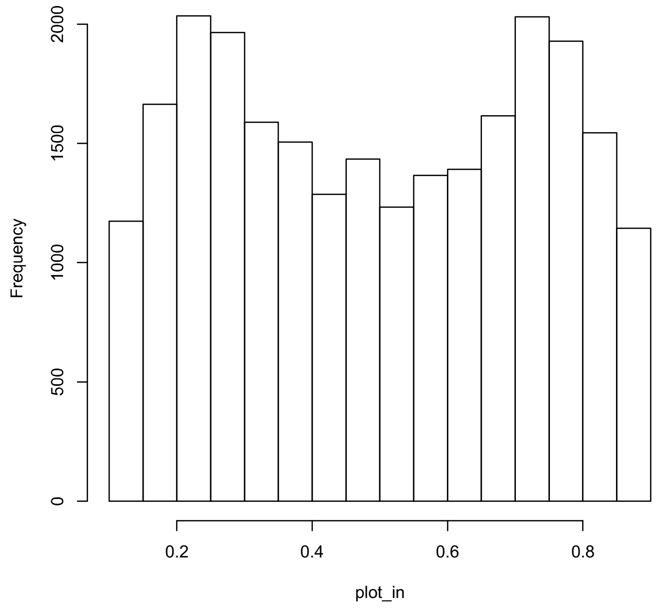

pub_283rx_AGTCACGA_genotypes.txt

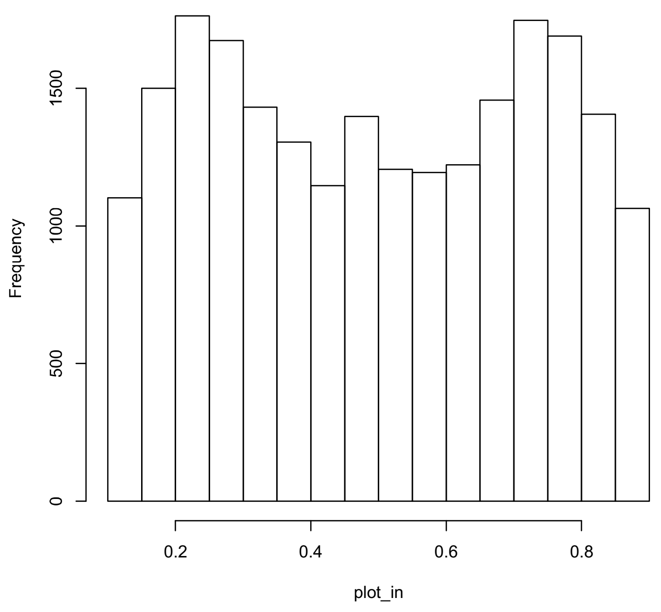

pub_2851x_ATATCATA_genotypes.txt

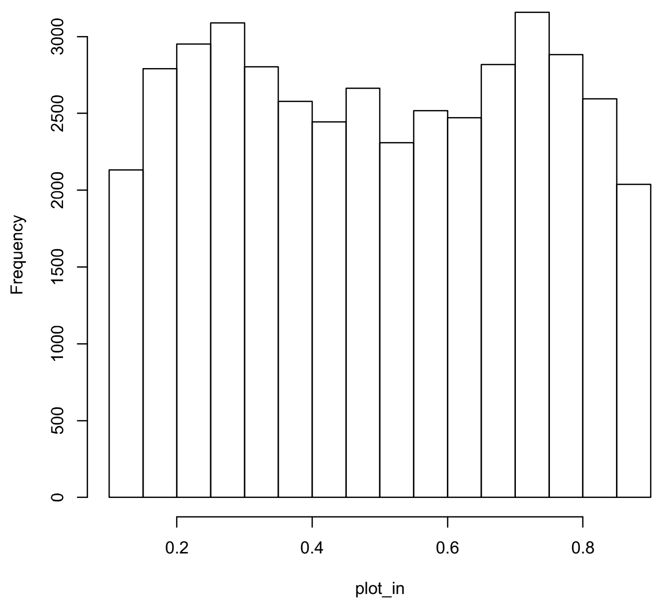

pub_3001R_ATCGAGTA_genotypes.txt

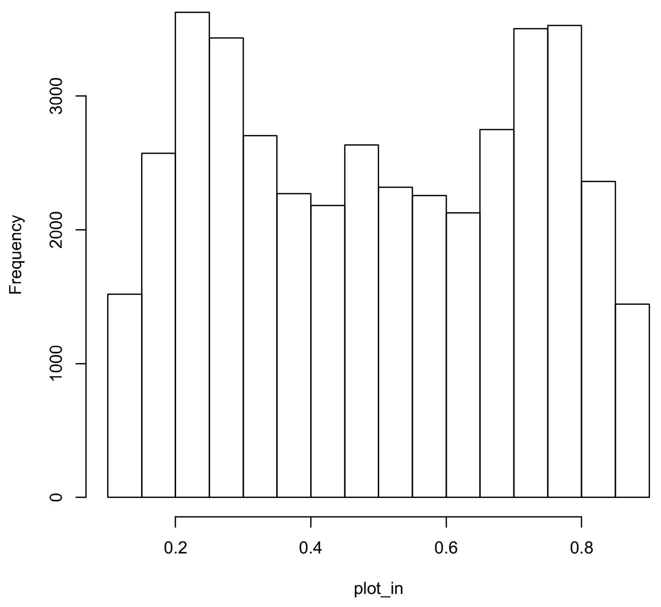

pub_3251x_GAGAT_1t_genotypes.txt

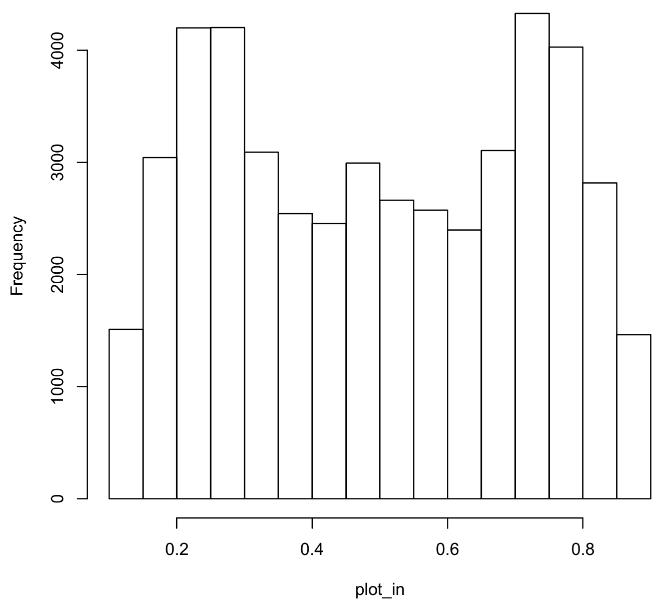

pub_3253x_CTAGATAG_genotypes.txt

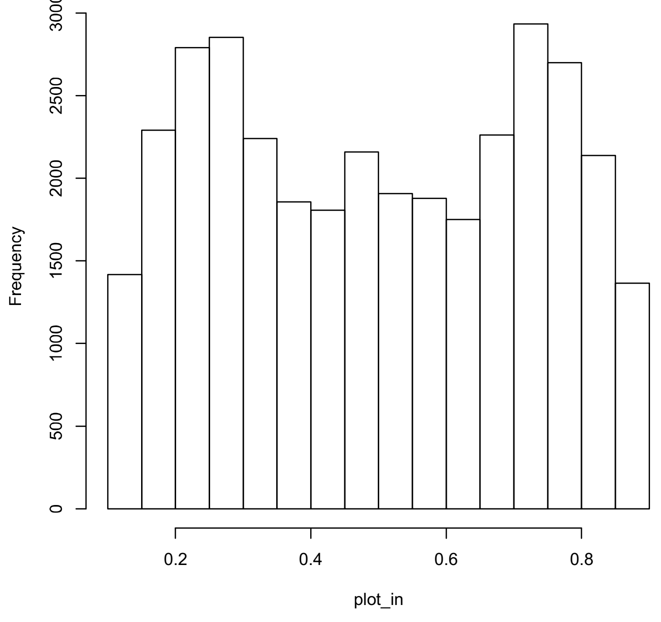

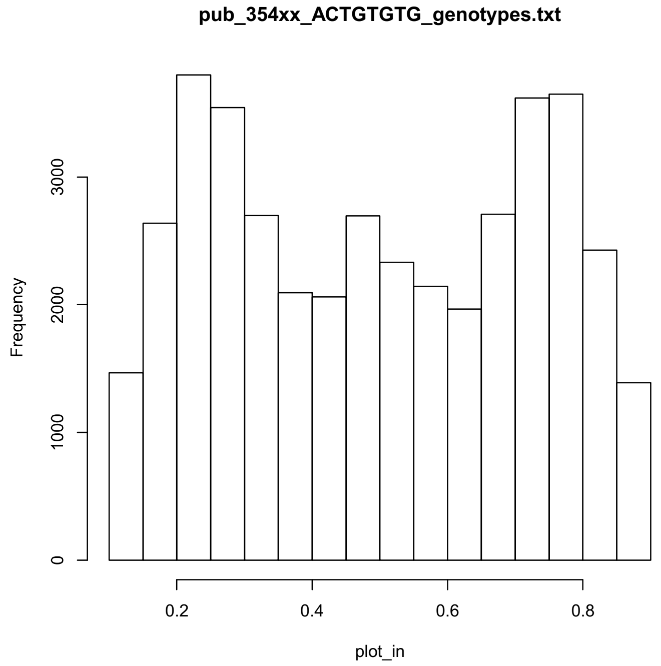
pub_354xx_ACTGTGTG_genotypes.txt
Frequency
0
1000
2000
3000
0.2
0.4
0.6
0.8
plot_in

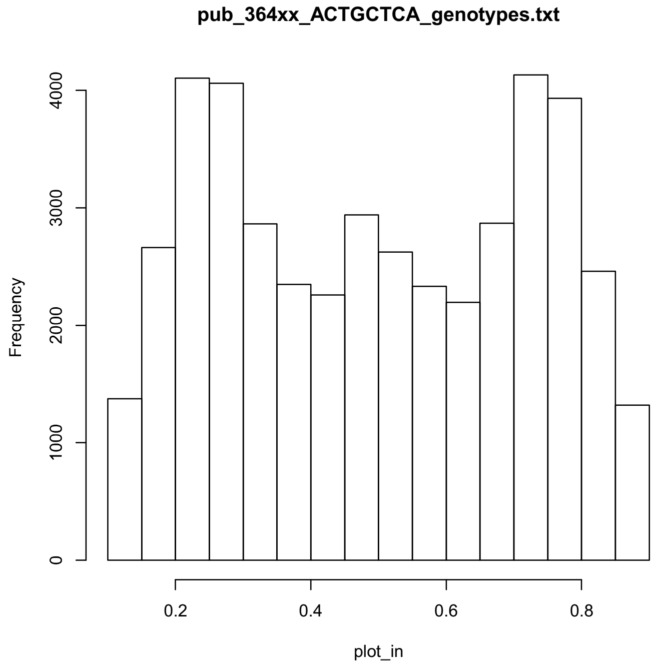
pub_364xx_ACTGCTCA_genotypes.txt
Frequency
0
1000
2000
3000
4000
0.2
0.4
0.6
0.8
plot_in

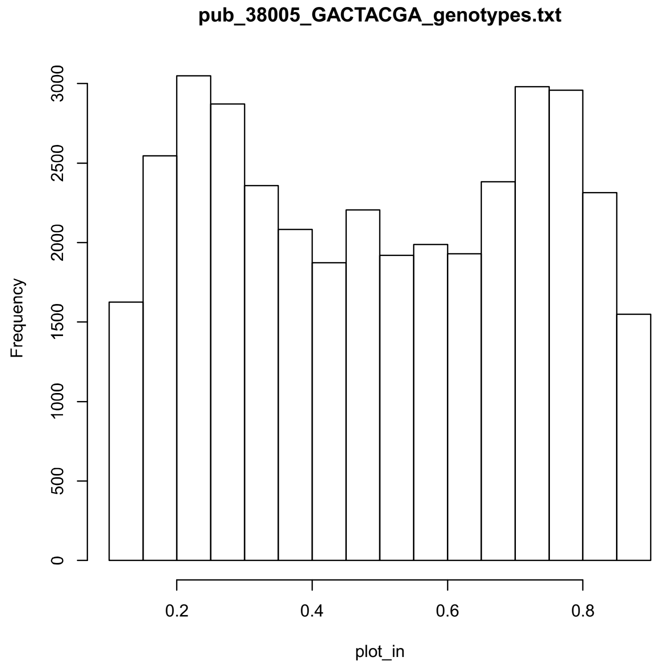
pub_38005_GACTACGA_genotypes.txt
Frequency
0
500
1000
1500
2000
2500
3000
0.2
0.4
0.6
0.8
plot_in

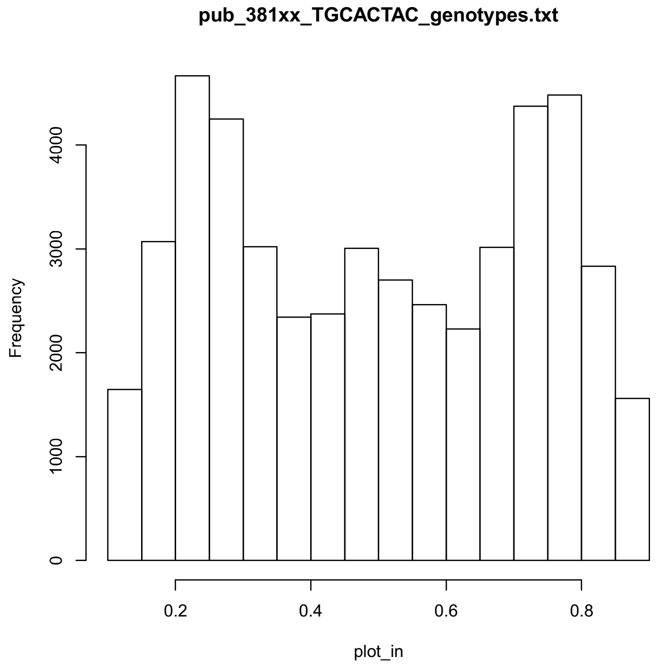
pub_381xx_TGCACTAC_genotypes.txt
Frequency
0
1000
2000
3000
4000
0.2
0.4
0.6
0.8
plot_in

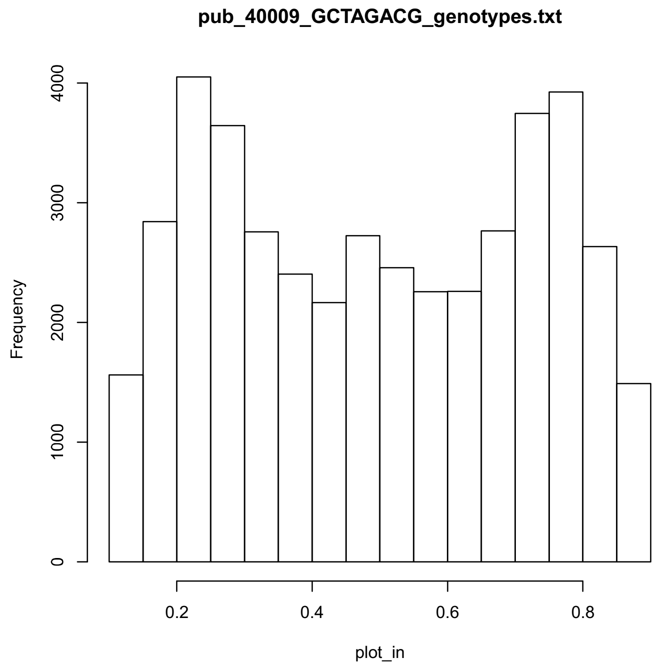
pub_40009_GCTAGACG_genotypes.txt
Frequency
0
1000
2000
3000
4000
0.2
0.4
0.6
0.8
plot_in

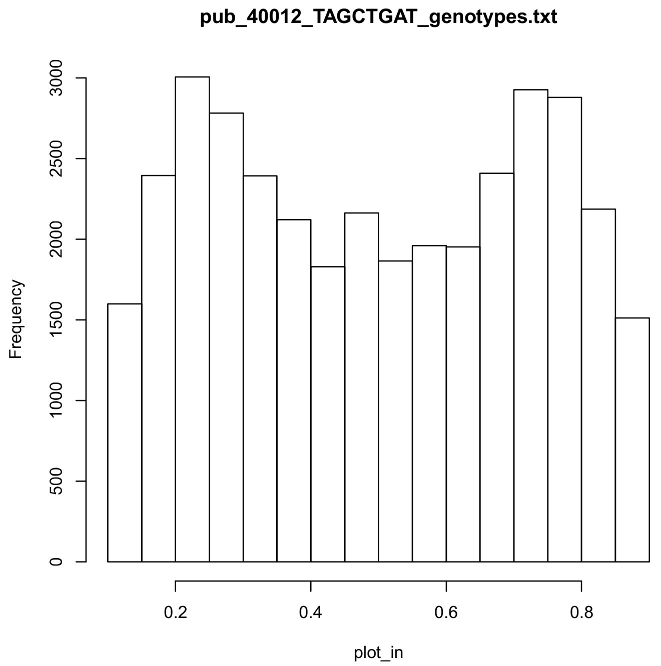
pub_40012_TAGCTGAT_genotypes.txt
Frequency
0
500
1000
1500
2000
2500
3000
0.2
0.4
0.6
0.8
plot_in

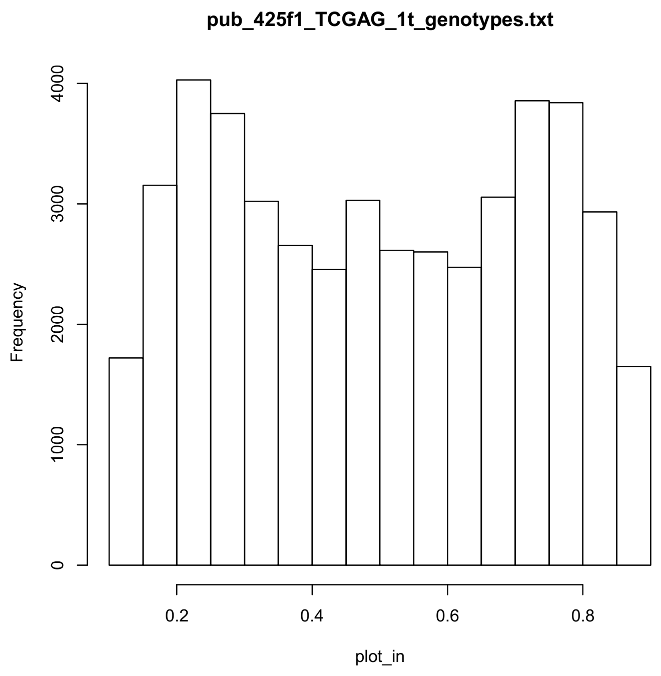
pub_425f1_TCGAG_1t_genotypes.txt
Frequency
0
1000
2000
3000
4000
0.2
0.4
0.6
0.8
plot_in

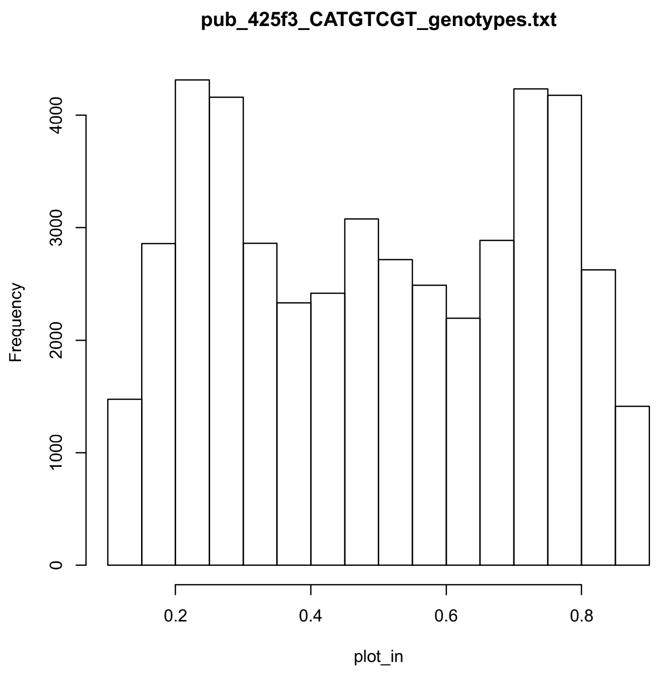
pub_425f3_CATGTCGT_genotypes.txt
Frequency
0
1000
2000
3000
4000
0.2
0.4
0.6
0.8
plot_in

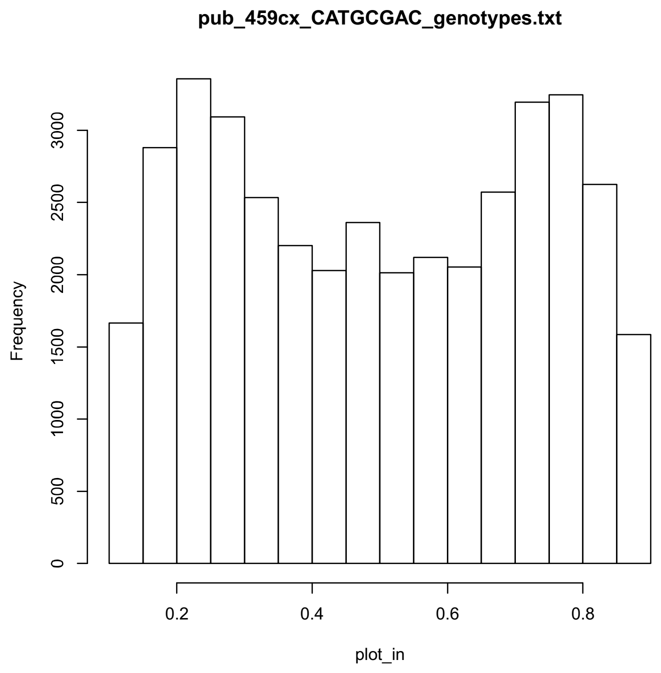
pub_459cx_CATGCGAC_genotypes.txt
Frequency
0
500
1000
1500
2000
2500
3000
0.2
0.4
0.6
0.8
plot_in

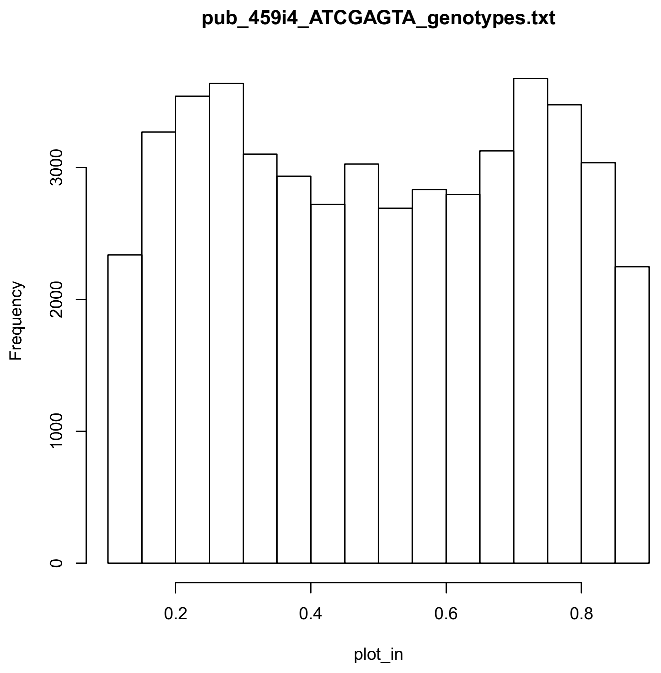
pub_459i4_ATCGAGTA_genotypes.txt
Frequency
0
1000
2000
3000
0.2
0.4
0.6
0.8
plot_in

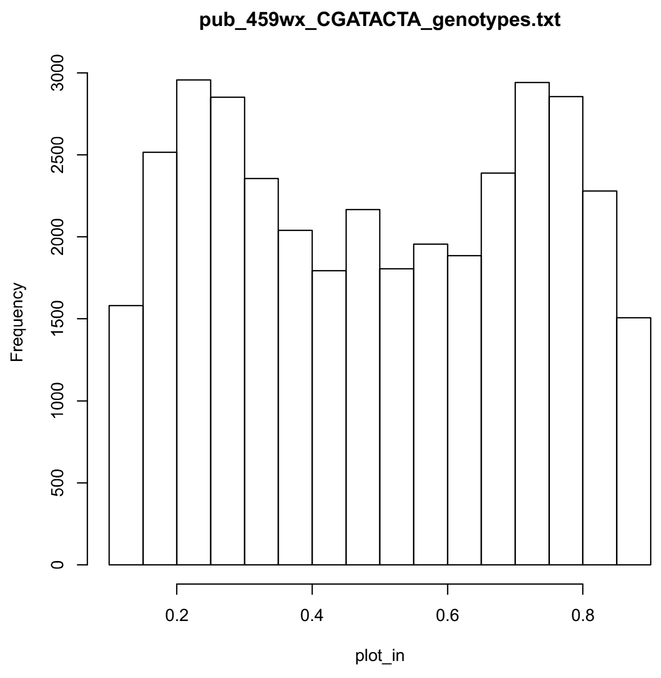
pub_459wx_CGATACTA_genotypes.txt
Frequency
0
500
1000
1500
2000
2500
3000
0.2
0.4
0.6
0.8
plot_in

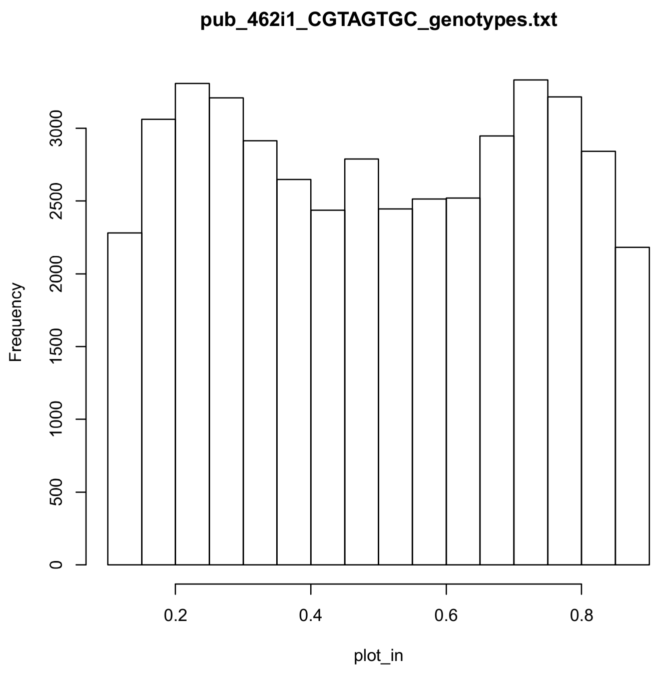
pub_462i1_CGTAGTGC_genotypes.txt
Frequency
0
500
1000
1500
2000
2500
3000
0.2
0.4
0.6
0.8
plot_in

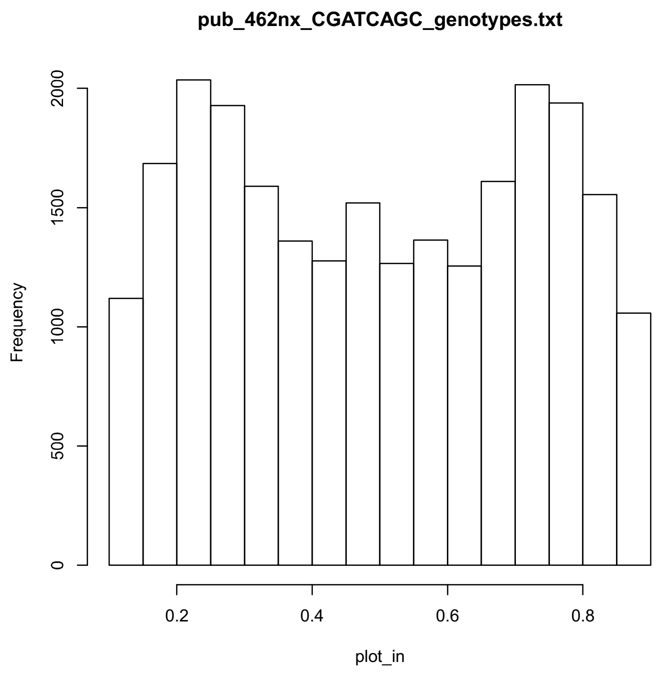
pub_462nx_CGATCAGC_genotypes.txt
Frequency
0
500
1000
1500
2000
0.2
0.4
0.6
0.8
plot_in

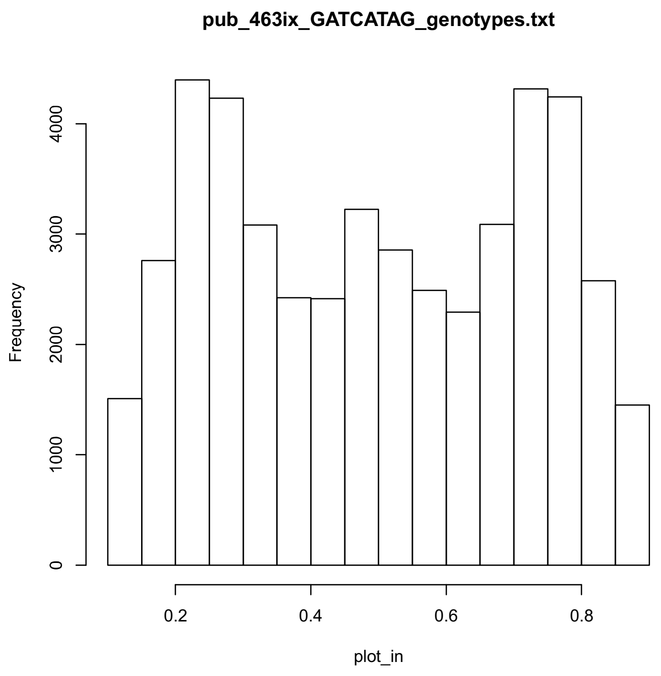
pub_463ix_GATCATAG_genotypes.txt
Frequency
0
1000
2000
3000
4000
0.2
0.4
0.6
0.8
plot_in

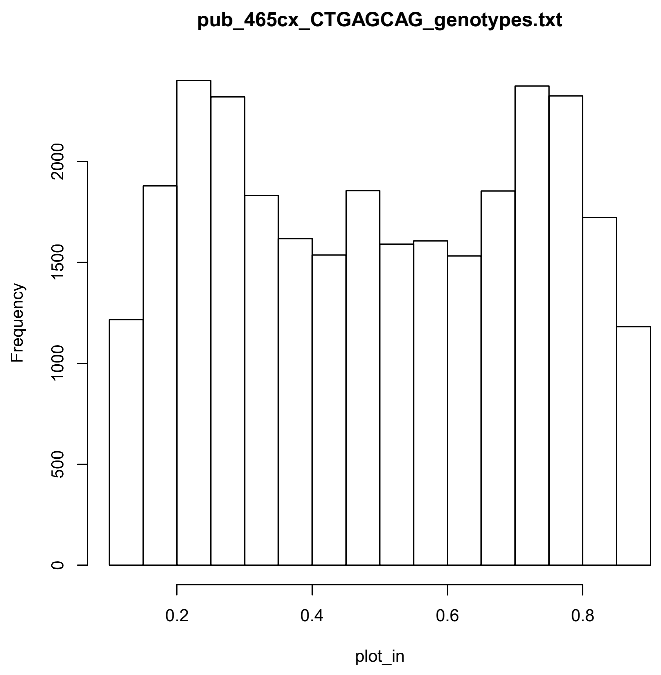
pub_465cx_CTGAGCAG_genotypes.txt
Frequency
0
500
1000
1500
2000
0.2
0.4
0.6
0.8
plot_in

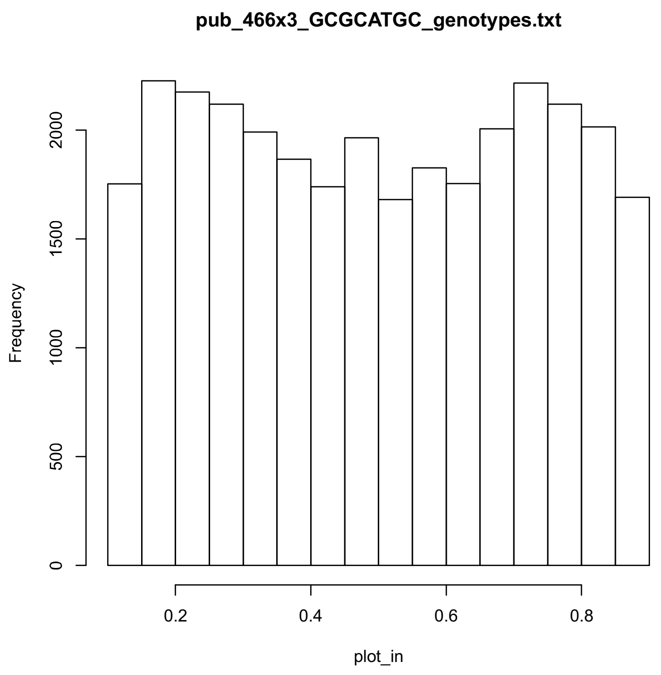
pub_466x3_GCGCATGC_genotypes.txt
Frequency
0
500
1000
1500
2000
0.2
0.4
0.6
0.8
plot_in

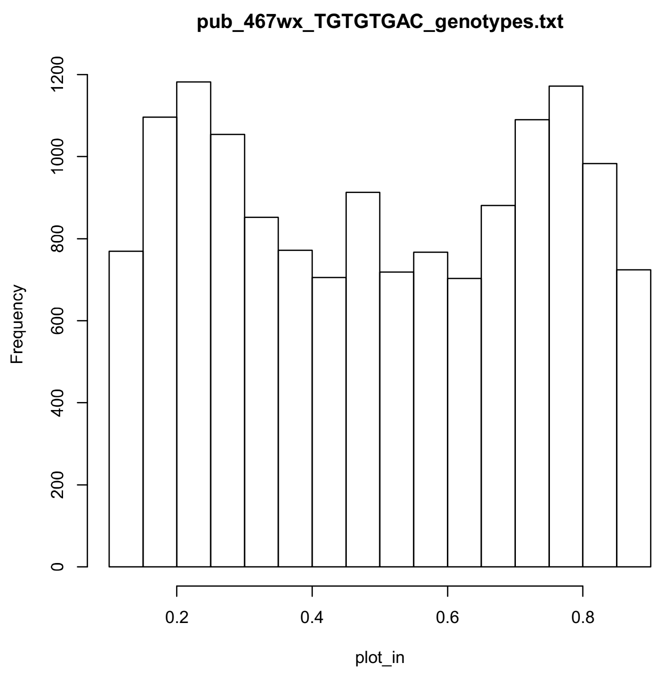
pub_467wx_TGTGTGAC_genotypes.txt
Frequency
0
200
400
600
800
1000
1200
0.2
0.4
0.6
0.8
plot_in

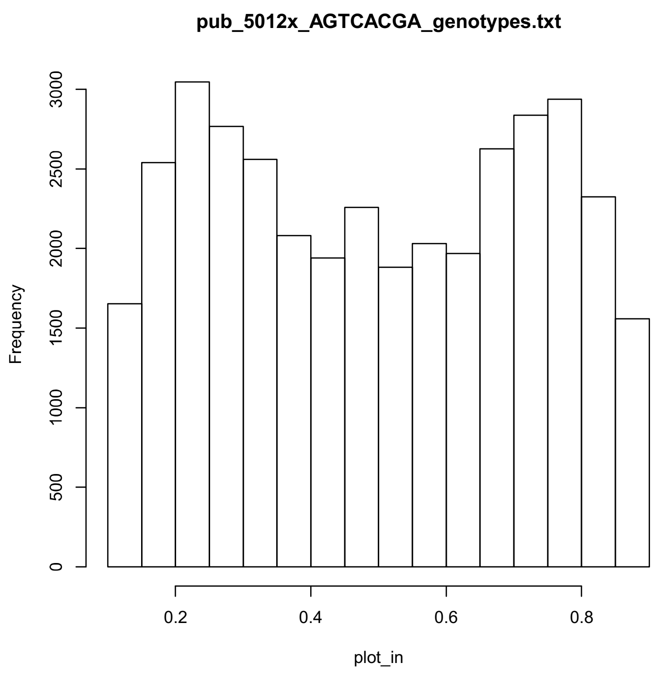
pub_5012x_AGTCACGA_genotypes.txt
Frequency
0
500
1000
1500
2000
2500
3000
0.2
0.4
0.6
0.8
plot_in

pub_5014x_AGTCTGCT_genotypes.txt

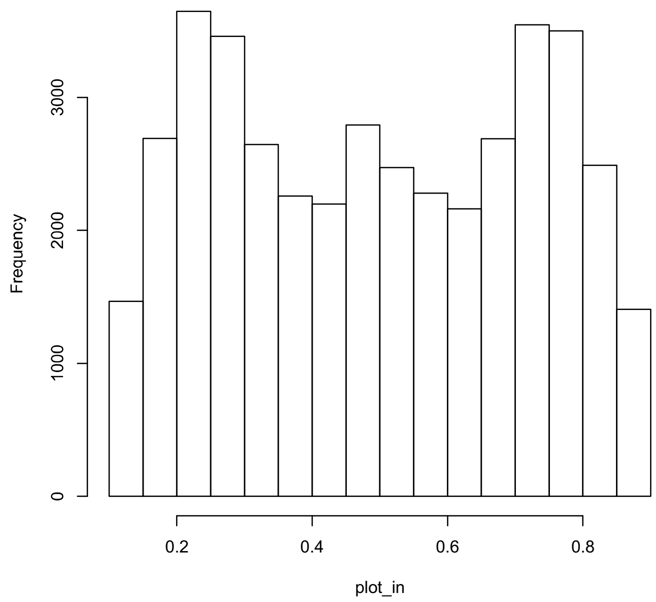

pub_567xx_TGCAGCTG_genotypes.txt

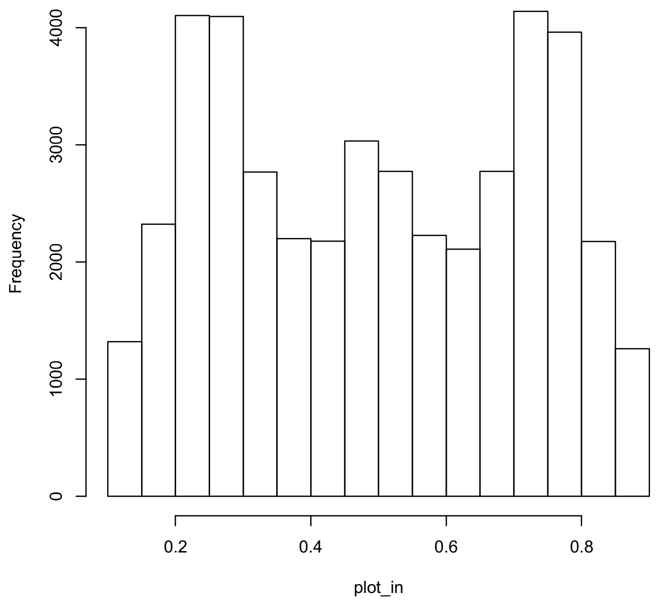

pub_575xx_TATAGCAT_genotypes.txt

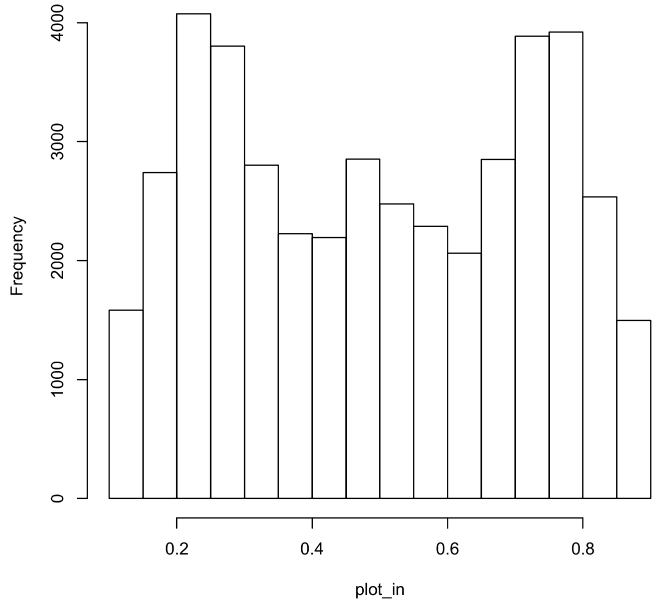

pub_576xx_GTGTCAGT_genotypes.txt

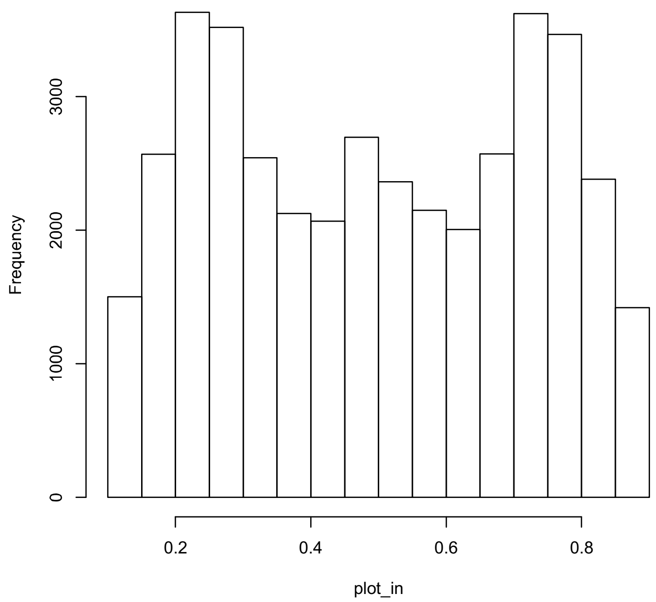

pub_579xx_CTAGCTCT_genotypes.txt

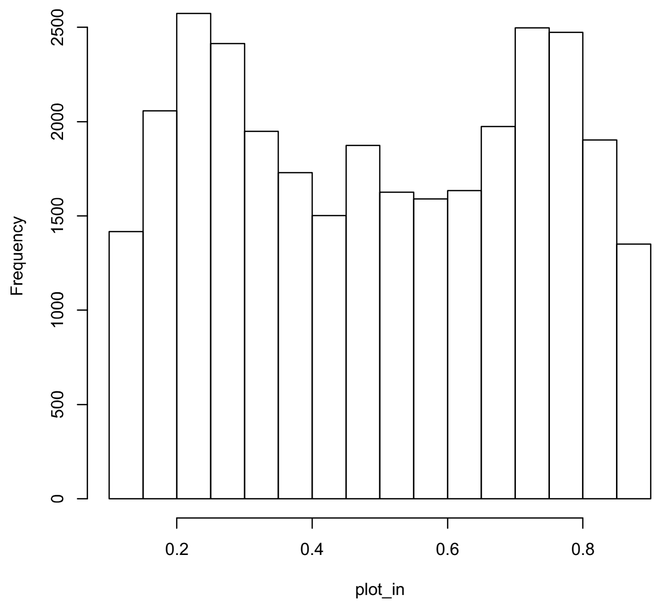

pub_583xx_TGACC_1t_genotypes.txt

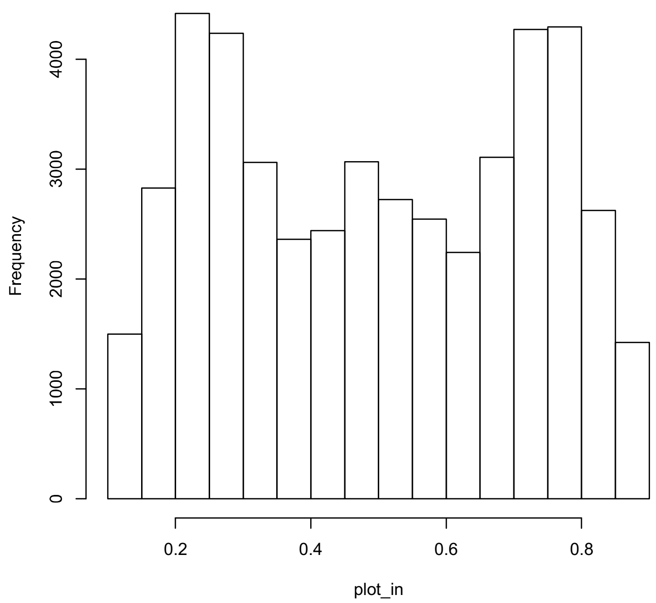

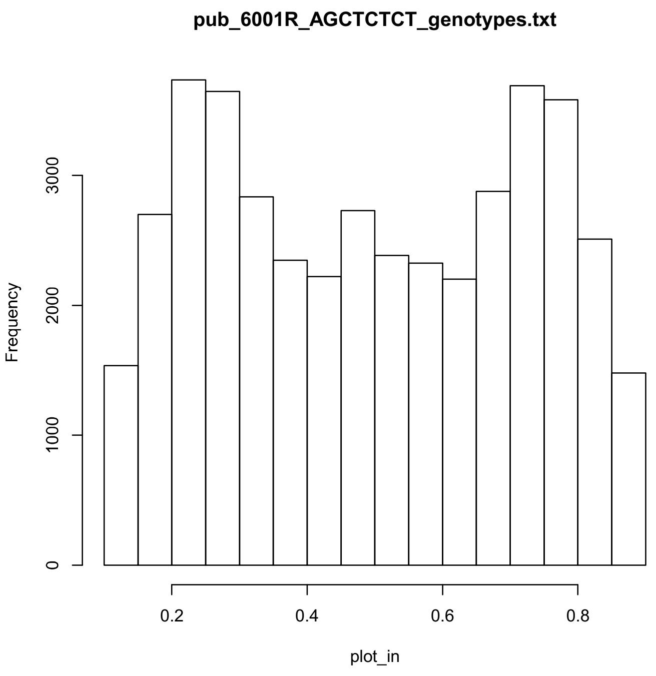
pub_6001R_AGCTCTCT_genotypes.txt
Frequency
0
1000
2000
3000
plot_in
0.2
0.4
0.6
0.8

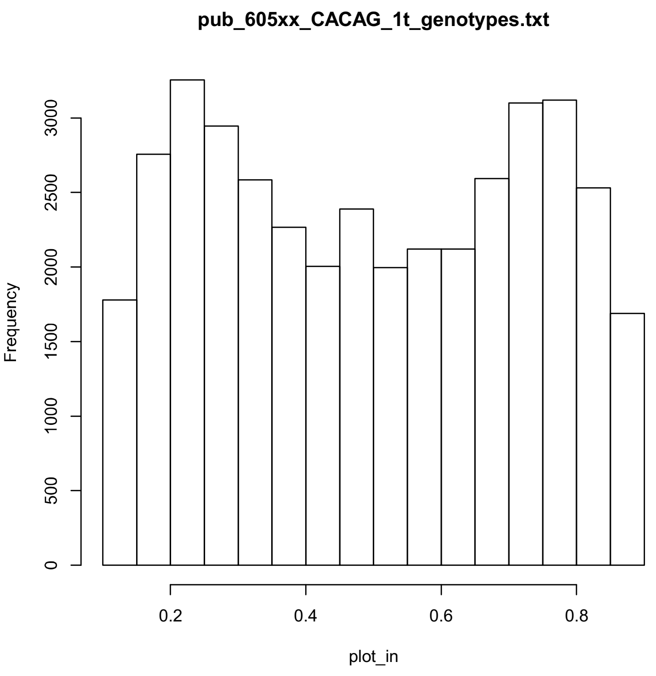
pub_605xx_CACAG_1t_genotypes.txt
Frequency
0
500
1000
1500
2000
2500
3000
plot_in
0.2
0.4
0.6
0.8

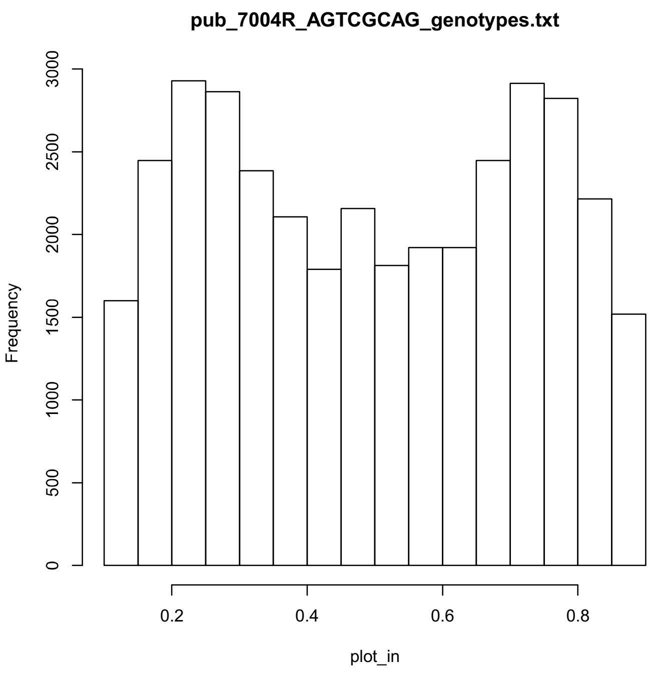
pub_7004R_AGTCGCAG_genotypes.txt
Frequency
0
500
1000
1500
2000
2500
3000
plot_in
0.2
0.4
0.6
0.8

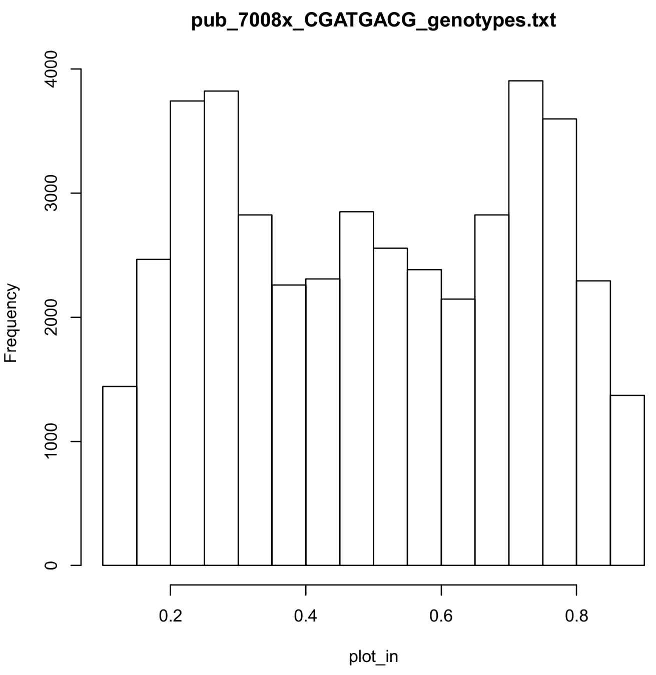
pub_7008x_CGATGACG_genotypes.txt
Frequency
0
1000
2000
3000
4000
plot_in
0.2
0.4
0.6
0.8

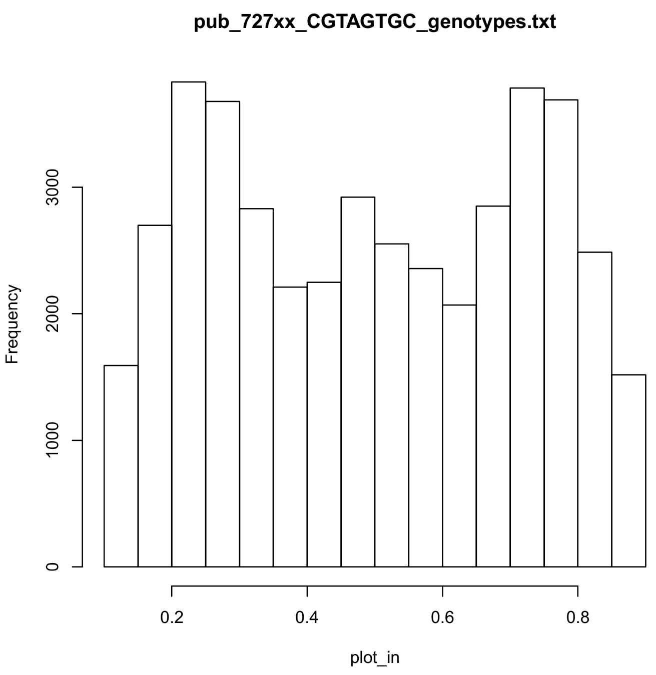
pub_727xx_CGTAGTGC_genotypes.txt
Frequency
0
1000
2000
3000
plot_in
0.2
0.4
0.6
0.8

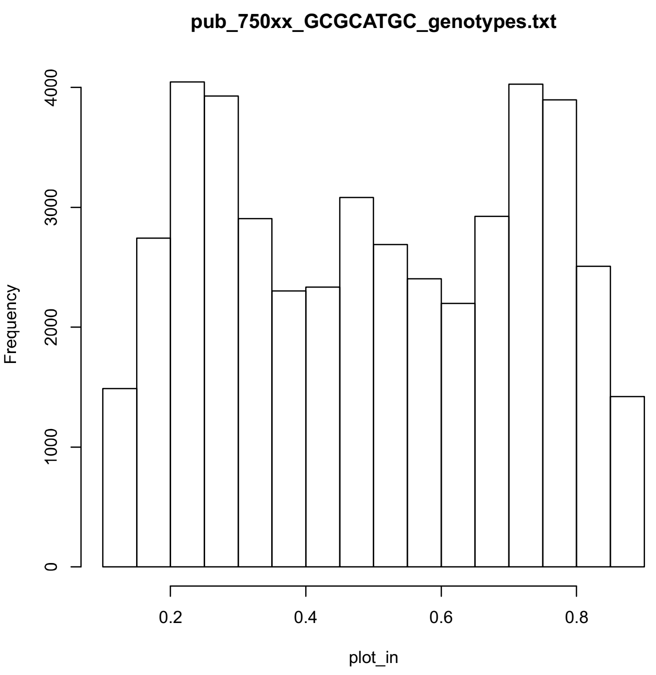
pub_750xx_GCGCATGC_genotypes.txt
Frequency
0
1000
2000
3000
4000
plot_in
0.2
0.4
0.6
0.8

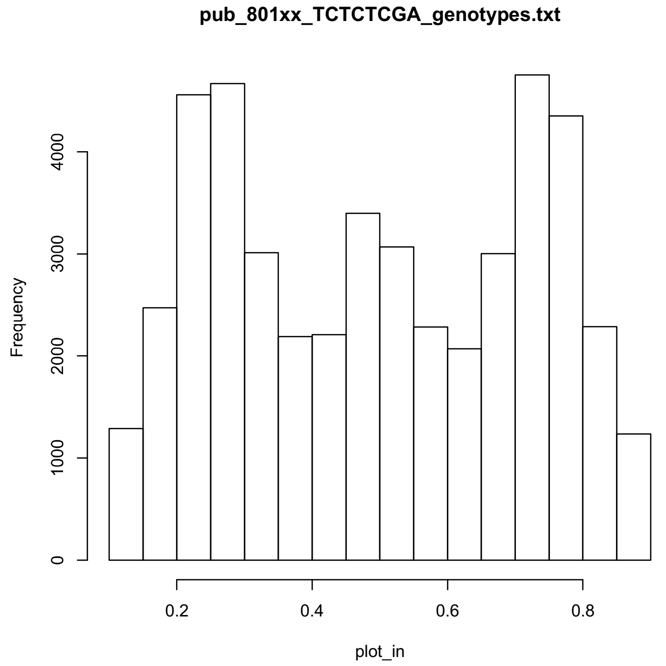
pub_801xx_TCTCTCGA_genotypes.txt
Frequency
0
1000
2000
3000
4000
0.2
0.4
0.6
0.8
plot_in

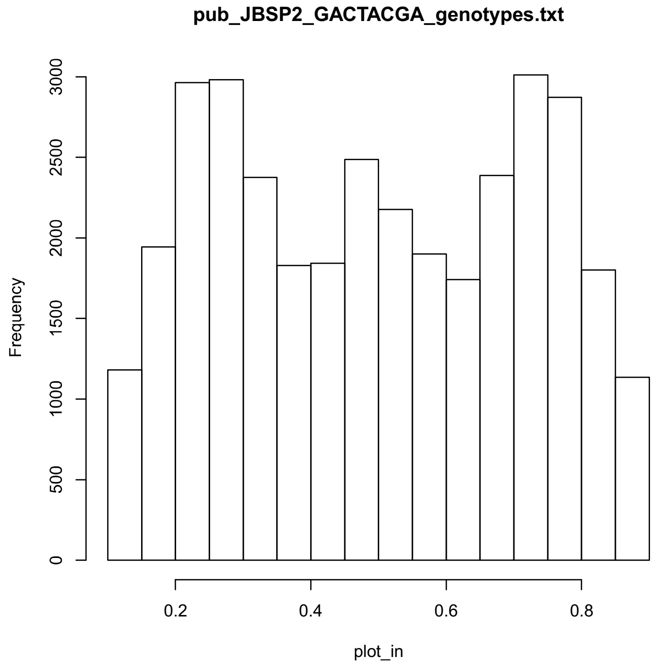
pub_JBSP2_GACTACGA_genotypes.txt
Frequency
0
500
1000
1500
2000
2500
3000
0.2
0.4
0.6
0.8
plot_in
